# *Plasmodium falciparum* rosetting protects schizonts against artemisinin

**DOI:** 10.1016/j.ebiom.2021.103680

**Published:** 2021-11-05

**Authors:** Wenn-Chyau Lee, Bruce Russell, Bernett Lee, Cindy S Chu, Aung Pyae Phyo, Kanlaya Sriprawat, Yee-Ling Lau, François Nosten, Laurent Rénia

**Affiliations:** a A*STAR Infectious Diseases Labs, Agency for Science, Technology and Research (A*STAR), Singapore; b Singapore Immunology Network (SIgN), A*STAR, Singapore; c Department of Microbiology and Immunology, University of Otago, Dunedin, Otago, New Zealand; d Shoklo Malaria Research Unit, Mahidol-Oxford Tropical Medicine Research Unit, Faculty of Tropical Medicine, Mahidol University, Mae Sot, Tak, Thailand; e Nuffield Department of Medicine, University of Oxford, United Kingdom; f Department of Parasitology, Faculty of Medicine, University of Malaya, Kuala Lumpur, Malaysia; g Lee Kong Chian School of Medicine, Nanyang Technological University, Singapore; h School of Biological Sciences, Nanyang Technological University, Singapore

**Keywords:** Artemisinin resistance, Plasmodium falciparum, rosetting, PfEMP1

## Abstract

**Background:**

Artemisinin (ART) resistance in *Plasmodium falciparum* is thought to occur during the early stage of the parasite's erythrocytic cycle. Here, we identify a novel factor associated with the late stage parasite development that contributes to ART resistance.

**Methods:**

Rosetting rates of clinical isolates pre- and post- brief (one hour) exposure to artesunate (AS, an ART derivative) were evaluated. The effects of AS-mediated rosetting on the post-AS-exposed parasite's replication and survival, as well as the extent of protection by AS-mediated rosetting on different parasite stages were investigated. The rosetting ligands, mechanisms, and gene mutations involved were studied.

**Findings:**

Brief AS exposure stimulated rosetting, with AS-resistant isolates forming more rosettes in a more rapid manner. AS-mediated rosetting enabled infected erythrocytes (IRBC) to withstand AS exposure for several hours and protected the IRBC from phagocytosis. When their rosetting ability was blocked experimentally, the post-AS exposure survival advantage by the AS-resistant parasites was abrogated. Deletions in two genes coding for PfEMP1 exon 2 (PF3D7_0200300 and PF3D7_0223300) were found to be associated with AS-mediated rosetting, and these mutations were significantly selected through time in the parasite population under study, along with the K13 mutations, a molecular marker of ART-resistance.

**Interpretation:**

Rapid ART parasite clearance is driven by the direct oxidative damages on IRBC by ART and the phagocytic destruction of the damaged IRBC. Rosetting serves as a rapid ‘buying time’ strategy that allows more parasites to complete schizont maturation, reinvasion and subsequent development into the intrinsically less ART-susceptible ring stage.

**Funding:**

A*STAR, NMRC-OF-YIRG, HRC e-ASIA, Wellcome.

**Research in context**

**Evidence before this study**

Artemisinin (ART) and its derivatives are superior to other antimalarials due to their rapid clearance of IRBC via phagocytic destruction of the IRBC by the host's immune system following rapid induction of oxidative damages to the IRBC. However, the spread of artemisinin-resistant *P. falciparum* is threatening malaria control and eradication. Currently, the mechanism of ART resistance revolves around the ‘ring (early stage)-mediated’ strategy involving several gene mutations, with the K13 mutations receiving the highest attention. According to this assumption, the mature (late) stages of ART-resistant parasites remain as ART-susceptible as the late stages of ART-sensitive parasites. Certainly, most experimental approaches investigating ART resistance solely focus on the ring stages, overlooking confounding effects arising from trophozoite- and schizont-IRBC. Recently, late stage-IRBC were reported to counter phagocytosis by forming rosettes with uninfected RBC. Late stages of *P. falciparum* that developed ART resistance *in vitro* were shown to upregulate expression of proteins involved in cytoadherence such as PfEMP1. Recently, the expression pattern of PfEMP1 in parasite population was found to be associated with the natural selection of resistance against ART and other anti-malarials. Nevertheless, the effect of ART on cytoadherence of IRBC has been neglected, with only two reports showing inhibition of cytoadherence after either long hours of drug incubation (which can substantially rigidify IRBC and compromise its cytoadherence), or exposure to the drug at the early stage (which has been proven to be the critical period for synthesis of PfEMP1). Here, we reevaluated the effects of ART on rosetting by briefly exposing the late stage-IRBC (the stage with optimal cytoadherence property) to artesunate (AS, a derivative of ART).

**Added value of this study**

Here, we show that the late stages of AS-resistant parasites formed more rosettes in a faster manner than the AS-sensitive group upon AS exposure. The AS-mediated rosetting conferred survival advantage to the AS-resistant parasites by allowing them to complete their schizogony after drug exposure and hampering of phagocytosis of IRBC by the phagocytes. The protection conferred by AS-mediated rosetting was limited to mostly the schizonts, ‘buying time’ for the parasites to replicate into ring stages, which are intrinsically less ART-susceptible, in addition to the beneficial effect conferred by mutations in the K13 gene in resistant parasites. We also provide evidence that deletions in two genes coded for different parts of PfEMP1 exon 2 are associated with AS-mediated rosetting, and that deletion events in these two genes were significantly selected through time, with deletions in one of them significantly associated with the occurrence of K13 mutations.

**Implication of all the available evidence**

Our findings complement the current picture of ART resistance development by *P. falciparum*. Attention should be given to all stages when designing ART resistance-related studies in future. The drug-mediated rosetting may be one of the initial phases facilitating the development of potent drug resistance in malaria parasites.

Alt-text: Unlabelled box

## 1 Introduction

Artemisinin (ART) was discovered just as chloroquine efficacy against malaria was failing [1]. ART is effective against all *Plasmodium* blood stages [2] via induction of oxidative damages to the infected erythrocyte (IRBC) [3], [4], [5], albeit of different potency against different developmental stages [6,7]. The ART-damaged IRBC are rapidly destroyed via the phagocytic clearance machinery of the host's immune system, resulting in fast parasite clearance by ART [8,9]. Unfortunately, ART-resistant falciparum malaria continues to emerge and is widespread in many geographical areas [10], [11], [12]. Earlier studies have shown that ART resistance manifests in the parasite's early erythrocytic (ring) stage harboring certain mutations in *PfKelch13* (K13) gene [12,13], which increase its oxidative stress resistance [14]. When such parasites are ART-treated, they reduce uptake of host hemoglobin (hence reduce heme production that activates ART) [15], trigger maturation arrest [16], and persist as the least ART-susceptible ring stages until ART (with short elimination half-live) is cleared from the circulation [17]. Based on this discovery, it was postulated that the late stages (trophozoites and schizonts) of ART-resistant parasites remained susceptible to ART [6].

Not all K13 single nucleotide polymorphisms (SNP) are significantly associated with ART resistance phenotype [18], [19], [20]. Besides, ART resistance phenotype has been shown to occur independently of K13 mutations [21,22], and delayed parasite clearance by ART treatment was reported with parasite isolates that did not harbor K13 mutations [23], [24], [25]. Therefore, ART resistance is probably mediated by multiple factors. In fact, mutations in a few other genes have been associated with ART resistance [22,[25], [26], [27]]. Nevertheless, most of the ART resistance-related studies focused on the ring stages, and all recently reported resistance-associated mutations were uncovered based on the ring-stage survival assay (RSA), which emphasizes on the survival ability of parasite ring stages after exposure to dihydroartemisinin (DHA, an active metabolite of ART with anti-malarial activity) [28,29]. It remains unknown if there are any resistance mechanisms in the intrinsically more ART-susceptible late stages, whose IRBC cytoadherence properties have long been associated with severe malaria pathogenesis [30]. Indeed, an earlier study showed that *P. falciparum in vitro* selected for ART resistance demonstrated drug resistance phenotypes at late stages [31], as reflected by their much higher IC_50_ (drug concentration that exerts schizont maturation inhibition on 50% of the parasite population [32]) for DHA.

IRBC have been found to counter phagocytosis via rosetting, a phenomenon of a late stage-IRBC stably adhering to uninfected erythrocytes (URBC)[33]. Nevertheless, reported studies that investigated ART effects and rosetting were scarce. Two earlier investigations reported that ART inhibited *P. falciparum* endothelial cytoadherence and rosetting when the parasites were exposed to ART either for hours [34], or during the early stages of the parasites [35]. However, subsequent discoveries of long ART exposure aggravating IRBC rigidity [36], negative correlation between IRBC rigidity and IRBC cytoadherence [37], and the parasite's tight temporal regulation of rosetting ligand expression [38], [39], [40], [41], [42], [43] suggest that rosetting machinery of late stage-IRBC may behave differently when exposed to ART for a shorter time. Here, we re-evaluated the effect of ART on rosetting by using brief artesunate (AS, an ART derivative used clinically as anti-malarial treatment in the area under study) exposure [for one hour (1 hr) in most of the experiments]. We also investigated the relationship between ART exposure, ART-driven IRBC phagocytosis, rosetting phenomenon, and parasite replication fitness after exposure to the drug.

## 2 Methods

Information of reagents and tools used, and the experiment workflow are available in Supplementary Table 1 and Supplementary Figure 1, respectively.

### 2.1 Study approval

Falciparum malaria-infected blood were collected in north-western Thailand by Shoklo Malaria Research Unit (SMRU) under ethical guidelines: OxTREC 04-10 (University of Oxford, UK) and TMEC 09-082 (Mahidol University, Thailand). Adult participants provided informed written consent, and a guardian provided informed written consent on behalf of juvenile participant.

### 2.2 Standard technical approaches

Each experiment was conducted with at least six biological replicates using different clinical isolates or laboratory-adapted parasite lines. We used AS in all experiments, except experiments with DHA. Parasites were cultured with human ‘O’ group RBC at 2% hematocrit, 1% parasitemia in 20% human serum enriched RPMI 1640 under *in vitro* cultivation conditions (temperature of 37°C, humidity of at least 90%, supplied with gas mixture of 5% CO_2_, 5% O_2_, 90% N_2_). Unless stated otherwise, experiments were conducted when ≥ 70% of the parasite population attained late stages (trophozoite - schizont), exposure to AS (49·42 nM) or other drugs, and co-incubation with phagocytes were done for one hour (1 hr; defined as ‘brief exposure’) at *in vitro* cultivation conditions. AS was dissolved in ethanol and transferred to 96-well flat bottom plates, and wells containing drug-free ethanol were prepared as drug-free control wells. The ethanol within all wells were allowed to evaporate completely under sterile condition. The drug-containing plates were stored under sterile condition at 4°C. Experiments involving drug exposure employed drug-free control [for experiment with DHA, DMSO (the solvent of DHA [28]) control was used]. Ratio of mixing THP-1 and RBC = 1: 10,000. Washings were done with culture media.

### 2.3 Rosetting assay

Parasites were incubated with AS (0 - 49·42 nM) [44,45] prior to rosetting assay with Giemsa-wet mount method [46,47]. Rosetting rates (percentage of IRBC stably adhered to URBC) were determined by recruiting 200 IRBC. Rosetting rate changes between control and AS-exposed groups were calculated as ‘AS-induced changes of rosetting rates’: [(rosetting rate with 49·42 nM AS – rosetting rate without AS) ÷ rosetting rate without AS] x 100%. The rosetting assay was repeated with AS of higher concentrations (494·2 nM, 4·942 µM, 49·42 µM) and DHA (700 nM).

Subsequently, experiments were designed based on AS-parasite clearance half-life (AS-PCt_1/2_), i.e. the estimated time for AS to reduce patient's parasitemia by half during the log-linear phase of parasite clearance post-AS administration [48]. Previously, the geometric mean AS-PCt_1/2_ of ‘fast’ clearance parasites was determined as 2·7 hrs, with the probability of any isolate with AS-PCt_1/2_ longer than 5·5 hrs belonging to ‘AS-sensitive’ groups was 0·043[49]. To recruit isolates into ‘fast’ and ‘slow’ clearance categories that could more certainly reflect ‘AS-sensitive’ and ‘AS-resistant’ respectively, isolates with AS-PCt_1/2_ shorter than 3 hrs were grouped as ‘short AS-PCt_1/2_’, and those with AS-PCt_1/2_ longer than 5 hrs were grouped as ‘long AS-PCt_1/2_’. Notably, of the recruited samples, all isolates with short AS-PCt_1/2_ were verified as K13 WT whereas all isolates with long AS-PCt_1/2_ were verified as K13 mutants (albeit of various K13 SNP; refer to Supplementary Table 1 for AS-PCt_1/2_ and K13 status of recruited individual isolates). The parasites were AS exposed prior to rosetting assay. A culture aliquot was kept for phagocytosis assay. Separately, rosetting rates of AS-exposed parasites were determined at interval of ten minutes from time zero to 1 hr post-AS introduction.

### 2.4 Parasite survival and replication assessment

Parasites (ring stage; <10 hrs post-invasion) were divided into four groups. One group served as control, the remaining were incubated with AS for 1, 4, and 6 hrs respectively, followed by drug removal and cultivation in drug-free media for 3 days. Parasite growth was evaluated at the intervals of 24 hrs (H_24_), 48 hrs (H_48_) and 72 hrs (H_72_) post-cultivation by preparing H_24_, H_48_, and H_72_ smears. Experiments were repeated with the early-mid trophozoite (∼ 26 – 35 hrs post-invasion), early schizont (∼ 38 – 42 hrs post-invasion) and trypsinised early schizonts. Early schizonts were used to ensure that the schizonts were yet to rupture even after the longest AS exposure. To remove the rosetting ligands from the surface of IRBC, we trypsinised (1 mg/ml, 37°C for 5 minutes, followed by addition of serum-enriched media to stop trypsin action) the schizont-IRBC [50,51], prior to mixing them with non-trypsinised URBC and subsequent AS exposure. Trypsin would not affect merozoite reinvasion since the enzyme was removed before the rupture of schizonts, and the treatment removed only the proteins (the rosetting ligands) on IRBC surface.

### 2.5 Phagocytosis assay

THP-1 cells were cultured with 10% fetal bovine serum (FBS)-enriched RPMI1640 medium. The cell line was validated as *Mycoplasma*-free (tested with MycoAlert™ PLUS kit). The THP-1 cells were primed with culture supernatant of *P. falciparum* culture (ratio of *P. falciparum* culture supernatant: culture medium = 1:4) for 24 hours prior to experiments. The AS-exposed parasites were incubated with the THP-1. Subsequently, IRBC phagocytosis rate (percentage of THP-1 with ongoing/ successful IRBC engulfment by the phagocyte; mere binding/ contact of IRBC with THP-1 without evident extension of pseudopods by the phagocyte to engulf the IRBC were not included in the counting) was determined with wet mount by recruiting 1000 THP-1^33,46^. AS-induced changes in phagocytosis were calculated: [(phagocytosis rate in ‘AS-exposed’ – phagocytosis rate in ‘AS-free’) ÷ phagocytosis rate in ‘AS-free’] x 100%.

Earlier on, THP-1 (AS-exposed vs. AS-free control) were incubated with the magnetic activated cell sorter (MACS)-purified [52] *P. falciparum* late stage-IRBC (IRBC purity ≥ 90%) for phagocytosis assay, to evaluate the effect of AS exposure on phagocytosis activity of THP-1. Separately, AS-exposed parasites (7 laboratory-adapted parasite lines were recruited) were incubated with THP-1. For each recruited parasite line, phagocytosis of 200 non-rosetting and 200 rosetting IRBC were evaluated. Numbers of phagocytes involved in each phagocytosis event were recorded. In another experiment, IRBC were MACS-purified to prevent rosetting, subsequently AS-exposed prior to THP-1 co-incubation for phagocytosis assessment.

### 2.6 Reinvasion assessment of AS-exposed parasites and THP-1 co-culture

AS-containing culture medium was removed from the AS-exposed parasites prior to THP-1-co-culture. ‘Hour zero’ (H_0_) smears were prepared. H_24_ smears were prepared 24 hrs post-culture. Replication was evaluated by deducting H_0_ late stage-parasitemia from the H_24_ ring stage-parasitemia.

### 2.7 Membrane cholesterol depletion

Two aliquots from each batch of MACS-purified IRBC were prepared. One served as control; the other was subjected to membrane cholesterol depletion with methyl-β-cyclodextrin (MBCD; 5mM) as described elsewhere [53]. The IRBC (MBCD-treated and untreated control) were mixed with untreated URBC to prepare parasite culture suspension with culture media. The baseline rosetting rates were determined. Subsequently, their rosetting responses to AS were evaluated.

### 2.8 Usage of trypsin at different concentrations to remove different rosetting ligands

Late stage-IRBC were purified with MACS and divided into three groups. One group was added with trypsin (working concentration 10 µg/ml), another group was added with trypsin (working concentration 1 mg/ml) and the last group was added with 1X PBS (as control). The cells were incubated for 5 minutes at 37°C, followed by addition of serum-enriched media to stop the enzyme activity, and centrifugation to remove the supernatant. After three rounds of washings, the packed IRBC were mixed with URBC and serum-enriched media to prepare parasite culture suspension. For each of the three groups of culture suspension, two categories were assigned. One was added with AS and the other acted as drug-free control. Rosetting assay ensued after 1 hr incubation.

### 2.9 Genes involved in AS-mediated rosetting

Culturable clinical isolates used for MalariaGEN *P. falciparum* Community Project [54] were recruited (Supplementary Table 2). With the isolates’ K13 status (WT or mutant) as reference, 140 genes associated either with development of ART resistance or expression of rosetting ligands were screened (Supplementary Table 3) [22,[25], [26], [27],^42^,[55], [56], [57], [58], [59], [60], [61], [62], [63], [64], [65], [66], [67], [68]]. Isolates were grouped based on genotypic combinations of the genes of interest to compare their AS-mediated rosette stimulation.

### 2.10 Bivariate statistics of gene polymorphisms

Genes of interest (PF3D7_1343700, PF3D7_0200300, PF3D7_0223300) in 513 isolates (MalariaGEN database) were examined (Supplementary Table 4). Relationship between deletions in PF3D7_0200300 and PF3D7_0223300, K13 mutations (gene PF3D7_1343700) and timeline (year 2010 set as cut-off) of samples collected between year 2001 and 2013 [n = 511 for timeline analysis (excluded 2 samples with missing collection date)] were evaluated using Fisher's exact tests with Bonferroni correction via R-3·3·3. Associations (visualized with Cytoscape 3.6.1.) were considered significant when P < 0·05.

### 2.11 Statistics

Analyses (except bivariate statistics) were performed with GraphPad Prism 9·0 using guidelines illustrated (Supplementary Figure 2). Comparisons were performed with two-sided testing.

### 2.12 Role of funding source

The funding source did not carry any role in study design, data collection and analyses, manuscript preparation and decision to submit the work for publication.

## 3 Results

### 3.1 Artesunate and rosetting

**Fig. 1:**
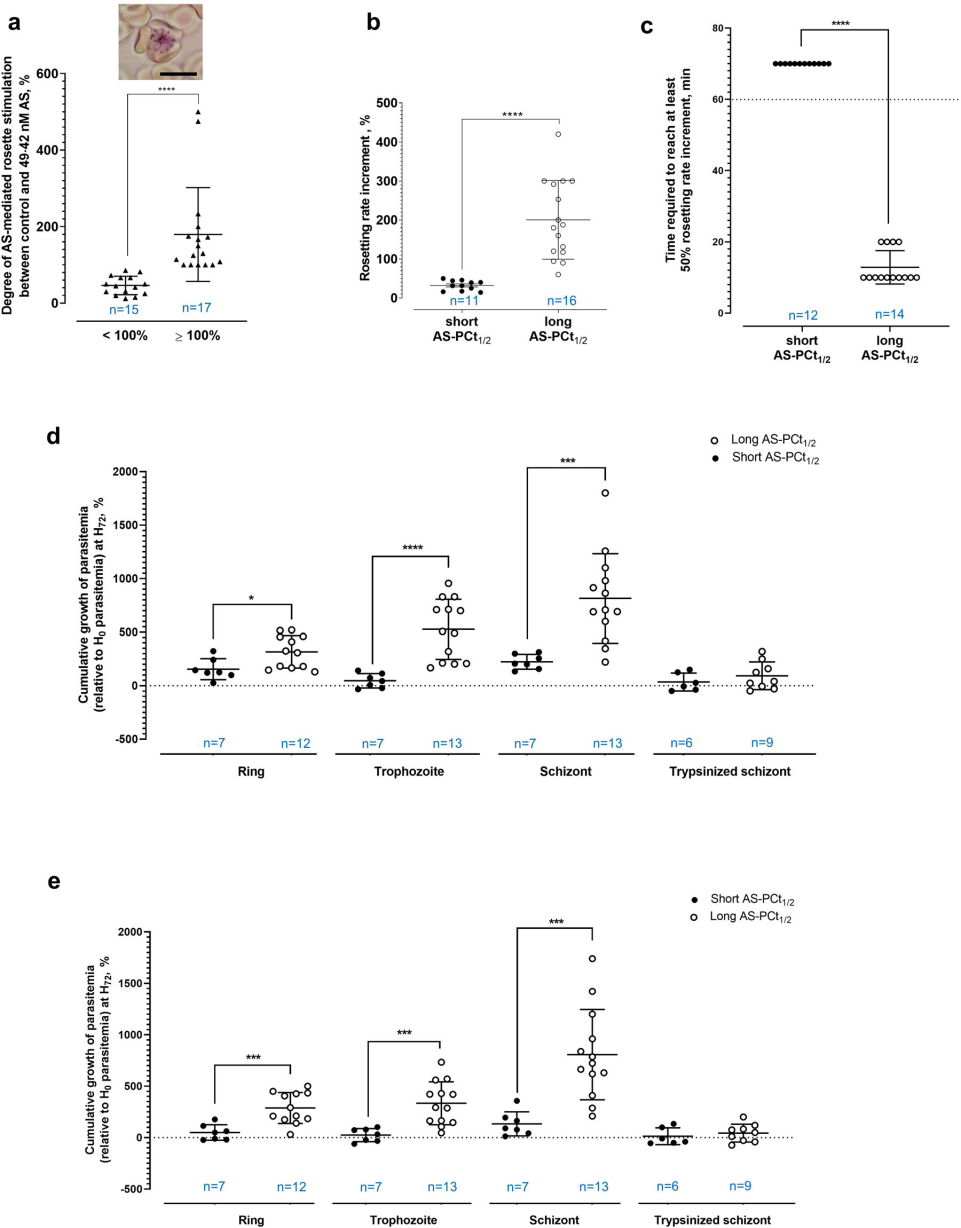
Brief AS-*P. falciparum* exposure. **(a)** The degree of increment in rosette (inset) formation post-AS exposure. AS-mediated rosette-stimulation range 12 – 500%. With the median of AS-mediated rosette-stimulation as cut-off, isolates formed two clusters (mean and S.D. shown) with different AS-mediated rosette-stimulation (Mann-Whitney P < 0·0001). **(b)** Long AS-PCt_1/2_ had higher AS-mediated rosette-stimulation (range 60 – 420%; mean 200·3%, S.D. 100·8) than short AS-PCt_1/2_ (range 14·29 – 50%; mean 32·272%, S.D. 12·73) (Mann-Whitney P < 0·0001). **(c)** Difference in the speed of AS-mediated rosetting between long- and short AS-PCt_1/2_ groups to attain at least 50% of rosetting rate increment after AS exposure. The rosetting rates of isolates were monitored at 10-minutes interval until the 60^th^ minute post-AS exposure (indicated by dotted line in the plot). As none of the isolates with short AS-PCt_1/2_ demonstrated 50% rosetting rate increment within the 60 minutes of monitoring (and demonstrated plateau trend on AS-mediated rosette-stimulation within this period, as shown by Supplementary Figure 3e), an arbitrary value of “70 minutes” were used for statistical comparison. On average, isolates with long AS-PCt_1/2_ required 12·86 ± 4·688 minutes to reach 50% increment in their rosetting rates. Significant difference was found between the two groups (Mann-Whitney P < 0·0001). **(d)** Growth of parasites (at H_72_) after 1 hr-AS exposure at different stages (ring, trophozoite and schizont) and trypsinised schizont (to prevent rosette formation by removing rosetting ligands on the surface of IRBC). Parasites with long AS-PCt_1/2_ experienced significantly higher growth than the short AS-PCt_1/2_ group (Welch's t test P = 0·0118, t = 2·825, df = 16·70; P < 0·0001, t = 5·868, df = 14·35; P = 0·0002, t = 4·964, df = 13·17 for exposure at ring, trophozoite and schizont stages, respectively). For the trypsinised schizont setting, no significant difference was found between the parasite growth of both AS-PCt1/2 groups (P = 0·3123, t = 1·051, df = 13). **(e)** Growth of parasites (at H_72_) after 6 hrs-AS exposure at different stages (ring, trophozoite and schizont) and trypsinised schizont. Parasites with long AS-PCt_1/2_ experienced significantly higher growth than the short AS-PCt_1/2_ group (Welch's t test P = 0·0003, t = 4·579, df = 16·81; P = 0·0002, t = 4·938, df = 15·57; P = 0·0001, t = 5·196, df = 14·87 for exposure at ring, trophozoite and schizont stages, respectively). For the trypsinised schizont setting, no significant difference was found between the parasite growth of both AS-PCt1/2 groups (P = 0·5035, t = 0·691, df = 11·28). Sample size (n) is stated in each plot.

By referring to the geometric means of AS IC_50_ of *P. falciparum* isolates collected from Thailand [69], [70], [71], we exposed the late stages (trophozoite - schizonts) of *P. falciparum* fresh clinical isolates collected from the north-western Thailand to AS (0 - 49·42 nM) briefly (1 hr) under *in vitro* cultivation conditions. The brief drug exposure stimulated rosette formation by the parasites. The AS-mediated rosette-stimulation ranged between 12% and 500% increment from AS-free to the highest AS concentration tested, revealing clusters of parasite isolates with different rosetting responsiveness to AS (Figure 1a), despite the overall trend of AS concentration-dependent rosette-stimulation (Supplementary Figure 3a). Considering the much higher post-administration peak serum concentration (C_max_) of AS recorded from clinical settings [72], and the availability of DHA within patients receiving ART treatment, we exposed laboratory adapted *P. falciparum* late stages to the much higher concentrations of AS (up to 49·42 µM) and DHA of 700 nM briefly, and found that their degrees of rosette-stimulation were similar to that of 49·42 nM AS (Supplementary Figure 3b).

### 3.2 AS-PCt_1/2_ and rosetting

Delayed parasite clearance in patients treated with AS in the Greater Mekong Subregion (GMS) was the first indication of parasite resistance to ART treatment [73,74]. Thus, we compared the AS-mediated rosette stimulation among clinical isolates with different AS-PCt_1/2_^48^. The AS-mediated rosette-stimulation of late stages with long AS-PCt_1/2_ was higher than those with short AS-PCt_1/2_ (Figure 1b). Remarkably, younger stages (late rings) (Supplementary Figure 3c) with long AS-PCt_1/2_ also responded to AS by forming more rosettes (Supplementary Figure 3d). In addition, parasites with long AS-PCt_1/2_ also demonstrated faster AS-mediated rosette stimulation (Figure 1c). The late stages with short AS-PCt_1/2_ required 40 minutes of AS exposure to significantly increase rosetting rates (Supplementary Figure 3e) whereas those with long AS-PCt_1/2_ required only 10 minutes of AS exposure to experience significant increment in rosetting rates (Supplementary Figure 3f). In short, isolates with long AS-PCt_1/2_ experienced more potent and rapid AS-induced rosette formation than those with short AS-PCt_1/2_.

### 3.3 Rosette protects trophozoites and schizonts with long AS-PCt_1/2_ from AS

Although ART and its derivatives have short elimination half-lives, the dosage administration of ART was designed to compensate for this drawback, even when ART monotherapy was implemented [75,76]. Therefore, we evaluated the relevance of AS-mediated rosetting in conferring survival advantage to parasites under AS exposure of different durations. Among the currently available methods used for assessment of parasite survival under drug exposure, RSA emphasizes more on the survival ability of ring stages under DHA exposure [28], whereas the IC_50_ evaluation requires long incubation (nearly 48 hrs) of parasites with the drug [32]. Here, we employed a slightly different approach. Different stages (ring, trophozoite and schizont) of parasites were exposed to AS for different durations (1, 4 and 6 hrs) prior to cultivation in a drug-free environment for another 72 hrs. The difference in parasite growth across all drug exposure durations became more obvious when the post-drug-removal cultures were maintained for longer time (Supplementary Figures 4 and 5). As expected, the ring stages of parasites with long AS-PCt_1/2_ demonstrated better growth than ring stages with short AS-PCt_1/2_ after AS exposure, whether it was as short as 1 hr-exposure (Figure 1d), or as long as 6 hrs of AS exposure (Figure 1e). The late stages (trophozoite and schizont) with long AS-PCt_1/2_, which demonstrated superior AS-mediated rosetting capability, showed better growth than their counterparts with short AS-PCt_1/2_. Nevertheless, the growth experienced by trophozoites with long AS-PCt_1/2_ after 6 hours of AS-exposure (mean 333·8 ± 208·6 %; Figure 1e) was smaller than that of 1 hr-AS exposure (mean 526·6 ± 280·4 %; Figure 1d), whereas schizonts with long AS-PCt_1/2_ maintained similar growth after AS exposure for 1hr (mean 814·5 ± 418·7%; Figure 1d) and 6 hrs (mean 806·6 ± 438·6%; Figure 1e). Of note, the survival ability of AS-exposed schizonts with short AS-PCt_1/2_ was better than their AS-exposed trophozoites. This confirmed an intrinsically lower AS susceptibility of schizonts as observed previously [77,78]. After the removal of rosetting ligands from the surface of schizont-IRBC via trypsin treatment (1 mg/ml, thus alleviated the rosetting ability of the treated IRBC [79]), the AS-exposed (in all durations tested) schizonts with long AS-PCt_1/2_ no longer experienced superior growth (Figures 1d and e; Supplementary Figures 5). In short, AS-mediated rosetting increased survival and replication success of the AS-exposed late stages, particularly the schizonts, whose survival advantage was apparent even with AS exposure as long as 6 hrs.

### 3.4 AS-mediated rosetting also protect IRBC against AS-driven phagocytosis

**Fig. 2:**
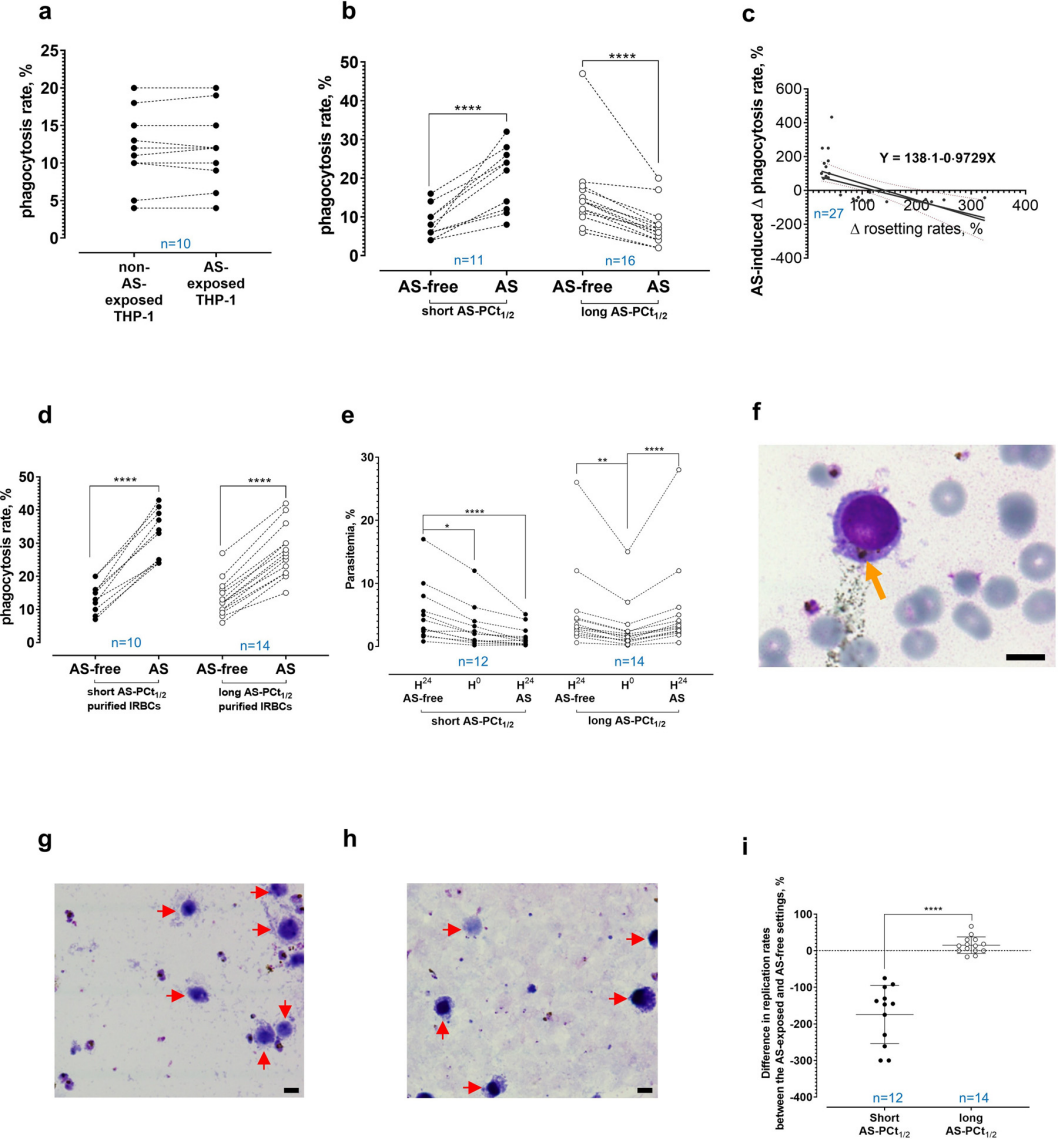
AS-mediated rosetting and phagocytosis. **(a)** AS did not alter THP-1’s ability to engulf IRBC (paired t-test P = 0·6783). **(b)** Phagocytosis increased in the AS-exposed short AS-PCt_1/2_ (paired t-test P < 0·0001). Phagocytosis decreased in the AS-exposed long AS-PCt_1/2_ (P < 0·0001). **(c)** Regression of AS-induced changes in IRBC phagocytosis and rosetting; F = 15·36, slope significantly non-zero (P = 0·0006). **(d)** Upon AS-exposure, phagocytosis of purified IRBC increased in both AS-PCt_1/2_ groups (paired t-test P <0·0001 for both). **(e)** In parasite-THP-1 co-culture, the control of short AS-PCt_1/2_ experienced increased parasitemia (Friedman test P = 0·0322), but not after AS exposure (P = 0·1574), revealing different growth between both settings (P < 0·0001). In long AS-PCt_1/2_, parasitemia increment occurred in AS-exposed setting (P < 0·0001) and control (P = 0·0028), with similar growth (P = 0·5576). **(f)** THP-1 with engulfed parasite (arrowed); thin smear. **(g)** AS-exposed short AS-PCt_1/2_ co-cultured with THP-1 (arrowed). Most parasites did not develop into rings; thick smear. **(h)** AS-exposed long AS-PCt_1/2_ co-cultured with THP-1 (arrowed), showing abundant ring forms; thick smear. **(i)** Parasite replication changes between AS-exposed and AS-free settings (mean and S.D. shown) for both AS-PCt_1/2_ groups were different (unpaired t-test with Welch's correction P < 0·0001). All images: Giemsa stained; 1000X magnification, scale bar 10 µm. Sample size (n) is stated in each plot.

As mentioned earlier, the rapid parasite clearance after ART administration is driven by the host immune system via phagocytosis of IRBC that were damaged by ART [8,9]. Recently, *P. falciparum* rosetting has been shown to protect IRBC from phagocytosis [33]. Thus, we investigated the effect of AS-induced rosetting on IRBC phagocytosis using THP-1, an acute monocytic leukemia patient-derived monocytic cell line [80], a validated surrogate for primary human monocytes/ macrophages [33]. First, we demonstrated that brief AS exposure to THP-1 did not alter their ability to phagocytose IRBC (Figure 2a). Brief AS exposure to parasite isolates with short AS-PCt_1/2_ increased phagocytosis of IRBC (Figure 2b, left panel), whereas IRBC phagocytosis rates in isolates with long AS-PCt_1/2_ decreased following AS exposure (Figure 2b, right panel). Notably, single, non-rosetting IRBC could be engulfed by individual phagocytes while engulfment of a rosette required recruitment of at least two phagocytes (Supplementary Figures 6a and b). The difference in AS-induced phagocytosis rate changes between both groups was significant (Supplementary Figure 6c). A negative linear correlation was found between AS-induced phagocytosis rate changes and AS-induced rosetting rate changes (Figure 2c). Next, late stage-IRBC were purified using MACS [52]. The scarcity of remaining URBC prevented rosetting. Here, trypsin method was not used to alleviate rosetting as we did not want to remove any IRBC surface antigen that could be used by the phagocytes for recognition and stimulation of IRBC engulfment. The AS-exposed purified IRBC from both AS-PCt_1/2_ groups were more phagocytosed than their drug-free counterparts (Figure 2d). The difference in the degree of AS-induced phagocytosis rate changes between both groups was not significant anymore (Supplementary Figure 6d), suggesting that the protection against phagocytosis on AS-exposed IRBC with long AS-PCt_1/2_ was due to the faster and higher AS-mediated rosette-stimulation (Figures 1b and c).

Subsequently, the AS-exposed late stages were co-cultured with THP-1 in drug-free medium for 24 hrs to evaluate parasite replication by deducting H_0_ late stage-parasitemia from the H_24_ ring stage-parasitemia. In the short AS-PCt_1/2_ group, the drug-free control (henceforth ‘control’) showed increased growth whereas the AS-exposed setting showed insignificant growth (Figure 2e, left panel), with many THP-1 harboring engulfed IRBC (Figure 2f). Majority of the AS-exposed late stage-parasites failed to complete schizogony (Figure 2g), reflecting their high susceptibility to AS. By contrast, the AS-exposed parasites with long AS-PCt_1/2_ grew. Ring stage-parasitemia in the control and AS-exposed settings of this group were similar (Figure 2e, right panel). Most parasites completed their schizogony and developed into rings (Figure 2h). The difference in post-AS exposure replication between the two AS-PCt_1/2_ groups was significant (Figure 2i). Apart from facilitating schizogony completion and replication under AS-containing environment, the AS-mediated rosetting also protects the late stage-IRBC from phagocytosis. Taken together, AS-mediated rosetting confers survival advantage to the parasites upon AS exposure.

### 3.5 Roles of host membrane cholesterol in AS mediated rosetting

The rosetting ability of IRBC depends on the availability of rosetting ligands on the surface of IRBC, whose IRBC surface expression becomes optimal as the parasite within the IRBC matures [39,42,43,51]. To date, several rosetting ligands have been characterized for *P. falciparum*, namely *P. falciparum* erythrocyte membrane protein 1 (PfEMP1)[81], STEVOR [82] and RIFIN [83]. These rosetting ligands have been found to be associated with a parasite-derived ‘sorting and trafficking’ organelle called Maurer's cleft [38,43,58,84], prior to IRBC surface expression. The trafficking of rosetting ligands like PfEMP1 from Maurer's cleft to IRBC surface relies on the host derived, IRBC membrane cholesterol [53].

**Fig. 3:**
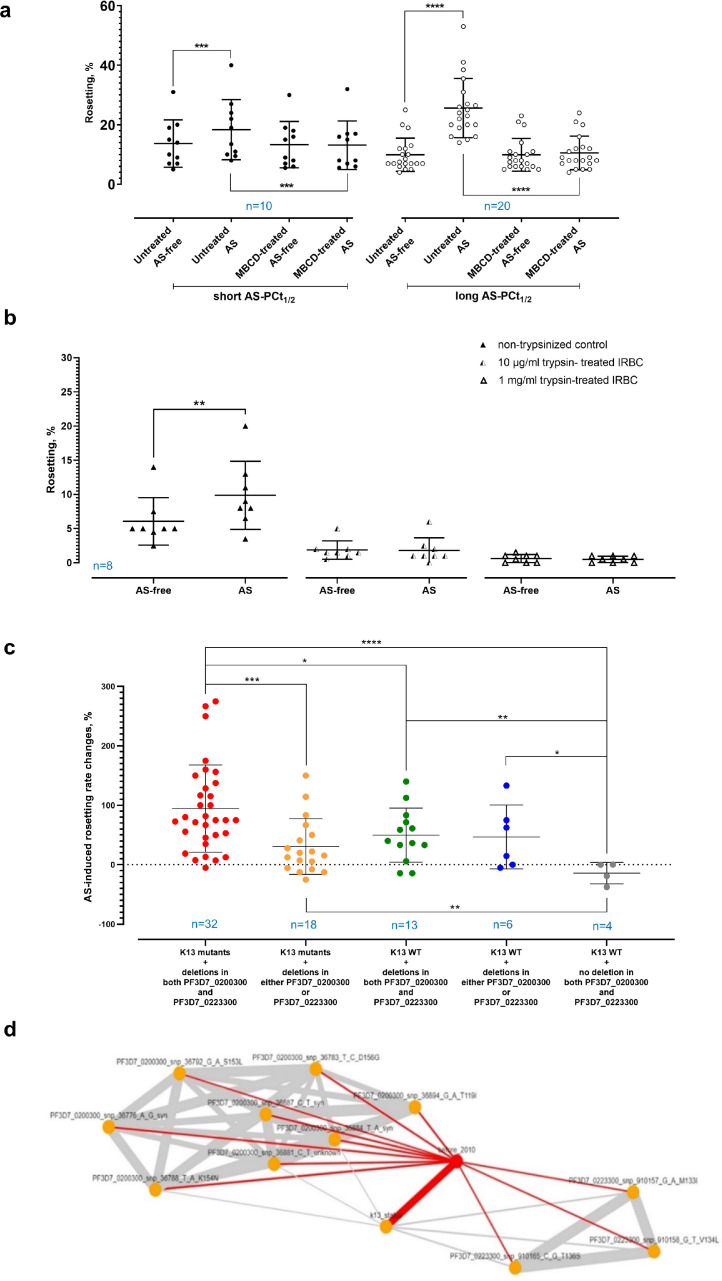
How AS mediated rosetting happens. **(a)** Via Tukey's test, MBCD treatment (membrane cholesterol depletion) did not alter baseline rosetting (P = 0·8546 and 0·9530 for short and long AS-PCt_1/2_ respectively). Without MBCD treatment, AS stimulated rosetting, albeit of different extents (P = 0·0064 for short AS-PCt_1/2_; P < 0·0001 for long AS-PCt_1/2_). After MBCD treatment, rosetting was not altered by AS (P = 0·9915 and 0·7746 for short and long AS-PCt_1/2_, respectively). **(b)** Removal of selective rosetting ligands from the IRBC surface by trypsin to decipher the ligands involved in AS-mediated rosetting. For each of the trypsin groups, Wilcoxon matched pair signed rank tests were performed to compare the rosetting rates obtained from AS-free and AS-exposed settings for each of the enzyme treatment settings. In the non-trypsinised control setting, AS significantly increased rosetting rates (P = 0·0078). For IRBC treated with 10 µg/ml and 1 mg/ml, AS did not exert significant changes to the rosetting rate (P > 0·9999 and P = 0·5000, respectively). **(c)** AS-mediated rosetting rate changes in *P. falciparum* isolates with different genotype combinations of K13, PF3D7_0223300 and PF3D7_0200300. Error bars represent mean and S.D. Each combination was represented by a distinct colour. ANOVA with two-stage linear step-up procedure of Benjamini, Krieger and Yekutieli was performed. The AS-mediated rosetting rate changes in K13 mutants with deletions in PF3D7_0200300 and PF3D7_0223300 were significantly higher than those of K13 mutants with deletions in either of the two genes (P = 0·0005), K13 WT with deletions in both genes (P = 0·0186) and K13 WT without deletions in both genes (P < 0·0001). Significant difference was found between K13 mutants with deletions in either of the two genes and K13 WT without deletions in both genes (P = 0·0076), but not with K13 WT with mutations in both genes (P = 0·2646), and K13 WT with deletions in either of the two genes (P = 0·5317). The AS-mediated rosetting rate changes in K13 WT with deletions in both genes were significantly higher than those of K13 WT without deletions in both genes (P = 0·0011). For comparison between K13 WT with deletions in either of the two genes and K13 WT without deletions in both genes, P = 0·0395. **(d)** Relationship between timeline (red lines; year 2010 as cut-off point, labelled as ‘before_2010’), K13 mutation development (‘K13_status’), deletions at SNP points of genes PF3D7_0200300 (upper left) and PF3D7_0223300 (lower right). Line thickness reflects the effect size of Cramér's V association. From Fisher's with Bonferroni tests, significant associations were found amongst deletions in PF3D7_0200300 and PF3D7_0223300 with K13 mutations (indicated by grey lines) and timeline (indicated by red lines), implying strong linkage disequilibrium. Between timeline and deletion occurrence in PF3D7_0200300, P range = 0·001 – 0·0086. Between timeline and deletion occurrence in PF3D7_0223300, P range = 0·0067 – 0·0069. Within each gene, the occurrence of deletion at different sites were strongly associated with each other. Deletions within PF3D7_0223300 were associated with the occurrence of K13 mutations (range of P = 0·0015 – 0·0029). Statistical details are available in Supplementary Table 5.

IRBC membrane cholesterol depletion with MBCD did not alter baseline (AS-free) rosetting (Figure 3a). For the MBCD-free (untreated) setting, AS stimulated rosetting in both AS-PCt_1/2_ groups, albeit of different degrees of stimulation. MBCD treatment rendered rosetting machinery of the parasites from both groups not responsive to AS, suggesting that the host membrane cholesterol-dependent trafficking machinery from Maurer's cleft to the IRBC surface might be involved in AS-mediated rosetting.

### 3.6 Rosetting ligands involved in AS-mediated rosetting

Among the identified rosetting ligands for *P. falciparum*, PfEMP1 is highly trypsin-sensitive, which can be removed from the surface of IRBC by trypsin treatment as low as 10 µg/ml, whereas STEVOR and RIFIN are relatively more resistant to trypsin treatment, requiring higher concentration of trypsin (1 mg/ml) to cleave them off the IRBC surface [50]. Thus, we employed trypsin treatment to uncover the rosetting ligands involved in AS-mediated rosetting. The non-trypsinised IRBC (control) group experienced increased rosetting rates after brief AS exposure, whereas the rosetting machinery of IRBC treated with low (10 µg/ml) and high (1 mg/ml) levels of trypsin were not responsive to AS (Figure 3b). These results support a role for PfEMP1 in AS-mediated rosetting.

### 3.7 PfEMP1 exon 2 and AS mediated rosetting

Seventy three culturable clinical isolates used for MalariaGEN *P. falciparum* Community Project [54] (whose genomes are archived in MalariaGEN open access database) were recruited (Supplementary Tables 1 and 2). By using isolates’ K13 status (WT or mutant) as reference, 140 genes reported to be associated with either ART resistance or rosetting ligands’ expression were screened (Supplementary Table 3) [22,[25], [26], [27],^42^,[55], [56], [57], [58], [59], [60], [61], [62], [63], [64], [65], [66], [67], [68],^85^]. Of these, two genes [PF3D7_0223300 (chromosomal location Pf3D7_02_v3; position 909350-911054) and PF3D7_0200300 (chromosomal location Pf3D7_02_v3; position 35927-37249), coded for different parts of PfEMP1 exon 2] showed distinct patterns between the K13 mutants and WTs. Deletions in both genes were more frequently encountered in K13 mutants, especially those collected in later years corresponding to the period with increased circulation of ART-resistant parasites in the area under study [73,86]. None of the K13 mutants was completely deletion-free in both genes of interest (Supplementary Table 2). The rosetting rates of isolates from all groups fell within the range of 2-40%, with no obvious difference in baseline rosetting rates across all groups (Supplementary Figure 7). The K13 WT without deletion in both genes of interest (Figure 3c, grey plots) did not experience rosetting rate increment following AS exposure. When comparing the AS-induced rosetting rate changes, the ‘K13 WT without deletion in both genes of interest’ group was significantly different from the K13 mutants with deletions in both genes (Figure 3c, red plots), K13 mutants with deletions in either of the two genes of interest (Figure 3c, yellow plots), K13 WT with deletions in both genes (Figure 3c, green plots), and K13 WT with deletions in either of the two genes of interest (Figure 3c, blue plots). The degree of AS-induced rosetting rate changes in K13 WT with deletions in both genes was lower than K13 mutants with deletions in both genes, but similar to those of K13 mutants and K13 WT with deletions in either of the two genes of interest.

### 3.8 Bivariate analyses

Following this, relationship between deletions in PF3D7_0200300 and PF3D7_0223300, K13 mutations (gene PF3D7_1343700) and timeline (year 2010 set as cut-off, based on the temporal establishment of widespread ART resistance in the area under study [73]) of samples collected between year 2001 and 2013 (n = 511) were evaluated (Supplementary Table 4). Deletions within PF3D7_0200300 and PF3D7_0223300 were significantly selected through the years, similar to that of K13 mutations (Figure 3e, Supplementary Table 5). Deletions in PF3D7_0200300 probably occurred independently of deletions in PF3D7_0223300 and K13 mutations, although deletion at one SNP point (position 36788) recorded a weak correlation with K13 mutations (P = 0·04). By contrast, deletions in PF3D7_0223300 were significantly associated with occurrence of K13 mutations.

## 4 Discussion

Here, we demonstrated that *P. falciparum* late stages reacted to ART (as assessed with ART derivatives AS and DHA) by forming more rosettes. We further showed that after AS exposure, the parasite isolates with slow clearance increased their rosetting rates to a greater extent than those with fast clearance. The quantity and rate of AS-mediated rosette-stimulation significantly impacted reinvasion success and IRBC susceptibility to phagocytosis. Phagocytosis depends on several factors such as the size and rigidity of targeted entities [87], [88], [89]. ART causes rapid oxidative damages to IRBC, rendering the IRBC more rigid [36], which increases their susceptibility to phagocytosis [87]. When late stage-IRBC form rosettes, the larger size of a rosette, and the masking of *Plasmodium*-derived antigens on the surface of IRBC with host RBC inhibit the phagocytosis of IRBC [88]. Although rosettes are more rigid than non-rosetting IRBC [90], the size factor is likely to predominate in this context, since engulfing a target requires a mandatory decrease in the phagocyte's plasma membrane area with a concomitant increase in its cellular volume [89]. Our study certainly shows that engulfment of rosetting IRBC is difficult, if not impossible for a single phagocyte. Thus, this study supports the idea that protection by rosetting against ART-facilitated phagocytosis confers a significant parasite survival advantage.

ART-stimulated rosetting also allows more late stage-parasites to complete schizogony following drug exposure. The higher and more rapid AS-mediated rosetting response by the late stages with long AS-PCt_1/2_ enabled them to survive through AS exposure. Among the late stages, the survival benefit conferred by AS-mediated rosetting on schizonts was more obvious. This is probably due to the fact that schizonts require shorter time to complete the maturation and reinvade new URBC to form the intrinsically less ART-susceptible ring stages. Thus, such ‘buy time’ strategy works better on the more terminally developed schizont stage. Based on our findings, terms like ‘post AS-exposure survival’ and ‘post AS-exposure replication’ regarding parasite's drug sensitivity may not be used interchangeably. Schizonts could survive brief AS exposure and replicate into rings regardless of their K13 mutation status. However, we observed that most of the 2^nd^ generation by the AS-sensitive parasites could not progress and replicate into subsequent cycle, implying that such schizonts that ‘survived the drug exposure’ were still susceptible to the drug. Here, the faster and higher AS-mediated rosette-stimulation suggests a more effective shield-like protection against intracellular accumulation of AS.

At molecular level, we provided evidence that PfEMP1 is the rosetting ligand involved in AS-mediated rosetting. Upon removal of PfEMP1 from the surface of IRBC with trypsin as low as 10 µg/ml, the rosetting rates of the IRBC were not significantly altered by AS exposure. This also suggests that STEVOR and RIFIN, the other two rosetting ligands that are more trypsin-resistant than PfEMP1, are less likely to be involved in AS-mediated rosetting. Besides, our observation of late rings with long AS-PCt_1/2_ responded to AS by forming more rosettes further supported involvement of PfEMP1 in AS-mediated rosetting, since PfEMP1 is the only rosetting ligand that is IRBC surface-expressed at such early stage [38], [39], [40], [41], [42], [43]. Of note, the immediate rosette-stimulation following brief AS exposure is unlikely to be attributed to a faster synthesis of PfEMP1, since these proteins require hours to traverse multiple membrane barriers before reaching the IRBC surface [39,55]. Interestingly, parasite-derived IRBC membrane-associated proteins are assembled in Maurer's cleft before being integrated with the IRBC plasma membrane [42,55,56]. However, the strict temporal regulation of expression causes PfEMP1 that missed the narrow period of surface expression to be retained in Maurer's cleft [39], making this parasite organelle a PfEMP1 reservoir in the cytoplasm of IRBC. Depletion of the IRBC plasma membrane cholesterol, which hampered Maurer's cleft-IRBC surface trafficking, rendered the parasite's rosetting machinery non-responsive to AS. Hence, Maurer's cleft may be the PfEMP1 reservoir for rapid IRBC surface expression upon AS exposure.

ART resistance has been associated with upregulation of parasite's protein post-translational translocation [16]. However, we do not know how ART signals rapid trafficking of PfEMP1 onto the IRBC surface, and how this phenomenon is mediated by PfEMP1 exon 2. Notably, PfEMP1 exon 2 is the cytoplasmic domain of PfEMP1 that is semi-conserved [60,91]. Deletions in genes coded for PfEMP1 exon 2 may allow bypassing of temporal regulation for PfEMP1 surface expression upon sensing threats. Interestingly, the independently occurring deletions in PF3D7_0200300 and PF3D7_0223300 seem to synergistically facilitate the IRBC to rosette more upon drug encounter. Of note, an evolutionary analysis on large exon 1 sequences of *P. falciparum var* genes has revealed high level of full-length sequence sharing among the Southeast Asian isolates that may have been resulted from the selection process of parasites resistant to ART and other anti-malarials, and the selection process was believed to be an ongoing process when the study was conducted [92]. In fact, we noticed similar trend when analyzing 33 genes related to PfEMP1 exon 1 (Supplementary Table 3). This earlier evolutionary study on *var* gene did not look into genes coded for PfEMP1 exon 2[92]. Nevertheless, by analyzing these genes, our study further proved the association of PfEMP1 and the selection of ART resistance phenotypes in the parasite population. Notably, another earlier study revealed that the *in vitro*-selected DHA-resistant *P. falciparum* demonstrated upregulated expression of several genes coded for PfEMP1 (fold change of 12·4 at ring stage), KAHRP (fold change of 8·7, 3·1 and 2·2 at ring, trophozoite and schizont stages, respectively) and the PF3D7_0200300 gene coded for PfEMP1 exon 2 (fold change of 9·7 at ring stage)[31], which agreed well with our findings. In addition, a recent population genetic study also reported association of PfEMP1 with development of ART resistance [68]. Clearly, there should be other factors within the K13 mutants with deletions in both genes of interest that enable better AS mediated rosetting ability than their K13 WT counterparts. Such factors can be mutations in other genes, or other biological attributes of the K13 mutant-IRBC under AS exposure, such as the IRBC oxidative damage level that can affect the rheology and cytoadherence properties of IRBC [36].

**Fig. 4:**
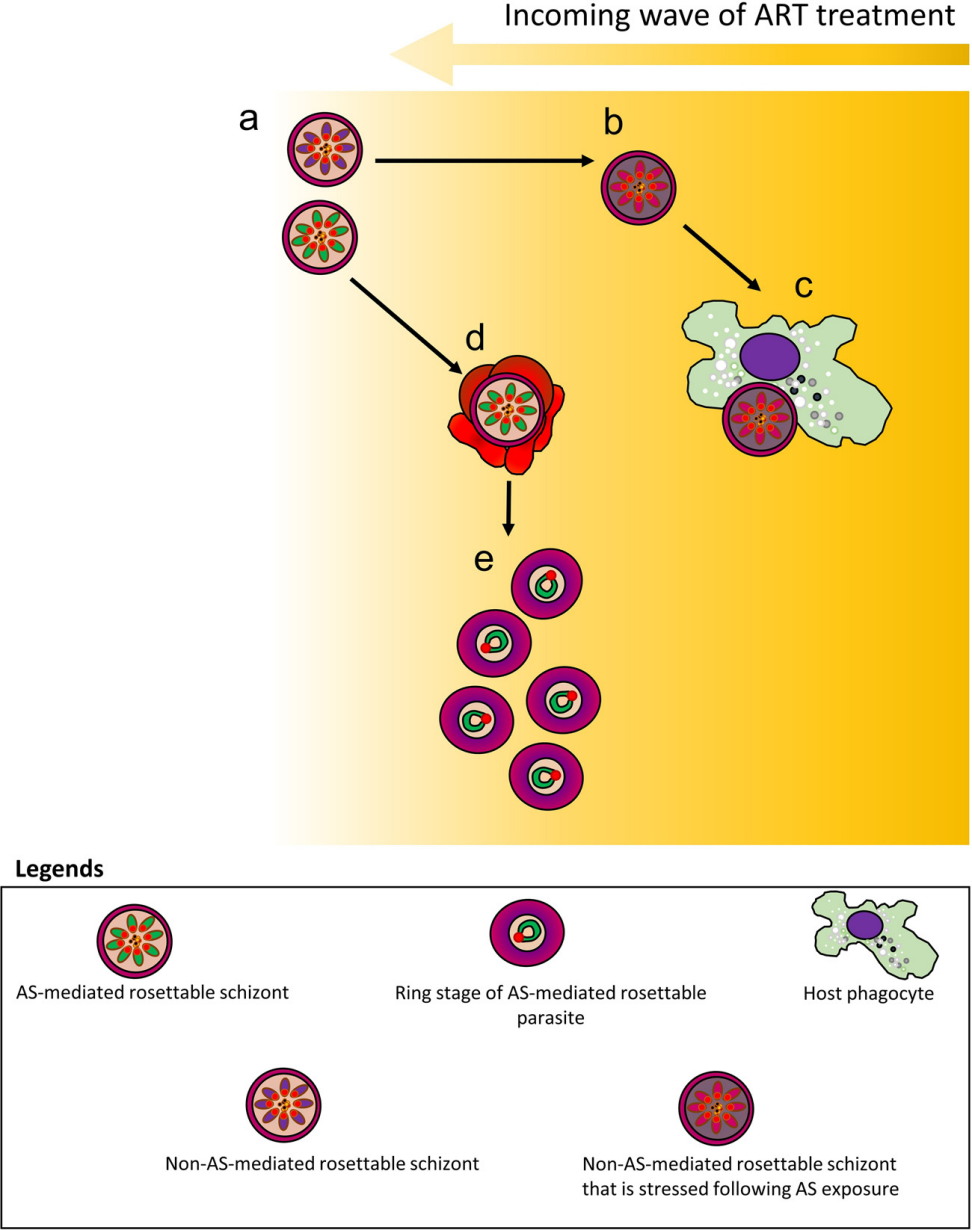
Hypothesized mechanisms of *P. falciparum* rosetting against ART. **(a)** Schizont stage-IRBCs with different rosetting phenotypes are exposed to ART. **(b)** Although not immediately killed by ART directly, the schizont-IRBC that cannot be stimulated to form rosette upon drug exposure will experience increased cellular stress induced by the drug. **(c)** Following this, the host phagocyte will easily engulf such stressed IRBC, leading to effective parasite clearance. **(d)** Schizont-IRBC that can form rosette upon the drug exposure has lower chance of being engulfed by the phagocytes, and these rosettable IRBC can resist AS-induced stress better. **(e)** As a result, these schizonts can complete their schizogony, producing many ring stage-IRBCs that are less susceptible to the drug.

Protection against ART by rosetting benefits schizonts the most. Without other ring-mediated ART-resistance strategies, the reinvaded second-generation parasites remain susceptible to a complete course of ART. Nevertheless, rosetting may contribute to drug resistance via mechanisms that we put forward. Owing to their good lipid permeability, ART and its derivatives can rapidly reach the intracellular parasites [93] (Figure 4a). ART imparts substantial cellular stress to the IRBC (Figure 4b) and increases IRBC susceptibility to phagocytosis (Figure 4c). However, some late stage-IRBC rosette more rapidly upon ART exposure (Figure 4d). Rosettes hamper IRBC phagocytosis. This ‘buys time’ for schizonts to replicate into the intrinsically less ART-susceptible rings (Figure 4e), which develop other mechanisms to survive. Although the postulated role of rosetting in enhancing merozoite reinvasion has been challenged [94], rosetting may grant instant URBC supply for *P. falciparum* merozoite invasion in the drug-containing environment. The proposed mechanism potentially benefits the parasites when drugs with short elimination half-lives are misused (e.g. substandard antimalarials or not used as prescribed)[95].

In previous studies, ART was reported to inhibit rosetting [34,35]. Experimental designs in these earlier studies were different from ours. AS exposure of only one hour was done in most of our experiments (simulating the *in vivo* kinetics of AS) instead of the longer incubation used in the earlier studies. The longer, non-physiological exposure of these earlier studies allows AS/ART to substantially harm IRBC rheology and its cytoadherence capabilities [36]. Apart from a few experiments on early-stage parasites, we did not expose ring stage to the drug as this is the critical period of the temporal-specific synthesis of *P. falciparum* rosetting ligands, which can be disrupted by the drug. Even hours after the drug is removed, expression of rosetting ligands may not resume due to their tight regulation of expression. Hence, it is not surprising that earlier study designs gave rise to different outcomes. Of note, one of these earlier studies reported that 4 hrs of incubation with anti-malarials such as ART reduced the rosetting rates of *P. falciparum* trophozoites [34]. In fact, our long duration AS-exposure (4 and 6 hrs) on trophozoites showed that the parasite growth from both long- and short AS-PCt_1/2_ groups were significantly lower than their respective drug-free controls (Supplementary Figure 4), which agreed well with this previous study. This also implies that the ART-mediated rosetting serves as an immediate strategy for the more terminally matured stages to complete schizogony. Based on our findings, drug-induced rosetting phenomenon may be one of the initial factors contributing to the development of AS resistance in some *P. falciparum*. More work is needed to decipher the detailed molecular mechanism involved in this AS-induced rosetting phenomenon. For example, gene editing involving replacement of deletion-free PF3D7_0200300 and PF3D7_0223300 genes in *P. falciparum* with edited ‘versions’ harboring different degrees of deletions, as well as complementation of parasite having deletions in the genes of interest with deletion-free replacements, should be done to test the effect of changes in these genes on the AS-mediated rosetting response. Subsequently, the phenotypic effects (rosetting, phagocytosis and AS IC_50_) arising from interactions between mutations on PF3D7_0200300, PF3D7_0223300 and *Pf*K13 should be examined further. In addition, it is worthwhile to investigate if rosetting phenomenon hampers cross-membrane diffusion and accumulation of ART in the intracellular parasite. Clearly, the mechanisms of ART resistance development in *P. falciparum* are far more complex than anticipated.

## 5 Contributors

WL, LR and BR conceived the project. WL conceptualized and planned the study. WL and KS prepared, managed and conducted *in vitro* experiments. BL performed bivariate gene analyses. FN, APP, CSC, and KS involved in collection and management of clinical data. FN, YL, APP and CSC involved in management of fieldwork, clinical management of patients, ethical clearance, collection, and processing of blood samples. WL, BL, BR, LR and FN compiled, analyzed and interpreted the collected data. WL, BL, BR, LR, CSC and FN verified the compiled and interpreted data. WL, BL, FN, CSC, BR and LR involved in manuscript preparation. All authors read and approved the final manuscript.

## Declaration of Competing Interest

We declare no competing interests.

## Data Availability

This study used genomic data from MalariaGEN database, whose sequencing was performed by the Wellcome Trust Sanger Institute. The Community Projects is coordinated by the MalariaGEN Resource Centre with funding from Wellcome Trust (098051, 090770). Genomic data from MalariaGEN P. falciparum Community Project are publicly available at www.malariagen.net/projects/p-falciparum-community-project.

## Appendix Supplementary materials

Image, application 1

Image, application 2

Image, application 3
