# Supplementary material for: Plasmodium falciparum rosetting protects schizonts against artemisinin
Source: eBioMedicine. 2021 Nov 5;73:103680. doi: 10.1016/j.ebiom.2021.103680 (PMC8586750; doi:10.1016/j.ebiom.2021.103680)
Supplement: Supplementary file 1 [file mmc1.docx]

**Supplementary materials**

| **Content** | **Page** |
| --- | --- |
| Supplementary Table 1 | 1-25 |
| Supplementary Table 2 | 26 |
| Supplementary Table 3 | 27-32 |
| Supplementary Table 4 | 33-40 |
| Supplementary Table 5 | 41* |
| Supplementary Figure 1 | 42 |
| Supplementary Figure 2 | 43 |
| Supplementary Figure 3 | 44 |
| Supplementary Figure 4 | 45 |
| Supplementary Figure 5 | 46 |
| Supplementary Figure 6 | 47 |
| Supplementary Figure 7 | 48 |

***Provided separately as excel file**

***Supplementary Table 1*. Reagents and Tools Table.**

| **Reagent type (species) or resource** | **Designation** | **Source or reference** | **Identifiers** | **Additional information** |
| --- | --- | --- | --- | --- |
| Biological sample (*Plasmodium falciparum*) | 3D7 | BEI Resources | MRA102 | Laboratory-adapted parasite strain; maintained in Singapore Immunology Network (SIgN)  Used in experiments for figures 2a, 3b, S3b |
| Biological sample (*Plasmodium falciparum*) | FVT402 | Lee et al.2020. doi: 10.7554/3Life.51546 |  | Adapted from clinical isolate in Shoklo Malaria Research Unit (SMRU)  Used in experiments for figures 2a, 3b, S3b, S6a |
| Biological sample (*Plasmodium falciparum*) | FVT201 | Lee et al.2020. doi: 10.7554/3Life.51546 |  | Adapted from clinical isolate in SMRU  Used in experiments for figures 3b, S3b, S6a |
| Biological sample (*Plasmodium falciparum*) | MKK183 | Lee et al.2020. doi: 10.7554/3Life.51546 |  | Adapted from clinical isolate in SMRU  Used in experiments for figures 3b, S3b, S6a |
| Biological sample (*Plasmodium falciparum*) | WPP3065 | Lee et al.2020. doi: 10.7554/3Life.51546 |  | Adapted from clinical isolate in SMRU  Used in experiments for figures 3b, S3b, S6a |
| Biological sample (*Plasmodium falciparum*) | WPP2803 | Lee et al.2020. doi: 10.7554/3Life.51546 |  | Adapted from clinical isolate in SMRU  Used in experiments for figures 3b, S3b, S6a |
| Biological sample (*Plasmodium falciparum*) | NHP1106 | Lee et al.2020. doi: 10.7554/3Life.51546 |  | Adapted from clinical isolate in SMRU  AS-PCt_1/2_ = 1.71 hr; K13_WT  Date of collection: Oct 2008  Used in experiments for figures 1b, 1c, 1d, 1e, 2b, 2c, 2d, 2e, 2i, 3a, 3b, S3b, S3d, S3e, S4a, S4c, S5a, S5c, S6a, S6c, S6d |
| Biological sample (*Plasmodium falciparum*) | NHP4770 | Lee et al.2020. doi: 10.7554/3Life.51546 |  | Adapted from clinical isolate in SMRU  AS-PCt_1/2_ = 8.07 hr; K13_A675V  Date of collection: Feb 2012  Used in experiments for figures 1b, 1d, 1e, 2a, 2b, 2c, 2d, 2e, 2i, 3a, 3b, S3b, S3d, S4b, S4d, S5b, S5d, S6a, S6c, S6d |
| Biological sample (*Plasmodium falciparum*) | PID412725 | This paper |  | Fresh clinical isolate from the North western part of Thailand  Used in experiments for figures 1a, S3a |
| Biological sample (*Plasmodium falciparum*) | PID405104 | This paper |  | Fresh clinical isolate from the North western part of Thailand  Used in experiments for figurea1a, S3a |
| Biological sample (*Plasmodium falciparum*) | PID403525 | This paper |  | Fresh clinical isolate from the North western part of Thailand  Used in experiments for figures 1a, S3a |
| Biological sample (*Plasmodium falciparum*) | PID109053 | This paper |  | Fresh clinical isolate from the North western part of Thailand  Used in experiments for figures 1a, S3a |
| Biological sample (*Plasmodium falciparum*) | PID102938 | This paper |  | Fresh clinical isolate from the North western part of Thailand  Used in experiments for figures 1a, S3a |
| Biological sample (*Plasmodium falciparum*) | PID402420 | This paper |  | Fresh clinical isolate from the North western part of Thailand  Used in experiments for figures 1a, S3a |
| Biological sample (*Plasmodium falciparum*) | PID402635 | This paper |  | Fresh clinical isolate from the North western part of Thailand  Used in experiments for figures 1a, S3a |
| Biological sample (*Plasmodium falciparum*) | PID412788 | This paper |  | Fresh clinical isolate from the North western part of Thailand  Used in experiments for figures 1a, S3a |
| Biological sample (*Plasmodium falciparum*) | PID305649 | This paper |  | Fresh clinical isolate from the North western part of Thailand  Used in experiments for figures 1a, S3a |
| Biological sample (*Plasmodium falciparum*) | PID412890 | This paper |  | Fresh clinical isolate from the North western part of Thailand  Used in experiments for figures 1a, S3a |
| Biological sample (*Plasmodium falciparum*) | PID404033 | This paper |  | Fresh clinical isolate from the North western part of Thailand  Used in experiments for figures 1a, S3a |
| Biological sample (*Plasmodium falciparum*) | PID412895 | This paper |  | Fresh clinical isolate from the North western part of Thailand  Used in experiments for figures 1a, S3a |
| Biological sample (*Plasmodium falciparum*) | PID412920 | This paper |  | Fresh clinical isolate from the North western part of Thailand  Used in experiments for figures 1a, S3a |
| Biological sample (*Plasmodium falciparum*) | PID404184 | This paper |  | Fresh clinical isolate from the North western part of Thailand  Used in experiments for figures 1a, S3a |
| Biological sample (*Plasmodium falciparum*) | PID412962 | This paper |  | Fresh clinical isolate from the North western part of Thailand  Used in experiments for figures 1a, S3a |
| Biological sample (*Plasmodium falciparum*) | PID412977 | This paper |  | Fresh clinical isolate from the North western part of Thailand  Used in experiments for figures 1a, S3a |
| Biological sample (*Plasmodium falciparum*) | PID401322 | This paper |  | Fresh clinical isolate from the North western part of Thailand  Used in experiments for figures 1a, S3a |
| Biological sample (*Plasmodium falciparum*) | PID409347 | This paper |  | Fresh clinical isolate from the North western part of Thailand  Used in experiments for figures 1a, S3a |
| Biological sample (*Plasmodium falciparum*) | PID411792 | This paper |  | Fresh clinical isolate from the North western part of Thailand  Used in experiments for figures 1a, S3a |
| Biological sample (*Plasmodium falciparum*) | PID402127 | This paper |  | Fresh clinical isolate from the North western part of Thailand  Used in experiments for figures 1a, S3a |
| Biological sample (*Plasmodium falciparum*) | PID413003 | This paper |  | Fresh clinical isolate from the North western part of Thailand  Used in experiments for figures 1a, S3a |
| Biological sample (*Plasmodium falciparum*) | PID109190 | This paper |  | Fresh clinical isolate from the North western part of Thailand  Used in experiments for figures 1a, S3a |
| Biological sample (*Plasmodium falciparum*) | MMA30037 | This paper |  | Fresh clinical isolate from the North western part of Thailand  Used in experiments for figures 1a, S3a |
| Biological sample (*Plasmodium falciparum*) | NHP3219 | This paper |  | Fresh clinical isolate from the North western part of Thailand  Used in experiments for figures 1a, S3a |
| Biological sample (*Plasmodium falciparum*) | MMA30038 | This paper |  | Fresh clinical isolate from the North western part of Thailand  Used in experiments for figures 1a, S3a |
| Biological sample (*Plasmodium falciparum*) | PID413024 | This paper |  | Fresh clinical isolate from the North western part of Thailand  Used in experiments for figures 1a, S3a |
| Biological sample (*Plasmodium falciparum*) | MMA40095 | This paper |  | Fresh clinical isolate from the North western part of Thailand  Used in experiments for figures 1a, S3a |
| Biological sample (*Plasmodium falciparum*) | PID413073 | This paper |  | Fresh clinical isolate from the North western part of Thailand  Used in experiments for figures 1a, S3a |
| Biological sample (*Plasmodium falciparum*) | PID412793 | This paper |  | Fresh clinical isolate from the North western part of Thailand  Used in experiments for figures 1a, S3a |
| Biological sample (*Plasmodium falciparum*) | PID109262 | This paper |  | Fresh clinical isolate from the North western part of Thailand  Used in experiments for figures 1a, S3a |
| Biological sample (*Plasmodium falciparum*) | MMA30040 | This paper |  | Fresh clinical isolate from the North western part of Thailand  Used in experiments for figures 1a, S3a |
| Biological sample (*Plasmodium falciparum*) | PID413071 | This paper |  | Fresh clinical isolate from the North western part of Thailand  Used in experiments for figures 1a, S3a |
| Biological sample (*Plasmodium falciparum*) | OHP590 | This paper |  | Clinical isolate from the North western part of Thailand  Date of collection: Jan 2002  AS-PCt_1/2_ = 1.23 hr; K13_WT  Used in experiments for figures 1b, 1c, 2b, 2c, 2d, 2e, 2i, 3a, S3d, S3e, S6c, S6d |
| Biological sample (*Plasmodium falciparum*) | OHP602 | This paper |  | Clinical isolate from the North western part of Thailand  AS-PCt_1/2_ = 1.04 hr; K13_WT  Date of collection: Mar 2002  Used in experiments for figures 1b, 1c, 2b, 2c, 2d, 2e, 2i, S3d, S3e, S6c, S6d |
| Biological sample (*Plasmodium falciparum*) | OHP646 | This paper |  | Clinical isolate from the North western part of Thailand  AS-PCt_1/2_ = 1.58 hr; K13_WT  Date of collection: Jul 2002  Used in experiments for figures 1b, 1c, 2b, 2c, 2d, 2e, 2i, 3a, S3d, S3e, S6c, S6d |
| Biological sample (*Plasmodium falciparum*) | NHP1510 | This paper |  | Clinical isolate from the North western part of Thailand  AS-PCt_1/2_ = 6.9 hr; K13_C580Y  Date of collection: Apr 2004  Used in experiments for figure 3c, 3d, S7a |
| Biological sample (*Plasmodium falciparum*) | OHP319 | This paper |  | Clinical isolate from the North western part of Thailand  AS-PCt_1/2_ = 1.9 hr; K13_WT  Date of collection: Jun 2004  Used in experiments for figures 1b, 1c, 1d, 1e, 2a, 2b, 2c, 2d, 2e, 2i, 3a, S3d, S3e, S4a, S4c, S5a,S6c, S6d |
| Biological sample (*Plasmodium falciparum*) | NHP2019 | This paper |  | Clinical isolate from the North western part of Thailand  AS-PCt_1/2_ = 3.54 hr; K13_WT  Date of collection: Mar 2008  Used in experiments for figure 3c, 3d, S7a |
| Biological sample (*Plasmodium falciparum*) | NHP1039 | This paper |  | Clinical isolate from the North western part of Thailand  AS-PCt_1/2_ = 6.14 hr; K13_P527H  Date of collection: Apr 2008  Used in experiments for figure 1c, 3a, 3c, 3d, S3f, S7a |
| Biological sample (*Plasmodium falciparum*) | NHP2037 | This paper |  | Clinical isolate from the North western part of Thailand  AS-PCt_1/2_ = 1.82 hr; K13_WT  Date of collection: May 2008  Used in experiments for figures 1b, 1c, 1d, 1e, 2b, 2c, 2d, 2e, 2i, 3a, S3d, S3e, S4a, S4c, S5a, S5c, S6c, S6d |
| Biological sample (*Plasmodium falciparum*) | NHP1053 | This paper |  | Clinical isolate from the North western part of Thailand  AS-PCt_1/2_ = 2.00 hr; K13_WT  Date of collection: May 2008  Used in experiments for figure 1c, 2e, 2i, 3c, 3d, S3e, S7a |
| Biological sample (*Plasmodium falciparum*) | NHP1059 | This paper |  | Clinical isolate from the North western part of Thailand  AS-PCt_1/2_ = 1.85 hr; K13_WT  Date of collection: Jun 2008  Used in experiments for figure 1c, 2e, 2i, 3a, 3c, 3d, S3e, S7a |
| Biological sample (*Plasmodium falciparum*) | NHP1060 | This paper |  | Clinical isolate from the North western part of Thailand  AS-PCt_1/2_ = 2.27 hr; K13_WT  Date of collection: Jul 2008  Used in experiments for figure 1c, 3c, 3d, S3e, S7a |
| Biological sample (*Plasmodium falciparum*) | NHP1068 | This paper |  | Clinical isolate from the North western part of Thailand  AS-PCt_1/2_ = 2.08 hr; K13_WT  Date of collection: Jul 2008  Used in experiments for figure 1c, 3a, 3c, 3d, S3e, S7a |
| Biological sample (*Plasmodium falciparum*) | NHP1069 | This paper |  | Clinical isolate from the North western part of Thailand  AS-PCt_1/2_ = 3.2 hr; K13_WT  Date of collection: Jul 2008  Used in experiments for figure 3c, 3d, S7a |
| Biological sample (*Plasmodium falciparum*) | NHP4265 | This paper |  | Clinical isolate from the North western part of Thailand  AS-PCt_1/2_ = 1.86 hr; K13_WT  Date of collection: Jul 2008  Used in experiments for figures 1b, 2a, 2b, 2c, S3d, S6c |
| Biological sample (*Plasmodium falciparum*) | NHP1070 | This paper |  | Clinical isolate from the North western part of Thailand  AS-PCt_1/2_ = 2.44 hr; K13_WT  Date of collection: Jul 2008  Used in experiments for figure 3c, 3d, S7a |
| Biological sample (*Plasmodium falciparum*) | NHP1071 | This paper |  | Clinical isolate from the North western part of Thailand  AS-PCt_1/2_ = 5.37 hr; K13_P527H  Date of collection: Jul 2008  Used in experiments for figures 3c, 3d, S7a, S7b |
| Biological sample (*Plasmodium falciparum*) | NHP3048 | This paper |  | Clinical isolate from the North western part of Thailand  AS-PCt_1/2_ = 1.91 hr; K13_WT  Date of collection: Aug 2008  Used in experiments for figures 1b, 1c, 1d, 1e, 2a, 2b, 2c, 2d, 2e, 2i, 3a, S3d, S3e, S4a, S4c, S5a, S5c, S6c, S6d |
| Biological sample (*Plasmodium falciparum*) | NHP1088 | This paper |  | Clinical isolate from the North western part of Thailand  AS-PCt_1/2_ = 2.60 hr; K13_WT  Date of collection: Sep 2008  Used in experiments for figure 3a, 3c, 3d, S7a |
| Biological sample (*Plasmodium falciparum*) | NHP1095 | This paper |  | Clinical isolate from the North western part of Thailand  AS-PCt_1/2_ = 2.89 hr; K13_WT  Date of collection: Oct 2008  Used in experiments for figure 3c, 3d, S7a |
| Biological sample (*Plasmodium falciparum*) | NHP4316 | This paper |  | Clinical isolate from the North western part of Thailand  AS-PCt_1/2_ = 1.43 hr; K13_WT  Date of collection: Oct 2008  Used in experiments for figures 1b, 1d, 1e, 2a, 2b, 2c, 2d, 2e, 2i, S3d, S4a, S4c, S5a, S5c, S6c, S6d |
| Biological sample (*Plasmodium falciparum*) | NHP1099 | This paper |  | Clinical isolate from the North western part of Thailand  AS-PCt_1/2_ = 2.91 hr; K13_WT  Date of collection: Oct 2008  Used in experiments for figure 3c, 3d, S7a |
| Biological sample (*Plasmodium falciparum*) | NHP1102 | This paper |  | Clinical isolate from the North western part of Thailand  AS-PCt_1/2_ = 2.85 hr; K13_WT  Date of collection: Oct 2008  Used in experiments for figure 3c, 3d, S7a |
| Biological sample (*Plasmodium falciparum*) | NHP1110 | This paper |  | Clinical isolate from the North western part of Thailand  AS-PCt_1/2_ = 2.23 hr; K13_WT  Date of collection: Oct 2008  Used in experiments for figure 3c, 3d, S7a |
| Biological sample (*Plasmodium falciparum*) | NHP1111 | This paper |  | Clinical isolate from the North western part of Thailand  AS-PCt_1/2_ = 1.75 hr; K13_WT  Date of collection: Oct 2008  Used in experiments for figure 3c, 3d, S7a |
| Biological sample (*Plasmodium falciparum*) | NHP1279 | This paper |  | Clinical isolate from the North western part of Thailand  AS-PCt_1/2_ = 3.08 hr; K13_WT  Date of collection: Jun 2010  Used in experiments for figure 3c, 3d S7a |
| Biological sample (*Plasmodium falciparum*) | NHP3127 | This paper |  | Clinical isolate from the North western part of Thailand  AS-PCt_1/2_ = 9.11 hr; K13_R561H  Date of collection: Jun 2010  Used in experiments for figures 1b, 2b, 2c, 2d, 2e, 2i, 3a, S3d, S6c, S6d |
| Biological sample (*Plasmodium falciparum*) | NHP1292 | This paper |  | Clinical isolate from the North western part of Thailand  AS-PCt_1/2_ = 3.67 hr; K13_S485N  Date of collection: Jul 2010  Used in experiments for figure 3c, 3d, S7a |
| Biological sample (*Plasmodium falciparum*) | NHP1301 | This paper |  | Clinical isolate from the North western part of Thailand  AS-PCt_1/2_ = N/A; K13_WT  Date of collection: Jun 2010  Used in experiments for figure 3c, 3d, S7a |
| Biological sample (*Plasmodium falciparum*) | NHP1332 | This paper |  | Clinical isolate from the North western part of Thailand  AS-PCt_1/2_ = 8.54 hr; K13_N458Y  Date of collection: Apr 2011  Used in experiments for figures 1b, 1c, 2b, 2c, 3a, S3d, S3f, S6c |
| Biological sample (*Plasmodium falciparum*) | NHP4703 | This paper |  | Clinical isolate from the North western part of Thailand  AS-PCt_1/2_ = 1.2 hr; K13_WT  Date of collection: Apr 2011  Used in experiments for figures 1b, 1c, 1d, 1e, 2b, 2c, 2d, 2e, 2i, S3d, S3e, S4a, S4c, S5a, S5c, S6c, S6d |
| Biological sample (*Plasmodium falciparum*) | NHP4722 | This paper |  | Clinical isolate from the North western part of Thailand  AS-PCt_1/2_ = 8.6 hr; K13_A675V  Date of collection: May 2011  Used in experiments for figures 1b, 1c, 1d, 1e, 2b, 2c, 2d, 2e, 2i, 3a, S3d, S3f, S4b, S4d, S5b, S6c, S6d |
| Biological sample (*Plasmodium falciparum*) | NHP3160 | This paper |  | Clinical isolate from the North western part of Thailand  AS-PCt_1/2_ = 8.59 hr; K13_N458Y  Date of collection: Jun 2011  Used in experiments for figures 1b, 1c, 2b, 2c, 3a, S3d, S3f, S6c |
| Biological sample (*Plasmodium falciparum*) | NHP1386 | This paper |  | Clinical isolate from the North western part of Thailand  AS-PCt_1/2_ = 1.67 hr; K13_WT  Date of collection: Jul 2011  Used in experiments for figures 1b, 1d, 1e, 2b, 2c, 2d, 2e, 2i, 3a, S3d, S4a, S4c, S5a, S5c, S6c, S6d |
| Biological sample (*Plasmodium falciparum*) | TH004-021 | This paper |  | Clinical isolate from the North western part of Thailand  AS-PCt_1/2_ = N/A; K13_A675V  Date of collection: Aug 2011  Used in experiments for figure 3c, 3d, S7a |
| Biological sample (*Plasmodium falciparum*) | TH004-022 | This paper |  | Clinical isolate from the North western part of Thailand  AS-PCt_1/2_ = N/A; K13_A675V  Date of collection: Sep 2011  Used in experiments for figures 3c, 3d, S7a, S7b |
| Biological sample (*Plasmodium falciparum*) | TH004-023 | This paper |  | Clinical isolate from the North western part of Thailand  AS-PCt_1/2_ = N/A; K13_A481V  Date of collection: Sep 2011  Used in experiments for figure 3c, 3d, S7a |
| Biological sample (*Plasmodium falciparum*) | TH004-024 | This paper |  | Clinical isolate from the North western part of Thailand  AS-PCt_1/2_ = N/A; K13_C580Y  Date of collection: Sep 2011  Used in experiments for figures 3c, 3d, S7a, S7b |
| Biological sample (*Plasmodium falciparum*) | TH004-084 | This paper |  | Clinical isolate from the North western part of Thailand  AS-PCt_1/2_ = N/A; K13_WT  Date of collection: Sep 2011  Used in experiments for figure 3c, 3d, S7a |
| Biological sample (*Plasmodium falciparum*) | TH004-086 | This paper |  | Clinical isolate from the North western part of Thailand  AS-PCt_1/2_ = N/A; K13_WT  Date of collection: Sep 2011  Used in experiments for figure 3c, 3d, S7a |
| Biological sample (*Plasmodium falciparum*) | NHP1393 | This paper |  | Clinical isolate from the North western part of Thailand  AS-PCt_1/2_ = 7.51 hr; K13_P441L  Date of collection: Nov 2011  Used in experiments for figure 1c, 3a, 3c, 3d, S3f, S7a |
| Biological sample (*Plasmodium falciparum*) | TH004-027 | This paper |  | Clinical isolate from the North western part of Thailand  AS-PCt_1/2_ = N/A; K13_C580Y  Date of collection: Nov 2011  Used in experiments for figures 3c, 3d, S7a, S7b |
| Biological sample (*Plasmodium falciparum*) | TH004-029 | This paper |  | Clinical isolate from the North western part of Thailand  AS-PCt_1/2_ = N/A; K13_A675V  Date of collection: Nov 2011  Used in experiments for figure 3c, 3d, S7a |
| Biological sample (*Plasmodium falciparum*) | TH004-031 | This paper |  | Clinical isolate from the North western part of Thailand  AS-PCt_1/2_ = N/A; K13_C580Y  Date of collection: Dec 2011  Used in experiments for figure 3c, 3d, S7a |
| Biological sample (*Plasmodium falciparum*) | TH004-090 | This paper |  | Clinical isolate from the North western part of Thailand  AS-PCt_1/2_ = N/A; K13_P441L  Date of collection: Dec 2011  Used in experiments for figures 3c, 3d, S7a, S7b |
| Biological sample (*Plasmodium falciparum*) | NHP4767 | This paper |  | Clinical isolate from the North western part of Thailand  AS-PCt_1/2_ = N/A; K13_C580Y  Date of collection: Dec 2011  Used in experiments for figure 3c, 3d, S7a |
| Biological sample (*Plasmodium falciparum*) | TH004-092 | This paper |  | Clinical isolate from the North western part of Thailand  AS-PCt_1/2_ = N/A; K13_WT  Date of collection: Dec 2011  Used in experiments for figure 3c, 3d, S7a |
| Biological sample (*Plasmodium falciparum*) | TH004-036 | This paper |  | Clinical isolate from the North western part of Thailand  AS-PCt_1/2_ = N/A; K13_R561H  Date of collection: Jan 2012  Used in experiments for figures 3c, 3d, S7a, S7b |
| Biological sample (*Plasmodium falciparum*) | TH004-100 | This paper |  | Clinical isolate from the North western part of Thailand  AS-PCt_1/2_ = N/A; K13_P441L  Date of collection: Feb 2012  Used in experiments for figures 3c, 3d, S7a, S7b |
| Biological sample (*Plasmodium falciparum*) | NHP1401 | This paper |  | Clinical isolate from the North western part of Thailand  AS-PCt_1/2_ = 9.18 hr; K13_C580Y  Date of collection: Feb 2012  Used in experiments for figures 1b, 1c, 1d, 1e, 2a, 2b, 2c, 2d, 2e, 2i, 3a, S3d, S3f, S4b, S4d, S5b, S6c, S6d |
| Biological sample (*Plasmodium falciparum*) | TH004-040 | This paper |  | Clinical isolate from the North western part of Thailand  AS-PCt_1/2_ = N/A; K13_A675V, C580Y, E252Q  Date of collection: Feb 2012  Used in experiments for figure 3c, 3d, S7a |
| Biological sample (*Plasmodium falciparum*) | TH004-042 | This paper |  | Clinical isolate from the North western part of Thailand  AS-PCt_1/2_ = N/A; K13_G538V  Date of collection: Feb 2012  Used in experiments for figure 3c, 3d, S7a |
| Biological sample (*Plasmodium falciparum*) | NHP1403 | This paper |  | Clinical isolate from the North western part of Thailand  AS-PCt_1/2_ = 7.7 hr; K13_A675V  Date of collection: Feb 2012  Used in experiments for figures 1b, 1c, 2b, 2c, 2d, 2e, 2i, 3a, S3d, S3f, S6c, S6d |
| Biological sample (*Plasmodium falciparum*) | TH004-097 | This paper |  | Clinical isolate from the North western part of Thailand  AS-PCt_1/2_ = N/A; K13_C580Y  Date of collection: Mar 2012  Used in experiments for figure 3c, 3d, S7a |
| Biological sample (*Plasmodium falciparum*) | TH004-098 | This paper |  | Clinical isolate from the North western part of Thailand  AS-PCt_1/2_ = N/A; K13_A675V  Date of collection: Mar 2012  Used in experiments for figure 3c, 3d, S7a |
| Biological sample (*Plasmodium falciparum*) | TH004-101 | This paper |  | Clinical isolate from the North western part of Thailand  AS-PCt_1/2_ = N/A; K13_A675V  Date of collection: Mar 2012  Used in experiments for figures 3c, 3d, S7a, S7b |
| Biological sample (*Plasmodium falciparum*) | NHP4777 | This paper |  | Clinical isolate from the North western part of Thailand  AS-PCt_1/2_ = 5.94 hr; K13_C580Y  Date of collection: Apr 2012  Used in experiments for figure 3c, 3d, S7a |
| Biological sample (*Plasmodium falciparum*) | NHP1085 | This paper |  | Clinical isolate from the North western part of Thailand  AS-PCt_1/2_ = 2.48 hr; K13_WT  Date of collection: May 2012  Used in experiments for figure 3c, 3d, S7a |
| Biological sample (*Plasmodium falciparum*) | TH004-065 | This paper |  | Clinical isolate from the North western part of Thailand  AS-PCt_1/2_ = N/A; K13_WT  Date of collection: May 2012  Used in experiments for figure 3c, 3d, S7a |
| Biological sample (*Plasmodium falciparum*) | TH004-070 | This paper |  | Clinical isolate from the North western part of Thailand  AS-PCt_1/2_ = N/A; K13_WT  Date of collection: May 2012  Used in experiments for figure 3c, 3d, S7a |
| Biological sample (*Plasmodium falciparum*) | NHP1445 | This paper |  | Clinical isolate from the North western part of Thailand  AS-PCt_1/2_ = 7.02 hr; K13_R561H  Date of collection: May 2012  Used in experiments for figure 1c, 3a, 3c, 3d, S3f, S7a |
| Biological sample (*Plasmodium falciparum*) | NHP1450 | This paper |  | Clinical isolate from the North western part of Thailand  AS-PCt_1/2_ = 8.87 hr; K13_N458Y  Date of collection: Jun 2012  Used in experiments for figures 1b, 1c, 1d, 1e, 2b, 2c, 2d, 2e, 2i, 3a, S3d, S3f, S4b, S4d, S5b, S5d, S6c, S6d |
| Biological sample (*Plasmodium falciparum*) | NHP1454 | This paper |  | Clinical isolate from the North western part of Thailand  AS-PCt_1/2_ = 7.98 hr; K13_R561H  Date of collection: Jun 2012  Used in experiments for figures 1b, 1c, 1d, 1e, 2b, 2c, 2d, 2e, 2i, S3d, S3f, S4b, S4d, S5b, S5d, S6c, S6d |
| Biological sample (*Plasmodium falciparum*) | NHP1455 | This paper |  | Clinical isolate from the North western part of Thailand  AS-PCt_1/2_ = 8.02 hr; K13_R561H  Date of collection: Jun 2012  Used in experiments for figures 1b, 1c, 1d, 1e, 2a, 2b, 2c, 2d, 2e, 2i, S3d, S3f, S4b, S4d, S5b, S5d, S6c, S6d |
| Biological sample (*Plasmodium falciparum*) | NHP1461 | This paper |  | Clinical isolate from the North western part of Thailand  AS-PCt_1/2_ = 9.21 hr; K13_P441L  Date of collection: Jun 2012  Used in experiments for figures 1b, 1c, 1d, 1e, 2b, 2c, 2d, 2e, 2i, 3a, S3d, S3f, S4d, S5b, S6c, S6d |
| Biological sample (*Plasmodium falciparum*) | TH004-117 | This paper |  | Clinical isolate from the North western part of Thailand  AS-PCt_1/2_ = N/A; K13_C580Y  Date of collection: Jun 2012  Used in experiments for figure 3c, 3d, S7a |
| Biological sample (*Plasmodium falciparum*) | TH004-120 | This paper |  | Clinical isolate from the North western part of Thailand  AS-PCt_1/2_ = N/A; K13_WT  Date of collection: Jun 2012  Used in experiments for figure 3c, 3d, S7a |
| Biological sample (*Plasmodium falciparum*) | TH004-113 | This paper |  | Clinical isolate from the North western part of Thailand  AS-PCt_1/2_ = N/A; K13_WT  Date of collection: Jun 2012  Used in experiments for figure 3c, 3d, S7a |
| Biological sample (*Plasmodium falciparum*) | NHP1481 | This paper |  | Clinical isolate from the North western part of Thailand  AS-PCt_1/2_ = 7.96 hr; K13_R561H  Date of collection: Jul 2012  Used in experiments for figures 1b, 1c, 1d, 1e, 2a, 2b, 2c, 2d, 2e, 2i, 3a, S6c, S3d, S3f, S4b, S4d, S5b, S5d, S6d |
| Biological sample (*Plasmodium falciparum*) | NHP4856 | This paper |  | Clinical isolate from the North western part of Thailand  AS-PCt_1/2_ = 4.96 hr; K13_C580Y  Date of collection: Dec 2012  Used in experiments for figure 3c, 3d, S7a |
| Biological sample (*Plasmodium falciparum*) | NHP1493 | This paper |  | Clinical isolate from the North western part of Thailand  AS-PCt_1/2_ = 6.57 hr; K13_P441L  Date of collection: Jan 2013  Used in experiments for figures 3c, 3d, S7a, S7b |
| Biological sample (*Plasmodium falciparum*) | NHP1499 | This paper |  | Clinical isolate from the North western part of Thailand  AS-PCt_1/2_ = 8.01 hr; K13_C580Y  Date of collection: Feb 2013  Used in experiments for figure 1c, 3c, 3d, S3f, S7a |
| Biological sample (*Plasmodium falciparum*) | NHP1494 | This paper |  | Clinical isolate from the North western part of Thailand  AS-PCt_1/2_ = 5.48 hr; K13_C580Y  Date of collection: Mar 2013  Used in experiments for figure 3c, 3d, S7a |
| Biological sample (*Plasmodium falciparum*) | NHP4870 | This paper |  | Clinical isolate from the North western part of Thailand  AS-PCt_1/2_ = 8.9 hr; K13_C580Y  Date of collection: Mar 2013  Used in experiments for figures 1b, 2b, 2c, 3a, S3d, S6c |
| Biological sample (*Plasmodium falciparum*) | NHP3215 | This paper |  | Clinical isolate from the North western part of Thailand  AS-PCt_1/2_ = 7.35 hr; K13_C580Y  Date of collection: Mar 2013  Used in experiments for figure 3c, 3d, S7a |
| Biological sample (*Plasmodium falciparum*) | NHP4873 | This paper |  | Clinical isolate from the North western part of Thailand  AS-PCt_1/2_ = 5.51 hr; K13_C580Y  Date of collection: Mar 2013  Used in experiments for figures 3c, 3d, S7a, S7b |
| Biological sample (*Plasmodium falciparum*) | NHP1501 | This paper |  | Clinical isolate from the North western part of Thailand  AS-PCt_1/2_ = 6.31 hr; K13_C580Y  Date of collection: Mar 2013  Used in experiments for figure 3c, 3d, S7a |
| Biological sample (*Plasmodium falciparum*) | NHP4885 | This paper |  | Clinical isolate from the North western part of Thailand  AS-PCt_1/2_ = 7.89 hr; K13_C580Y  Date of collection: Apr 2013  Used in experiments for figures 3c, 3d, S7a, S7b |
| Biological sample (*Plasmodium falciparum*) | NHP4898 | This paper |  | Clinical isolate from the North western part of Thailand  AS-PCt_1/2_ = 5.98 hr; K13_C580Y  Date of collection: May 2013  Used in experiments for figure 3c, 3d, S7a |
| Biological sample (*Plasmodium falciparum*) | NHP1503 | This paper |  | Clinical isolate from the North western part of Thailand  AS-PCt_1/2_ = 4.02 hr; K13_C580Y  Date of collection: May 2013  Used in experiments for figure 3c, 3d, S7a |
| Biological sample (*Plasmodium falciparum*) | NHP2203 | This paper |  | Clinical isolate from the North western part of Thailand  AS-PCt_1/2_ = 8.83 hr; K13_R575K  Date of collection: Jun 2013  Used in experiments for figures 1b, 1d, 1e, 2b, 2c, 2d, 2e, 2i, 3a, S3d, S4b, S4d, S5b, S6c, S6d |
| Biological sample (*Plasmodium falciparum*) | NHP4906 | This paper |  | Clinical isolate from the North western part of Thailand  AS-PCt_1/2_ = 6.22 hr; K13_C580Y  Date of collection: Jun 2013  Used in experiments for figures 3c, 3d, S7a, S7b |
| Biological sample (*Plasmodium falciparum*) | NHP4907 | This paper |  | Clinical isolate from the North western part of Thailand  AS-PCt_1/2_ = 6.20 hr; K13_C580Y  Date of collection: Jun 2013  Used in experiments for figures 3c, 3d, S7a, S7b |
| Biological sample (*Plasmodium falciparum*) | NHP1504 | This paper |  | Clinical isolate from the North western part of Thailand  AS-PCt_1/2_ = 6.35 hr; K13_R561H  Date of collection: Jun 2013  Used in experiments for figure 3c, 3d, S7a |
| Biological sample (*Plasmodium falciparum*) | NHP2204 | This paper |  | Clinical isolate from the North western part of Thailand  AS-PCt_1/2_ = 6.03 hr; K13_R561H  Date of collection: Jun 2013  Used in experiments for figure 3c, 3d, S7a |
| Biological sample (*Plasmodium falciparum*) | NHP4910 | This paper |  | Clinical isolate from the North western part of Thailand  AS-PCt_1/2_ = 6.69 hr; K13_C580Y  Date of collection: Jun 2013  Used in experiments for figure 3c, 3d, S7a |
| Biological sample (*Plasmodium falciparum*) | NHP4915 | This paper |  | Clinical isolate from the North western part of Thailand  AS-PCt_1/2_ = 6.96 hr; K13_C580Y  Date of collection: Jun 2013  Used in experiments for figure 3c, 3d, S7a |
| Biological sample (*Plasmodium falciparum*) | NHP3226 | This paper |  | Clinical isolate from the North western part of Thailand  AS-PCt_1/2_ = 7.44 hr; K13_C580Y  Date of collection: Jun 2013  Used in experiments for figure 3c, 3d, S7a |
| Biological sample (*Plasmodium falciparum*) | NHP4917 | This paper |  | Clinical isolate from the North western part of Thailand  AS-PCt_1/2_ = 5.49 hr; K13_C580Y  Date of collection: Jun 2013  Used in experiments for figures 3c, 3d, S7a, S7b |
| Biological sample (*Plasmodium falciparum*) | NHP1507 | This paper |  | Clinical isolate from the North western part of Thailand  AS-PCt_1/2_ = 9.24 hr; K13_R561H  Date of collection: Jun 2013  Used in experiments for figures 1b, 1d, 1e, 2b, 2c, 2d, 2e, 2i, 3a, S3d, S4b, S4d, S5b, S5d, S6c, S6d |
| Biological sample (*Plasmodium falciparum*) | NHP1508 | This paper |  | Clinical isolate from the North western part of Thailand  AS-PCt_1/2_ = 5.54 hr; K13_R561H  Date of collection: Jun 2013  Used in experiments for figure 3c, 3d, S7a |
| Biological sample (*Plasmodium falciparum*) | NHP2205 | This paper |  | Clinical isolate from the North western part of Thailand  AS-PCt_1/2_ = 6.62 hr; K13_R561H  Date of collection: Jun 2013  Used in experiments for figure 3c, 3d, S7a |
| Biological sample (*Plasmodium falciparum*) | NHP1511 | This paper |  | Clinical isolate from the North western part of Thailand  AS-PCt_1/2_ = 7.53 hr; K13_R561H  Date of collection: Jun 2013  Used in experiments for figure 3a, 3c, 3d, S7a |
| Biological sample (*Plasmodium falciparum*) | NHP1509 | This paper |  | Clinical isolate from the North western part of Thailand  AS-PCt_1/2_ = 6.30 hr; K13_R561H  Date of collection: Jun 2013  Used in experiments for figure 3c, 3d, S7a |
| Biological sample (*Plasmodium falciparum*) | NHP2206 | This paper |  | Clinical isolate from the North western part of Thailand  AS-PCt_1/2_ = 5.28 hr; K13_C580Y  Date of collection: Jul 2013  Used in experiments for figures 3c, 3d, S7a, S7b |
| Biological sample (*Plasmodium falciparum*) | NHP4930 | This paper |  | Clinical isolate from the North western part of Thailand  AS-PCt_1/2_ = 3.4 hr; K13_C580Y  Date of collection: Jul 2013  Used in experiments for figures 3c, 3d, S7a, S7b |
| Biological sample (*Plasmodium falciparum*) | NHP4922 | This paper |  | Clinical isolate from the North western part of Thailand  AS-PCt_1/2_ = 5.21 hr; K13_C580Y  Date of collection: Jul 2013  Used in experiments for figures 1d, 1e, 3a, 3c, 3d, S4b, S4d, S5b, S5d, S7a |
| Biological sample (*Plasmodium falciparum*) | NHP2207 | This paper |  | Clinical isolate from the North western part of Thailand  AS-PCt_1/2_ = 6.6 hr; K13_C580Y  Date of collection: Aug 2013  Used in experiments for figures 3c, 3d, S7a, S7b |
| Biological sample (*Plasmodium falciparum*) | NHP4939 | This paper |  | Clinical isolate from the North western part of Thailand  AS-PCt_1/2_ = 8.77 hr; K13_C580Y  Date of collection: Sep 2013  Used in experiments for figures 1b, 1d, 1e, 2b, 2c, 2d, 2e, 2i, 3a, S3d, S4b, S4d, S5b, S5d, S6c, S6d |
| Biological sample (*Plasmodium falciparum*) | NHP4931 | This paper |  | Clinical isolate from the North western part of Thailand  AS-PCt_1/2_ = 6.59 hr; K13_C580Y  Date of collection: Jul 2013  Used in experiments for figures 1d, 1e, 2d, 2e, 2i, 3a, S4b, S4d, S5b, S5d, S6d |
| Cell line (Homo sapiens) | THP-1 | ATCC | TIB-202^™^ | Monocytic cell line |
| Chemical compound, drug | Albumax II | Gibco™ | Cat # 11021045 | For parasite culture |
| Antibody | Anti-A (ABO1 ) murine monoclonal antibody | TransClone® Bio-Rad | Cat # 86328 | Blood group-typing  (slide method: 50 µl) |
| Antibody | Anti-B (ABO2) murine monoclonal antibody | TransClone® Bio-Rad | Cat # 86470 | Blood group-typing  (slide method: 50 µl) |
| Chemical compound, drug | Artesunate (AS) | Sigma-Aldrich® | Cat # A3731 |  |
| Other | BD Vacutainer™ with lithium heparin | ThermoFisher Scientific | Cat # 02-657-28 |  |
| Chemical compound, drug | Bovine Serum Albumin (BSA) | Sigma-Aldrich® | Cat # A2058 |  |
| Other | Cellulose acetate syringe filter, pore size 0.45 µm | Sartorius Minisart®, Sigma-Aldrich | Cat # 16555-K |  |
| Other | Centrifuge | Sorvall® | Legend® RT Plus |  |
| Software | CellSens Dimension | Olympus® |  | Microfluidics image  processing |
| Other | CF11 cellulose powder | Sigma-Aldrich® | Cat # 318094 |  |
| Software | Cytoscape version 3.6.1 | Cytoscape |  | For visualization of associations from bivariate analyses |
| Chemical compound, drug | Dihydroartemisinin (DHA) | Sigma-Aldrich® | Cat # D7439 |  |
| Chemical compound, drug | Dimethyl sulfoxide (DMSO) | Sigma-Aldrich® | Cat # D8418 |  |
| Other | Dual CCD digital camera for microscope | Olympus® | Model DP80 |  |
| Chemical compound, drug | Ethanol | Sigma-Aldrich® | Cat # E7023 |  |
| Other | Falcon® Cell Culture Flask T25, filter cap | VWR™ | Cat #29185298 |  |
| Chemical compound, drug | Fetal Bovine Serum (FBS) | Gibco™ | Cat # 10500 | For cell line culture |
| Other | Flat bottom plate, 96 well | NUNC™ | Cat #44240421 |  |
| Other | Giemsa | Merck | Cat # HX60416604 |  |
| Other | Glass coverslip 22x32mm | Mariendfeld | Cat # 0101112 |  |
| Other | Glass slide | Sail brand | Cat # 7105 |  |
| Software | GraphPad Prism9.0 | GraphPad |  | Statistical analysis |
| Other | LD columns | Miltenyi Biotec | Cat # 130-042-901 |  |
| Chemical compound, drug | Methanol | Merck | Cat#1.06009.2500 |  |
| Chemical compound, drug | Methyl-β-cyclodextrin (MBCD) | Sigma-Aldrich® | Cat # C4555 |  |
| Commercial assay or kit | MycoAlert™ Plus Mycoplasma detection kit | Lonza | Cat # LT07-705 | Routine screening of cultures for *Mycoplasma* contamination |
| Chemical compound, drug | Percoll® | Sigma-Aldrich® | Cat # P1644 |  |
| Other | QuadroMACS™ separator | Miltenyi Biotec | Cat # 130-090-976 |  |
| Chemical compound, drug | RPMI 1640 medium | HyClone™ | Cat # SH30255.01 |  |
| Software | R version 3.3.3 | R-statistics |  | For bivariate analyses |
| Other | Trypsin solution | Gibco™ | Cat # 25300054 |  |

***Supplementary Table 2*.** **Genotype combination of genes PF3D7_1343700 (coded for K13), PF3D7_0200300 and PF3D7_0223300 (both coded for parts of PfEMP1 exon 2) among the isolates recruited for experiments in Figure 3c, Supplementary Figures 7a and b.**

| K13 mutants  +  deletions in both  PF3D7_0200300  and  PF3D7_0223300 | K13 mutants  +  deletions in either  PF3D7_0200300  or  PF3D7_0223300 | K13 WT  +  deletions in both  PF3D7_0200300  and  PF3D7_0223300 | K13 WT  +  deletions in either  PF3D7_0200300  or  PF3D7_0223300 | K13 WT  +  no deletion in both  PF3D7_0200300  and  PF3D7_0223300 |
| --- | --- | --- | --- | --- |
| TH004-117 | NHP2205 | TH004-084 | NHP1059 | TH004-092 |
| NHP4910 | TH004-024 | TH004-070 | NHP1060 | TH004-065 |
| NHP4915 | TH004-027 | TH004-113 | NHP1279 | NHP1110 |
| NHP1499 | NHP2206 | TH004-120 | NHP1301 | NHP2019 |
| NHP1510 | NHP2207 | NHP1095 | TH004-086 |  |
| NHP3226 | NHP4930 | NHP1053 | NHP1085 |  |
| NHP4767 | NHP4885 | NHP1068 |  |  |
| NHP1503 | NHP4906 | NHP1069 |  |  |
| NHP4777 | NHP4873 | NHP1070 |  |  |
| NHP4922 | NHP4907 | NHP1088 |  |  |
| NHP4856 | NHP4917 | NHP1102 |  |  |
| NHP4898 | TH004-036 | NHP1099 |  |  |
| TH004-040 | TH004-022 | NHP1111 |  |  |
| NHP1508 | TH004-090 |  |  |  |
| NHP1509 | TH004-100 |  |  |  |
| NHP1393 | TH004-101 |  |  |  |
| NHP1504 | NHP1071 |  |  |  |
| TH004-031 | NHP1493 |  |  |  |
| TH004-097 |  |  |  |  |
| NHP1501 |  |  |  |  |
| NHP3215 |  |  |  |  |
| TH004-029 |  |  |  |  |
| TH004-042 |  |  |  |  |
| TH004-098 |  |  |  |  |
| TH004-023 |  |  |  |  |
| TH004-021 |  |  |  |  |
| NHP1039 |  |  |  |  |
| NHP1292 |  |  |  |  |
| NHP1445 |  |  |  |  |
| NHP1494 |  |  |  |  |
| NHP1511 |  |  |  |  |
| NHP2204 |  |  |  |  |

***Supplementary Table 3*. The 140 *P. falciparum* genes shortlisted for SNPs comparison.**

| **No.** | **Gene candidate** | **Gene ID** | **Chromosome** | **Description** | **# SNPs detected** |
| --- | --- | --- | --- | --- | --- |
| 1 | PFB0090c | PF3D7_0201800 | Pf3D7_02_v3 | 254688442;​258596856;​812614;​KAHsp40;​O96123;​PF02_0018;​PFB0090c knob associated heat shock protein 40 (KAHsp40) | 45 |
| 2 | PfEMP1 exon 2 | PF3D7_0223300 | Pf3D7_02_v3 | 124801577;​3845340;​812817;​O96294;​PF02_0221;​PFB1045w erythrocyte membrane protein 1+(PfEMP1), exon 2 | 3 |
| 3 | PfEMP1 exon 2 | PF3D7_0200300 | Pf3D7_02_v3 | 124799939;​3845073;​812599;​O96110;​PF02_0003;​PFB0020c erythrocyte membrane protein 1+(PfEMP1), exon 2 | 8 |
| 4 | PfEMP1 (VAR) | PF3D7_0100300 | Pf3D7_01_v3 | VAR-UPSA3 | 9 |
| 5 | PfEMP1 (VAR) | PF3D7_0100100 | Pf3D7_01_v3 | MAL1P4.01;PFA0005w;VAR-UPSB1 | 4 |
| 6 | PfEMP1 (VAR) | PF3D7_0115700 | Pf3D7_01_v3 | MAL1P4.29;VAR-UPSB1 | 1 |
| 7 | PfEMP1 (VAR) | PF3D7_0400100 | Pf3D7_04_v3 | 124505155;​23498127;​812552;​MAL4P1.1;​PFD0005w;​Q8I220;​VAR;​VAR-UPSB1 erythrocyte membrane protein 1, PfEMP1 (VAR) | 1 |
| 8 | PfEMP1 (VAR) | PF3D7_0400400 | Pf3D7_04_v3 | 124505159;​23498129;​812551;​MAL4P1.4;​PFD0020c;​Q8I218;​VAR;​VAR-UPSA1 erythrocyte membrane protein 1, PfEMP1 (VAR) | 19 |
| 9 | PfEMP1 (VAR) | PF3D7_0412400 | Pf3D7_04_v3 | 124505387;​6562758;​812583;​MAL4P2.56;​PFD0615c;​Q9U0G6;​VAR;​VAR-UPSC1 erythrocyte membrane protein 1, PfEMP1 (VAR) | 1 |
| 10 | PfEMP1 (VAR) | PF3D7_0412700 | Pf3D7_04_v3 | 124505391;​6562760;​812580;​MAL4P2.58;​PFD0625c;​Q9U0G5;​VAR;​VAR-UPSC1 erythrocyte membrane protein 1, PfEMP1 (VAR) | 3 |
| 11 | PfEMP1 (VAR) | PF3D7_0420700 | Pf3D7_04_v3 | 124505543;​23498272;​812476;​MAL4P1.194;​PFD0995c;​Q8IFQ6;​VAR;​VAR-UPSC1 erythrocyte membrane protein 1, PfEMP1 (VAR) | 93 |
| 12 | PfEMP1 (VAR) | PF3D7_0421300 | Pf3D7_04_v3 | 124505551;​23498276;​4794163;​MAL4P1.198;​PFD1015c;​Q8IFQ2;​VAR;​VAR-UPSC1 erythrocyte membrane protein 1, PfEMP1 (VAR) | 6 |
| 13 | PfEMP1 (VAR) | PF3D7_0425800 | Pf3D7_04_v3 | 124505639;​225632294;​23498320;​296005120;​812400;​MAL4P1.242;​MAL7P1.1;​PFD1235w;​VAR;​VAR-UPSA1 erythrocyte membrane protein 1, PfEMP1 (VAR) | 16 |
| 14 | PfEMP1 (VAR) | PF3D7_0600200 | Pf3D7_06_v3 | 2270.t00002;​3885990;​46362239;​86170340;​C6KSK8;​MAL6P1.316;​PFF0010w;​VAR;​VAR-UPSB2 erythrocyte membrane protein 1, PfEMP1 (VAR) | 11 |
| 15 | PfEMP1 (VAR) | PF3D7_0600400 | Pf3D7_06_v3 | 2270.t00005;​3885677;​46362241;​86170351;​C6KSL0;​MAL6P1.314;​PFF0020c;​VAR;​VAR-UPSA3 erythrocyte membrane protein 1, PfEMP1 (VAR) | 18 |
| 16 | PfEMP1 (VAR) | PF3D7_0617400 | Pf3D7_06_v3 | 2270.t00065;​3885794;​46361126;​86171174;​C6KT15;​MAL6P1.252;​PFF0845c;​VAR;​VAR-UPSC1 erythrocyte membrane protein 1, PfEMP1 (VAR) | 40 |
| 17 | PfEMP1 (VAR) | PF3D7_0632800 | Pf3D7_06_v3 | 2270.t00323;​3885832;​46361276;​86171928;​C6KTF9;​MAL6P1.1;​PFF1595c;​VAR;​VAR-UPSB1 erythrocyte membrane protein 1, PfEMP1 (VAR) | 3 |
| 18 | PfEMP1 (VAR) | PF3D7_0711700 | Pf3D7_07_v3 | 124511794;​23498798;​2654957;​PF07_0048;​Q8IBX3;​VAR;​VAR-UPSC1 erythrocyte membrane protein 1, PfEMP1 (VAR) | 6 |
| 19 | PfEMP1 (VAR) | PF3D7_0712000 | Pf3D7_07_v3 | 124511796;​23498799;​2654960;​PF07_0049;​Q8IBX2;​VAR;​VAR-UPSC1 erythrocyte membrane protein 1, PfEMP1 (VAR) | 21 |
| 20 | PfEMP1 (VAR) | PF3D7_0712300 | Pf3D7_07_v3 | 124511798;​23498800;​2654929;​MAL7P1.50;​Q8IBX1;​VAR;​VAR-UPSB1 erythrocyte membrane protein 1, PfEMP1 (VAR) | 84 |
| 21 | PfEMP1 (VAR) | PF3D7_0712600 | Pf3D7_07_v3 | 124511802;​23498802;​4794184;​PF07_0051;​Q8IBW9;​VAR;​VAR-UPSC1 erythrocyte membrane protein 1, PfEMP1 (VAR) | 72 |
| 22 | PfEMP1 (VAR) | PF3D7_0712900 | Pf3D7_07_v3 | 124511806;​23498804;​4794185;​MAL7P1.56;​Q8IBW7;​VAR;​VAR-UPSC2 erythrocyte membrane protein 1, PfEMP1 (VAR) | 0 |
| 23 | PfEMP1 (VAR) | PF3D7_0800100 | Pf3D7_08_v3 | 124512762;​23499283;​4794199;​MAL8P1.168;​PF08_0142;​Q8IAK1;​VAR;​VAR-UPSB1 erythrocyte membrane protein 1, PfEMP1 (VAR) | 6 |
| 24 | PfEMP1 (VAR) | PF3D7_0800200 | Pf3D7_08_v3 | 124512760;​23499282;​2655302;​MAL8P1.167;​PF08_0141;​Q8IAK2;​VAR;​VAR-UPSA3 erythrocyte membrane protein 1, PfEMP1 (VAR) | 3 |
| 25 | PfEMP1 (VAR) | PF3D7_0808600 | Pf3D7_08_v3 | 124512610;​23499207;​4794195;​MAL8P1.119;​PF08_0107;​Q8IAS3;​VAR;​VAR-UPSC1 erythrocyte membrane protein 1, PfEMP1 (VAR) | 10 |
| 26 | PfEMP1 (VAR) | PF3D7_0808700 | Pf3D7_08_v3 | 124512608;​23499206;​2655304;​MAL8P1.118;​PF08_0106;​Q8IAS4;​VAR;​VAR-UPSB7 erythrocyte membrane protein 1, PfEMP1 (VAR) | 8 |
| 27 | PfEMP1 (VAR) | PF3D7_0809100 | Pf3D7_08_v3 | 124512602;​23499203;​2655303;​PF08_0103;​Q8IAS7;​VAR;​VAR-UPSB6 erythrocyte membrane protein 1, PfEMP1 (VAR) | 27 |
| 28 | PfEMP1 (VAR) | PF3D7_0937600 | Pf3D7_09_v3 | 124507287;​23505270;​813643;​PFI1820w;​Q8I2E4;​VAR erythrocyte membrane protein 1, PfEMP1 (VAR) | 10 |
| 29 | PfEMP1 (VAR) | PF3D7_1100200 | Pf3D7_11_v3 | 124803377;​23495942;​810584;​PF11_0008;​Q8IIZ4;​VAR erythrocyte membrane protein 1, PfEMP1 (VAR) | 4 |
| 30 | PfEMP1 (VAR) | PF3D7_1200400 | Pf3D7_12_v3 | 124805343;​2277.t00004;​23496535;​811062;​MAL12P1.4;​PFL0020w;​Q8I640;​VAR;​VAR-UPSB5 erythrocyte membrane protein 1, PfEMP1 (VAR) | 32 |
| 31 | PfEMP1 (VAR2CSA) | PF3D7_1200600 | Pf3D7_12_v3 | 124805350;​2277.t00006;​23496537;​811060;​MAL12P1.6;​PFL0030c;​Q8I639;​VAR-UPSE;​VAR2CSA erythrocyte membrane protein 1, PfEMP1 (VAR2CSA) | 469 |
| 32 | PfEMP1 (VAR) | PF3D7_1219300 | Pf3D7_12_v3 | 124805987;​2277.t00187;​23496720;​811239;​MAL12P1.188;​PFL0935c;​Q8I5L5;​VAR;​VAR-UPSB1 erythrocyte membrane protein 1, PfEMP1 (VAR) | 64 |
| 33 | PfEMP1 (VAR) | PF3D7_1240300 | Pf3D7_12_v3 | 124806687;​2277.t00390;​23496924;​811442;​MAL12P1.388;​PFL1950w;​Q8I521;​VAR;​VAR-UPSB4 erythrocyte membrane protein 1, PfEMP1 (VAR) | 21 |
| 34 | PfEMP1 (VAR) | PF3D7_1240600 | Pf3D7_12_v3 | 124806691;​2277.t00392;​23496926;​811444;​MAL12P1.390;​PFL1960w;​Q8I519;​VAR;​VAR-UPSC1 erythrocyte membrane protein 1, PfEMP1 (VAR) | 30 |
| 35 | PfEMP1 (VAR) | PF3D7_1240900 | Pf3D7_12_v3 | 2277.t00394;​811446;​C6S3L1;​MAL12P1.392;​PFL1970w;​VAR erythrocyte membrane protein 1, PfEMP1 (VAR) | 35 |
| 36 | PfEMP1 (VAR) | PF3D7_1300300 | Pf3D7_13_v3 | 124512768;​23615157;​813648;​PF13_0003;​Q8IEU9;​VAR;​VAR-UPSA1 erythrocyte membrane protein 1, PfEMP1 (VAR) | 1 |
| 37 | PfHOP | PF3D7_1434300 | Pf3D7_14_v3 | 124809132;​23497393;​75016029;​811906;​HOP;​PF14_0324;​PfHOP;​Q8ILC1 Hsp70/Hsp90 organizing protein (HOP) | 121 |
| 38 | PfHSP40 subfamily A | PF3D7_1437900 | Pf3D7_14_v3 | 124809271;23497429;811941;PF14_0359; Q8IL88 HSP40, subfamily A, putative | 94 |
| 39 | PfHSP40, type II | PF3D7_0501100.2 | Pf3D7_05_v3 | HSP40;​MAL5P1.12;​PF3D7_0501100;​PFE0055c;​PFE55 heat shock protein 40, type II (HSP40) | 56 |
| 40 | PfHSP40, type II | PF3D7_0113700 | Pf3D7_01_v3 | MAL1P4.10; PFA0660 heat shock protein 40, type II | 87 |
| 41 | PfHSP70 | PF3D7_0818900 | Pf3D7_08_v3 | 124512406;​23499105;​2655254;​HSP70;​HSP70-1;​PF08_0054;​PfHsp70-1;​Q8IB24 heat shock protein 70 (HSP70) | 121 |
| 42 | PfHSP70-2 | PF3D7_0917900 | Pf3D7_09_v3 | 124506906;​23505079;​813455;​BIP;​GRP78;​HSP70-2;​PFI0875w;​Q8I2X4 heat shock protein 70 (HSP70-2) | 134 |
| 43 | PfHSP70-3 | PF3D7_1134000 | Pf3D7_11_v3 | 124804504;​23496277;​810898;​HSP70-3;​PF11_0351;​PfHsp70-3;​Q8II24;​mtHSP70 heat shock protein 70 (HSP70-3) | 134 |
| 44 | PfHSP70-x | PF3D7_0831700 | Pf3D7_08_v3 | 371941768;​9221911;​HSP70-x;​MAL7P1.228;​PfHSP70-x heat shock protein 70 (HSP70-x) | 64 |
| 45 | PfHSP 110 | PF3D7_1344200 | Pf3D7_13_v3 | 225632011;​296005494;​9221988;​C0H5H0;​HSP110;​HSP70-y;​MAL13P1.540 heat shock protein 110, putative (HSP110) | 344 |
| 46 | PfHSP110c | PF3D7_0708800 | Pf3D7_07_v3 | 124511738;​23498770;​2655069;​CG4;​HSP110c;​HSP70-z;​PF07_0033;​PfHSP70-z;​Q8IC01 heat shock protein 110 (HSP110c) | 139 |
| 47 | KAHRP | PF3D7_0202000 | Pf3D7_02_v3 | 124800689;​3845095;​812616;​KAHRP;​PF02_0020;​PFB0100c;​Q9TY99 knob-associated histidine-rich protein (KAHRP) | 93 |
| 48 | MC-2TM | PF3D7_0324100 | Pf3D7_03_v3 | 124505139;​4493995;​814556;​MAL3P7.47;​MC-2TM;​O97307;​PFC1080c;​PfMC-2TM Pfmc-2TM Maurer's cleft two transmembrane protein (MC-2TM) | 11 |
| 49 | MC-2TM | PF3D7_0713100 | Pf3D7_07_v3 | 124511810;​23498806;​2655005;​MAL7P1.58;​MC-2TM;​PfMC-2TM;​Q8IBW5 Pfmc-2TM Maurer's cleft two transmembrane protein (MC-2TM) | 44 |
| 50 | SBP1 | PF3D7_0501300 | Pf3D7_05_v3 | 124505949;​23504499;​812874;​MAL5P1.14;​PFE0065w;​Q8I487;​SBP1 skeleton-binding protein 1+(SBP1) | 59 |
| 51 | RIFIN | PF3D7_0100200 | Pf3D7_01_v3 | 124505647;​7670006;​MAL1P4.02;​PFA0010c;​RIF rifin,PIR protein (RIF) | 17 |
| 52 | RIFIN | PF3D7_0100600 | Pf3D7_01_v3 | 124505653;​7670010;​MAL1P4.06;​PFA0030c;​RIF rifin,PIR protein (RIF) | 16 |
| 53 | RIFIN | PF3D7_0114700 | Pf3D7_01_v3 | 124505911;​23476998;​MAL1P4.17;​PFA0710c;​RIF rifin,PIR protein (RIF) | 157 |
| 54 | STEVOR | PF3D7_0200400 | Pf3D7_02_v3 | 124799942;​3845075;​812600;​O96111;​PF02_0004;​PFB0025c | 43 |
| 55 | STEVOR | PF3D7_0201300 | Pf3D7_02_v3 | 254688441;​258596854;​812609;​O96118;​PF02_0013;​PFB0065w | 72 |
| 56 | STEVOR | PF3D7_0221400 | Pf3D7_02_v3 | 254688496;​258596945;​812795;​O96283;​PF02_0199;​PFB0955w | 112 |
| 57 | STEVOR | PF3D7_0222800 | Pf3D7_02_v3 | 254688498;​258596949;​812812;​O96291;​PF02_0216;​PFB1020w | 66 |
| 58 | STEVOR | PF3D7_0300400 | Pf3D7_03_v3 | 124504679;​4494022;​814324;​MAL3P8.4;​PFC0025c;​Q9U5L9 | 7 |
| 59 | STEVOR | PF3D7_0300900 | Pf3D7_03_v3 | 814327;​MAL3P8.8;​O97329;​PFC0045w stevor-like,PIR protein | 115 |
| 60 | STEVOR | PF3D7_0400800 | Pf3D7_04_v3 | 124505165;​23498132;​812539;​MAL4P1.7;​PFD0035c;​Q8I215 | 47 |
| 61 | PHISTb | PF3D7_0201600 | Pf3D7_02_v3 | 124800673;​23503341;​812612;​O96121;​PF02_0016;​PFB0080c; *Plasmodium* exported protein | 84 |
| 62 | PHISTc | PF3D7_0202100 | Pf3D7_02_v3 | 124800693;​23503343;​812617;​LSAP-2;​LSAP2;​O96125;​PF02_0021;​  unknown function, liver stage associated protein 2+(LSAP2) | 65 |
| 63 | PHISTc | PF3D7_0219700 | Pf3D7_02_v3 | 124801455;​23503435;​812780;​GEXP20;​O96272;​PF02_0184;​PFB0900c;​  PfGEXP20 *Plasmodium* exported protein (PHISTc), unknown function (GEXP20) | 102 |
| 64 | PHISTc | PF3D7_0219800 | Pf3D7_02_v3 | 254688492;​258596937;​  812781;​O96273;​PF02_0185;​PFB0905c *Plasmodium* exported protein (PHISTc), unknown function | 106 |
| 65 | PHISTb | PF3D7_0401800 | Pf3D7_04_v3 | 124505181;​23498140;​812531;​  ​MAL4P1.16;​PFD0080c;​PfD80;​Q8I207 Plasmodium exported protein (PHISTb), unknown function (PfD80) | 74 |
| 66 | PHISTa | PF3D7_0402000 | Pf3D7_04_v3 | 124505185;​23498142;​812529; ​MAL4P1.18; PFD0090c;​Q8I206 *Plasmodium* exported protein (PHISTa), unknown function | 118 |
| 67 | PHISTb | PF3D7_0402100 | Pf3D7_04_v3 | 124505187;​23498143;​812530;  ​ MAL4P1.19;​PFD0095c;​Q8I205 *Plasmodium* exported protein (PHISTb), unknown function | 212 |
| 68 | PHISTc | PF3D7_0424000 | Pf3D7_04_v3 | 812434;​C0H495;​MAL4P1.223;​ PFD1140w *Plasmodium* exported protein (PHISTc), unknown function | 174 |
| 69 | PHISTb | PF3D7_0424600 | Pf3D7_04_v3 | 124505615;​23498308;​812429;​ MAL4P1.229;​PFD1170c;​Q8IFM0 *Plasmodium* exported protein (PHISTb), unknown function | 72 |
| 70 | PHISTb | PF3D7_0424800 | Pf3D7_04_v3 | 124505619;​23498310;​812424;​  MAL4P1.231;​PFD1180w;​Q8IFL8 *Plasmodium* exported protein (PHISTb), unknown function | 125 |
| 71 | PHISTa | PF3D7_0424900 | Pf3D7_04_v3 | 124505621;​23498311;​812425;​  MAL4P1.232;​PFD1185w;​Q8IFL7 *Plasmodium* exported protein (PHISTa), unknown function | 59 |
| 72 | PHISTa | PF3D7_0425400 | Pf3D7_04_v3 | 124505633;​23498317;​812407;  ​ MAL4P1.238;​PFD1215w;​Q8IFL1 *Plasmodium* exported protein (PHISTa), unknown function | 115 |
| 73 | PHISTc | PF3D7_0532200 | Pf3D7_05_v3 | 124506545;​23504896;​813128;  ​MAL5P1.313;​PFE1595c;​Q8I3F2 *Plasmodium* exported protein (PHISTc), unknown function | 110 |
| 74 | PHISTb | PF3D7_0532300 | Pf3D7_05_v3 | 124506547;​23504897;​813129;  ​MAL5P1.314;​PFE1600w;​Q8I3F1 *Plasmodium* exported protein (PHISTb), unknown function | 102 |
| 75 | PHISTb | PF3D7_0532400 | Pf3D7_05_v3 | 124506547;​23504897;​813129;  ​MAL5P1.314;​PFE1600w;​Q8I3F1 *Plasmodium* exported protein (PHISTb), unknown function | 98 |
| 76 | PHISTb | PF3D7_0601500 | Pf3D7_06_v3 | 225631715;​2270.t00299;​296004618;​3885746;  ​C6KSL7;​MAL6P1.19;​PFF0075c *Plasmodium* exported protein (PHISTb), unknown function | 2 |
| 77 | PHISTb | PF3D7_0731300 | Pf3D7_07_v3 | 124512146;​23498974;​2654996;  ​MAL7P1.174;​PfG174;​Q8IBE9 *Plasmodium* exported protein (PHISTb), unknown function (PfG174) | 58 |
| 78 | PHISTc | PF3D7_0801000 | Pf3D7_08_v3 | 124512744;​23499274;​2655336;​  PF08_0137;​Q8IAK9 *Plasmodium* exported protein (PHISTc), unknown function | 234 |
| 79 | PHISTc | PF3D7_0830600 | Pf3D7_08_v3 | 124512184;​23498994;​2655380;  ​MAL8P1.4;​Q8IBD2 *Plasmodium* exported protein (PHISTc), unknown function | 197 |
| 80 | PHISTb | PF3D7_0831000 | Pf3D7_08_v3 | 124512176;​23498990;​2655365;  ​GEXP09;​MAL8P1.2;​PfGEXP09;​Q8IBD6 *Plasmodium* exported protein (PHISTb), unknown function (GEXP09) | 188 |
| 81 | PHIST | PF3D7_0831500 | Pf3D7_08_v3 | *Plasmodium* exported protein (PHIST) ; unknown function | 93 |
| 82 | PHISTa-like | PF3D7_0832200.1 | Pf3D7_08_v3 | *Plasmodium* exported protein (PHISTa-like) ; unknown function | 2 |
| 83 | PHISTa-like | PF3D7_0832200.2 | Pf3D7_08_v3 | 225632124;​296004776;​MAL7P1.225;​PF3D7_0832200 *Plasmodium* exported protein (PHISTa-like), unknown function | 133 |
| 84 | PHISTa-like | PF3D7_0832300 | Pf3D7_08_v3 | 225632123;​296004774;​9221887; C0H4J2;​MAL7P1.224 *Plasmodium* exported protein (PHISTa-like), unknown function | 125 |
| 85 | PHISTc | PF3D7_0936600 | Pf3D7_09_v3 | 124507267;​23505260;​813634;​  GEXP05;​PFI1770w;​PfGEXP05;​Q8I2F4 *Plasmodium* exported protein (PHISTc), unknown function (GEXP05) | 90 |
| 86 | PHISTc | PF3D7_0936800 | Pf3D7_09_v3 | 124507271;​23505262;​813636;  PFI1780w;​Q8I2F2 *Plasmodium* exported protein (PHISTc), unknown function | 108 |
| 87 | PHISTb | PF3D7_0937000 | Pf3D7_09_v3 | 225631878;​296005323;​813638;  ​C0H593;​PFI1790w *Plasmodium* exported protein (PHISTb), unknown function | 151 |
| 88 | PHISTa | PF3D7_1001300 | Pf3D7_10_v3 | 254922370;​258596961;​810175;​ PF10_0017;​ Q8IK23 *Plasmodium* exported protein (PHISTa), unknown function | 95 |
| 89 | PHISTc | PF3D7_1001700 | Pf3D7_10_v3 | 124801919;​23494884; 810179; ​PF10_0021;​Q8IK19 *Plasmodium* exported protein (PHISTc), unknown function | 63 |
| 90 | PHISTc | PF3D7_1001800 | Pf3D7_10_v3 | 254922371;​258596963;​810180;​ PF10_0022;​ Q8IK18 *Plasmodium* exported protein (PHISTc), unknown function | 68 |
| 91 | PHISTc | PF3D7_1016500 | Pf3D7_10_v3 | 254922417;​258597042;​810319;​ PF10_0161; ​Q8IJN2 *Plasmodium* exported protein (PHISTc), unknown function | 222 |
| 92 | PHISTc | PF3D7_1016600 | Pf3D7_10_v3 | 254922418;​258549061;​8444980;​ C6S3C7; PF10_0161;​ PF10_0161a *Plasmodium* exported protein (PHISTc), unknown function | 94 |
| 93 | PHISTc | PF3D7_1016700 | Pf3D7_10_v3 | 254922419;​258597044;​810320;​ PF10_0162;​ Q8IJN1 *Plasmodium* exported protein (PHISTc), unknown function | 338 |
| 94 | PHISTc | PF3D7_1016800 | Pf3D7_10_v3 | 124802357;​23495028; ​810321; ​PF10_0163;​Q8IJN0 *Plasmodium* exported protein (PHISTc), unknown function | 58 |
| 95 | RESA-like protein with PHIST & DnaJ domains | PF3D7_1038800 | Pf3D7_10_v3 | 124803007;​23495246;​810535;​PF10_0378;​Q8IJ23 | 329 |
| 96 | PHISTb | PF3D7_1102500 | Pf3D7_11_v3 | 124803430;​23495963;​810590; GEXP02; ​PF11_0037;​PfGEXP02;​Q8IIX5, unknown function (GEXP02) | 208 |
| 97 | PHISTc | PF3D7_1148700 | Pf3D7_11_v3 | 124804948;​23496416;​811035; GEXP12;​ PF11_0503;​PfGEXP12;​ Q8IHN7 *Plasmodium* exported protein (PHISTc), unknown function (GEXP12) | 130 |
| 98 | PHISTa | PF3D7_1149700 | Pf3D7_11_v3 | 124804985;​23496427;​811046; PF11_0514;​ Q8IHM6 *Plasmodium* exported protein (PHISTa), unknown function | 16 |
| 99 | PHISTc | PF3D7_1200900 | Pf3D7_12_v3 | 124805359;​2277.t00009;​23496540;​811057; MAL12P1.9;​ PFL0045c;​Q8I636 *Plasmodium* exported protein (PHISTc), unknown function | 83 |
| 100 | PHISTb | PF3D7_1201000 | Pf3D7_12_v3 | 124805363;​2277.t00010;​23496541;​811056;  ​MAL12P1.10;​PFL0050c;​Q8I635 *Plasmodium* exported protein (PHISTb), unknown function | 126 |
| 101 | RESA-like protein with PHIST & DnaJ domains | PF3D7_1201100 | Pf3D7_12_v3 | 124805367;​2277.t00011;​23496542;​811055;  ​MAL12P1.11;​PFL0055c;​Q8I634 RESA-like protein with PHIST and DnaJ domains | 277 |
| 102 | PHISTa-like | PF3D7_1201200 | Pf3D7_12_v3 | 124805371;​2277.t00012;​23496543;​811054;  ​MAL12P1.12;​PFL0060w;​Q8I633 *Plasmodium* exported protein (PHISTa-like), unknown function | 56 |
| 103 | PHISTb | PF3D7_1252700 | Pf3D7_12_v3 | 124807103;​2277.t00505;  ​23497039;​811557;​MAL12P1.502;​PFL2535w;​Q8I4Q9 *Plasmodium* exported protein (PHISTb), unknown function | 106 |
| 104 | PHISTb | PF3D7_1252800 | Pf3D7_12_v3 | 124807106;​2277.t00506; 23497040; ​811558;​MAL12P1.503;​PFL2540w;​Q8I4Q8 *Plasmodium* exported protein (PHISTb), unknown function | 139 |
| 105 | PHISTa | PF3D7_1301500 | Pf3D7_13_v3 | 124513004;​23615275;​813948;​ MAL13P1.59;​Q8IEJ1 *Plasmodium* exported protein (PHISTa), unknown function | 107 |
| 106 | PHISTa | PF3D7_1372000 | Pf3D7_13_v3 | 225632074;​296005620;​ 9221997; ​C0H5M7;​MAL13P1.470 *Plasmodium* exported protein (PHISTa), unknown function | 215 |
| 107 | PHISTb | PF3D7_1372100 | Pf3D7_13_v3 | 225632075;​296005622;​9221998; C0H5M8;​GEXP04;​MAL13P1.475;​PfGEXP04 *Plasmodium* exported protein (PHISTb), unknown function (GEXP04) | 323 |
| 108 | PHISTb | PF3D7_1401600 | Pf3D7_14_v3 | 255528744;​258597612;​811593; PF14_0018;​Q8IM73 *Plasmodium* exported protein (PHISTb), unknown function | 206 |
| 109 | PHISTb | PF3D7_1476200 | Pf3D7_14_v3 | 255528949;​258597965;​812312;​ PF14_0730;​PF14_0731;​Q8IK76 *Plasmodium* exported protein (PHISTb), unknown function | 165 |
| 110 | PHISTb | PF3D7_1476300 | Pf3D7_14_v3 | 124810519;​23497808;​812314;​  PF14_0732;​Q8IK74 *Plasmodium* exported protein (PHISTb), unknown function | 169 |
| 111 | PHISTb | PF3D7_1477500 | Pf3D7_14_v3 | 255528955;​258597975;​ 812328;​PF14_0746;​Q8IK60 *Plasmodium* exported protein (PHISTb), unknown function | 98 |
| 112 | PHISTa | PF3D7_1477700 | Pf3D7_14_v3 | 255528956;​258597977;​812330;​ PF14_0748;​Pfg14-748;​Pfg14.748;​Q8IK58 *Plasmodium* exported protein (PHISTa), unknown function (Pfg14-748) | 99 |
| 113 | PHISTa | PF3D7_1478000 | Pf3D7_14_v3 | 124810562;​23497827;​812333;​ GEXP17;​PF14_0752;​PfGEXP17;​Q8IK55 *Plasmodium* exported protein (PHISTa), unknown function (GEXP17)) | 113 |
| 114 | PHISTa | PF3D7_1479200 | Pf3D7_14_v3 | 124810585;​23497838;​812344; PF14_0763;​Q8IK45 *Plasmodium* exported protein (PHISTa), unknown function | 14 |
| 115 | PTEX88 | PF3D7_1105600 | Pf3D7_11_v3 | 254832595;​258597205;​810619;  PF11_0067;​PTEX88;​Q8IIU7 translocon component PTEX88 | 331 |
| 116 | PTEX150 | PF3D7_1436300 | Pf3D7_14_v3 | 124809214;​23497413;​811926;  ​PF14_0344;​PTEX150;​Pf112;​Q8ILA1 translocon component PTEX150 (PTEX150) | 338 |
| 117 | PI3K | PF3D7_0515300 | Pf3D7_05_v3 | 124506225;​23504638;​812967;​  MAL5P1.153;​PFE0765w;​PI3K;​PfPI3K;​Q8I3V5;​VPS34 phosphatidylinositol 3-kinase (PI3K) | 553 |
| 118 | PfUBP | PF3D7_0403500 | Pf3D7_04_v3 | 124505209; 23498154; 812487; MAL4P1.33; PFD0165w; Q8I1Z5, ubiquitin specific protease, putative | 96 |
| 119 | PfUBP | PF3D7_0904600 | Pf3D7_09_v3 | 124506645;?23504947;?813325;?PFI0225w; Q8I3A3, ubiquitin specific protease, putative | 695 |
| 120 | *pf*ap2mu | PF3D7_1218300 | Pf3D7_12_v3 | 124805951;2277.t00177;23496709;811229;MAL12P1.177;  PFL0885w; Q8I5M5 AP-2 complex subunit mu, putative; pfap2mu | 139 |
| 121 | *Pf*coronin | PF3D7_1251200 | Pf3D7_12_v3 | 124807051;​2277.t00492;​23497026;​811544;​MAL12P1.489;​PFL2460w;​Q8I4S2 coronin | 162 |
| 122 | *Pf*spp | PF3D7_1457000 | Pf3D7_14_v3 | 223712821;​223712823;​223712825;​223712827;​223712829;​255528896;​258597872;​812125;​PF14_0543;​PfSPP;​Q8IKQ9;​SPP signal peptide peptidase (SPP) | 60 |
| 123 | *Pf*trx1 | PF3D7_1457200 | Pf3D7_14_v3 | 124809933;​23497618;​75009811;​7799189;​812127;​PF14_0545;​Q7KQL8;​Q9NFK9;​TRX1 thioredoxin 1+(TRX1) | 24 |
| 124 | *Pf*mdr1 | PF3D7_0523000 | Pf3D7_05_v3 | 124506379;​126936;​160399;​23504716;​4261570;​813045;​9936;​MAL5P1.230;​MDR1;​PFE1150w;​PfMDR1;​Pgh1;​Q7K6A5 multidrug resistance protein (MDR1) | 200 |
| 125 | *Pf*mdr2 | PF3D7_1447900 | Pf3D7_14_v3 | 124809620;​23497526;​812037;​MDR2;​PF14_0455;​PfMDR2;​Q8IKZ6 multidrug resistance protein 2+(heavy metal transport family) (MDR2) | 277 |
| 126 | *Pf*CRT | PF3D7_0709000 | PF3D7_07_v3 | 124511742;​23498772;​2655199;​7331101;​7331105;​74815295;​74824947;​CRT;​MAL7P1.27;​Q8IBZ9 chloroquine resistance transporter (CRT) | 89 |
| 127 | *Pf*ARPS10 | PF3D7_1460900.1 | Pf3D7_14_v3 | 124810084;​23497654;​PF14_0581;​PF3D7_1460900 apicoplast ribosomal protein S10 precursor, putative | 64 |
| 128 | Ferredoxin (fd) | PF3D7_1318100 | Pf3D7_13_v3 | 124513122;​23615334;​813986;​ MAL13P1.95;​Q8IED5 ferredoxin (fd), putative | 47 |
| 129 | Conserved *Plasmodium* protein | PF3D7_1451200 | Pf3D7_14_v3 | 124809716;​23497559;​812069;​PF14_0487;​Q8IKW4 conserved *Plasmodium* protein, unknown function | 826 |
| 130 | Phosphoinositide-binding protein | PF3D7_0720700 | Pf3D7_07_v3 | 225632173;​2654930;​296004875;​MAL7P1.108;​MAL7P1.109;​Q8IBP4 phosphoinositide-binding protein, putative | 660 |
| 131 | Conserved *Plasmodium* protein | PF3D7_1322700 | Pf3D7_13_v3 | 225631969;​296005409;​813677;​C0H5D1;​MAL13P1.127, conserved *Plasmodium* protein, unknown function | 76 |
| 132 | *Pf*NIF4 | PF3D7_1012700 | Pf3D7_10_v3 | 124802229;​23494988;​810282;​NIF4;​PF10_0124;​Q8IJR8 NLI interacting factor-like phosphatase, putative (NIF4) | 594 |
| 133 | DNA repair protein | PF3D7_0710400 | Pf3D7_07_v3 | 2655205;​MAL7P1.32;​Q8IBY6 DNA repair protein, putative | 113 |
| 134 | IMP1-like protein | PF3D7_1006000 | Pf3D7_10_v3 | 124802046;​23494925;​810219;​PF10_0061;​Q8IJY0 conserved *Plasmodium* protein, unknown function | 149 |
| 135 | RUVB2 | PF3D7_1106000 | Pf3D7_11_v3 | 124803531;​23495998;​810623;​PF11_0071;​Q8IIU3;​RUVB2 RuvB-like helicase 2+(RUVB2) | 108 |
| 136 | ERCC4 | PF3D7_1368800 | Pf3D7_13_v3 | 124514084;​23615815;​813914;​ERCC4;​MAL13P1.346;​Q8ID22 DNA repair endonuclease, putative (ERCC4) | 749 |
| 137 | ATP-dependent DNA helicase | PF3D7_1429900 | Pf3D7_14_v3 | 124808958;​23497346;​811860;​PF14_0278;​Q8ILG5 ATP-dependent DNA helicase, putative | 375 |
| 138 | Q8IKS5 conserved *Plasmodium* protein | PF3D7_1455300 | Pf3D7_14_v3 | 124809850;​23497599;​812109;​PF14_0527;​Q8IKS5 conserved *Plasmodium* protein, unknown function | 248 |
| 139 | MAHRP1 | PF3D7_1370300 | Pf3D7_13_v3 | 225632066;​296005604;​34305469;​9222008;​C0H5L9;​MAHRP1;​MAL13P1.413 membrane associated histidine-rich protein (MAHRP1) | 49 |
| 140 | MAHRP2 | PF3D7_1353200 | Pf3D7_13_v3 | 124513776;​23615661;​814241;​MAHRP2;​PF13_0276;​Q8IDG8 membrane associated histidine-rich protein (MAHRP2) | 44 |

***Supplementary Table 4*. Sequenced Thai *P. falciparum* isolates retrieved from MalariaGEN database.** The status of their K13, along with status in genes PF3D7_0200300 and PF3D7_0223300 are shown. *N/A: information not available.

| **No.** | **Sample ID** | **Year** | **SMRU codes** | **K13 status**  **(PF3D7_1343700; chromosome 13)** | **PfEMP1 exon 2**  **(PF3D7_0200300; chromosome 2)** | **PfEMP1 exon 2 (PF3D7_0223300; chromosome 2)** |
| --- | --- | --- | --- | --- | --- | --- |
| 1 | PD1394-C | 2003 | OHP00291 | E252Q | ATTGCTCG | --- |
| 2 | PD0940-C | 2007 | NHP4029 | R515K | ATTGCTCG | --- |
| 3 | PD0945-C | 2007 | NHP4038 | A675V | ATTGCTCG | --- |
| 4 | PD0997-C | 2008 | NHP4119 | E252Q | ATTGCTCG | --- |
| 5 | PD0720-C | 2008 | NHP1023 | P527H | ATTGCTCG | --- |
| 6 | PD0725-C | 2008 | NHP1032 | P527H | ATTGCTCG | --- |
| 7 | PD0744-C | 2008 | NHP1063 | E252Q | ATTGCTCG | GGC |
| 8 | PD0746-C | 2008 | NHP1065 | A675V | ATTGCTCG | --- |
| 9 | PD0751-C | 2008 | NHP1071 | P527H | ATTGCTCG | --- |
| 10 | PD0867-C | 2008 | NHP2065 | A675V | ATTGCTCG | GGC |
| 11 | PD1030-C | 2008 | NHP4180 | E252Q | ATTGCTCG | --- |
| 12 | PD1036-C | 2008 | NHP4192 | A675V | ATTGCTCG | --- |
| 13 | PD1151-C | 2010 | NHP4676 | R561H | ATTGCTCG | --- |
| 14 | PD0477-C | 2011 | TH004-024 | C580Y | ATTGCTCG | --- |
| 15 | PD0479-C | 2011 | TH004-027 | C580Y | ATTGCTCG | --- |
| 16 | PD0552-C | 2011 | TH004-090 | P441L | ATTGCTCG | --- |
| 17 | PD0492-C | 2011 | TH004-088 | C580Y | ATTGCTCG | --- |
| 18 | PD0513-C | 2011 | TH004-022 | A675V | ATTGCTCG | --- |
| 19 | PD0557-C | 2012 | TH004-101 | A675V | ATTGCTCG | --- |
| 20 | PD0563-C | 2012 | TH004-109 | P441L | ATTGCTCG | --- |
| 21 | PD0584-C | 2012 | N/A | G449A | ATTGCTCG | --- |
| 22 | PD0587-C | 2012 | N/A | G449A | ATTGCTCG | --- |
| 23 | PD0632-C | 2012 | TH004-033 | E252Q | ATTGCTCG | --- |
| 24 | PD0637-C | 2012 | TH004-075 | N537I | ATTGCTCG | GGC |
| 25 | PD0481-C | 2012 | TH004-036 | R561H | ATTGCTCG | --- |
| 26 | PD0521-C | 2012 | TH004-048 | F446I | ATTGCTCG | --- |
| 27 | PD0523-C | 2012 | TH004-050 | C580Y, F446I | ATTGCTCG | --- |
| 28 | PD0534-C | 2012 | TH004-063 | C580Y, G538V | ATTGCTCG | --- |
| 29 | PD0537-C | 2012 | TH004-066 | C580Y, G538V | ATTGCTCG | --- |
| 30 | PD0548-C | 2012 | TH004-079 | A675V | ATTGCTCG | --- |
| 31 | PD0802-C | 2012 | NHP1486 | R575K | ATTGCTCG | --- |
| 32 | PD0924-C | 2012 | NHP3212 | A675V | ATTGCTCG | --- |
| 33 | PD0556-C | 2012 | TH004-100 | P441L | ATTGCTCG | --- |
| 34 | PD1166-C | 2012 | NHP4844 | E252Q | ATTGCTCG | --- |
| 35 | PD1168-C | 2012 | NHP4846 | E252Q | ATTGCTCG | --- |
| 36 | PD1169-C | 2012 | NHP4847 | E252Q | ATTGCTCG | --- |
| 37 | PD1170-C | 2012 | NHP4848 | N537I | ATTGCTCG | GGC |
| 38 | PD1180-C | 2012 | NHP4858 | E252Q | ATTGCTCG | --- |
| 39 | PD0518-C | 2012 | TH004-044 | G538V | ATTGCTCG | --- |
| 40 | PD0488-C | 2012 | TH004-043 | E252Q | ATTGCTCG | --- |
| 41 | PD0516-C | 2012 | TH004-034 | C580Y | ATTGCTCG | --- |
| 42 | PD0517-C | 2012 | TH004-035 | C580Y, E252Q | ATTGCTCG | --- |
| 43 | PD0879-C | 2013 | NHP2207 | C580Y | ATTGCTCG | --- |
| 44 | PD0913-C | 2013 | NHP3058 | R515K | ATTGCTCG | --- |
| 45 | PD0670-C | 2013 | N/A | R539T | ATTGCTCG | --- |
| 46 | PD0808-C | 2013 | NHP1493 | P441L | ATTGCTCG | --- |
| 47 | PD0810-C | 2013 | NHP1495 | P441L | ATTGCTCG | --- |
| 48 | PD0820-C | 2013 | NHP1505 | P441L | ATTGCTCG | --- |
| 49 | PD0829-C | 2013 | NHP1514 | A481V | ATTGCTCG | --- |
| 50 | PD0830-C | 2013 | NHP1515 | P441L | ATTGCTCG | --- |
| 51 | PD0878-C | 2013 | NHP2206 | C580Y | ATTGCTCG | --- |
| 52 | PD1193-C | 2013 | NHP4873 | A675V, C580Y | ATTGCTCG | --- |
| 53 | PD1196-C | 2013 | NHP4877 | A675V, C580Y, E252Q | ATTGCTCG | --- |
| 54 | PD1197-C | 2013 | NHP4878 | A675V | ATTGCTCG | --- |
| 55 | PD1204-C | 2013 | NHP4885 | C580Y | ATTGCTCG | --- |
| 56 | PD1209-C | 2013 | NHP4893 | N537I | ATTGCTCG | GGC |
| 57 | PD1212-C | 2013 | NHP4896 | C580Y | ATTGCTCG | --- |
| 58 | PD1213-C | 2013 | NHP4897 | C580Y, G449A | ATTGCTCG | --- |
| 59 | PD1215-C | 2013 | NHP4899 | C580Y | ATTGCTCG | --- |
| 60 | PD1222-C | 2013 | NHP4906 | C580Y | ATTGCTCG | --- |
| 61 | PD1223-C | 2013 | NHP4907 | C580Y | ATTGCTCG | --- |
| 62 | PD1232-C | 2013 | NHP4917 | C580Y | ATTGCTCG | --- |
| 63 | PD1239-C | 2013 | NHP4925 | C580Y | ATTGCTCG | --- |
| 64 | PD1244-C | 2013 | NHP4930 | C580Y | ATTGCTCG | --- |
| 65 | PD1246-C | 2013 | NHP4932 | C580Y, K479I | ATTGCTCG | --- |
| 66 | PD1249-C | 2013 | NHP4936 | K479I | ATTGCTCG | --- |
| 67 | PD1191-C | 2013 | NHP4870 | C580Y | ATTGCTCG | --- |
| 68 | PD0951-C | 2007 | NHP4046 | E252Q | A------- | --- |
| 69 | PD0775-C | 2008 | NHP1103 | E252Q | ATTG---- | GGC |
| 70 | PD1041-C | 2008 | NHP4199 | G538V, K438N | ATTG---- | GGC |
| 71 | PD1046-C | 2008 | NHP4206 | E252Q | ATTG---- | --- |
| 72 | PD1085-C | 2008 | NHP4264 | E252Q | ATTG---- | --- |
| 73 | PD1106-C | 2008 | NHP4290 | E252Q | ATTG---- | --- |
| 74 | PD1108-C | 2008 | NHP4294 | E252Q | ATTG---- | --- |
| 75 | PD0731-C | 2008 | NHP1039 | P527H | ----CTCG | --- |
| 76 | PD0870-C | 2008 | NHP2198 | P441L | A---CTCG | --- |
| 77 | PD0793-C | 2010 | NHP1292 | S485N | ----CTCG | --- |
| 78 | PD0922-C | 2010 | NHP3125 | R561H, E252Q | ----CTCG | --- |
| 79 | PD0921-C | 2010 | NHP3118 | E252Q | ---GCTCG | --- |
| 80 | PD0505-C | 2011 | N/A | C580Y | ATTG---- | GGC |
| 81 | PD0508-C | 2011 | N/A | C580Y | ATTG---- | GGC |
| 82 | PD0499-C | 2011 | N/A | C580Y | ATTG---- | --- |
| 83 | PD0504-C | 2011 | N/A | C580Y | ATTG---- | GGC |
| 84 | PD0798-C | 2011 | NHP1393 | P441L | --TGCTCG | --- |
| 85 | PD1153-C | 2011 | NHP4696 | E252Q | ATTG---- | --- |
| 86 | PD1164-C | 2011 | NHP4767 | C580Y | ATTG---- | --- |
| 87 | PD0460-C | 2011 | NHP4062 | E252Q | AT------ | --- |
| 88 | PD0473-C | 2011 | TH004-018 | C580Y | ----CTCG | --- |
| 89 | PD0478-C | 2011 | TH004-025 | P441L | AT------ | GGC |
| 90 | PD0491-C | 2011 | TH004-087 | P441L | ----CTCG | GGC |
| 91 | PD0503-C | 2011 | N/A | P574L | AT------ | --- |
| 92 | PD0630-C | 2011 | TH004-030 | E252Q | ----CTCG | --- |
| 93 | PD0519-C | 2012 | TH004-045 | C580Y | A------- | --- |
| 94 | PD0520-C | 2012 | TH004-047 | C580Y | ----CTCG | --- |
| 95 | PD0524-C | 2012 | TH004-052 | E252Q | A------- | --- |
| 96 | PD0529-C | 2012 | TH004-057 | A675V | ----CTCG | --- |
| 97 | PD0544-C | 2012 | TH004-074 | E252Q | A------- | GGC |
| 98 | PD0545-C | 2012 | TH004-076 | C580Y | A------- | --- |
| 99 | PD0549-C | 2012 | TH004-080 | G538V | A------- | --- |
| 100 | PD0566-C | 2012 | TH004-112 | P441L | ----CTCG | GGC |
| 101 | PD0577-Cx | 2012 | N/A | C580Y | A------- | --- |
| 102 | PD0590-C | 2012 | N/A | C580Y | -T------ | GGC |
| 103 | PD1165-C | 2012 | NHP4777 | C580Y | ---GCTCG | --- |
| 104 | PD1173-C | 2012 | NHP4851 | C580Y | ATTG---- | --- |
| 105 | PD1178-C | 2012 | NHP4856 | C580Y | ATTG---- | --- |
| 106 | PD0484-C | 2012 | TH004-039 | C580Y, E252Q | ATTG---- | --- |
| 107 | PD0485-C | 2012 | TH004-040 | A675V, C580Y, E252Q | ATTG---- | --- |
| 108 | PD0577-C | 2012 | N/A | C580Y | AT-G---- | --- |
| 109 | PD0578-C | 2012 | N/A | R539T | ATTG---- | --- |
| 110 | PD0583-C | 2012 | N/A | G449A | ATTG---- | GGC |
| 111 | PD0592-C | 2012 | N/A | P667A, P574L | ATTG---- | --- |
| 112 | PD0662-C | 2012 | N/A | R539T | ATTGCTC- | --- |
| 113 | PD0664-C | 2012 | N/A | R539T | ATTGC-C- | --- |
| 114 | PD0482-C | 2012 | TH004-037 | C580Y, E252Q | ATTG---- | --- |
| 115 | PD0483-C | 2012 | TH004-038 | E252Q | ATTG---- | --- |
| 116 | PD0806-C | 2012 | NHP1490 | C580Y | ATTG---- | --- |
| 117 | PD0990-C | 2012 | NHP4109 | P527H | A-TG---- | --- |
| 118 | PD0528-C | 2012 | TH004-056 | P553L | ATTG---- | GGC |
| 119 | PD0531-C | 2012 | TH004-059 | C580Y, E252Q | ATTG---- | --- |
| 120 | PD0571-C | 2012 | TH004-117 | C580Y | ATTG---- | --- |
| 121 | PD0814-C | 2013 | NHP1499 | C580Y | ATTG---- | --- |
| 122 | PD0818-C | 2013 | NHP1503 | C580Y | ---G---- | --- |
| 123 | PD0819-C | 2013 | NHP1504 | R561H | --TG---- | --- |
| 124 | PD0821-C | 2013 | NHP1506 | C580Y, R561H | ATTG---- | --- |
| 125 | PD0823-C | 2013 | NHP1508 | R561H | ATTG---- | --- |
| 126 | PD0824-C | 2013 | NHP1509 | R561H | ATTG---- | --- |
| 127 | PD0825-C | 2013 | NHP1510 | C580Y | ATTG---- | --- |
| 128 | PD0828-C | 2013 | NHP1513 | R561H | ATTG---- | --- |
| 129 | PD0832-C | 2013 | NHP1517 | R561H | ATTG---- | --- |
| 130 | PD0872-C | 2013 | NHP2200 | G538V | ATTG---- | --- |
| 131 | PD0873-C | 2013 | NHP2201 | C580Y, G538V | ATTG---- | --- |
| 132 | PD0874-C | 2013 | NHP2202 | C580Y, G538V | ATTG---- | --- |
| 133 | PD0912-C | 2013 | NHP3056 | R515K | AT-G---- | --- |
| 134 | PD0926-C | 2013 | NHP3214 | E252Q | ATTG---- | --- |
| 135 | PD0928-C | 2013 | NHP3218 | P574L | ATTG---- | --- |
| 136 | PD0933-C | 2013 | NHP3226 | C580Y | ATTG---- | --- |
| 137 | PD1214-C | 2013 | NHP4898 | C580Y | --TGCTCG | --- |
| 138 | PD1226-C | 2013 | NHP4910 | C580Y | ATTG---- | --- |
| 139 | PD1228-C | 2013 | NHP4912 | P574L | ATTG---- | --- |
| 140 | PD1230-C | 2013 | NHP4915 | C580Y | ---GCTCG | --- |
| 141 | PD1236-C | 2013 | NHP4922 | C580Y | ---GCTCG | --- |
| 142 | PD1247-C | 2013 | NHP4933 | C580Y | ATTG---- | --- |
| 143 | PD1250-C | 2013 | NHP4937 | C580Y | ATTG---- | --- |
| 144 | PD1251-C | 2013 | NHP4939 | C580Y | --TGCTCG | --- |
| 145 | PD0875-C | 2013 | NHP2203 | R575K | ATTG---- | --- |
| 146 | PD0822-C | 2013 | NHP1507 | R561H | AT-G---- | --- |
| 147 | PD0941-C | 2007 | NHP4032 | E252Q | -------- | GGC |
| 148 | PD0943-C | 2007 | NHP4036 | K438N | -------- | --- |
| 149 | PD0946-C | 2007 | NHP4039 | P441L | -------- | --- |
| 150 | PD0871-C | 2008 | NHP2199 | E252Q | -------- | GGC |
| 151 | PD0887-C | 2008 | NHP3019 | P441L | -------- | --- |
| 152 | PD0898-C | 2008 | NHP3031 | A675V | -------- | --- |
| 153 | PD0911-C | 2008 | NHP3054 | E252Q | -------- | --- |
| 154 | PD0462-C | 2011 | TH004-005 | A675V | -------- | GGC |
| 155 | PD0464-C | 2011 | TH004-007 | G538V | -------- | --- |
| 156 | PD0466-C | 2011 | TH004-009 | R561H, G538V | -------- | --- |
| 157 | PD0468-C | 2011 | TH004-011 | P553L | -------- | --- |
| 158 | PD0470-C | 2011 | TH004-013 | G538V | -------- | --- |
| 159 | PD0471-C | 2011 | TH004-014 | A675V | -------- | --- |
| 160 | PD0480-C | 2011 | TH004-029 | A675V | -------- | --- |
| 161 | PD0498-C | 2011 | N/A | R539T | -------- | --- |
| 162 | PD0500-C | 2011 | N/A | C580Y | -------- | GGC |
| 163 | PD0501-C | 2011 | N/A | C580Y | -------- | GGC |
| 164 | PD0502-C | 2011 | N/A | R539T | -------- | --- |
| 165 | PD0506-C | 2011 | N/A | C580Y | -------- | GGC |
| 166 | PD0509-Cx | 2011 | N/A | C580Y | -------- | --- |
| 167 | PD0510-C | 2011 | N/A | C580Y | -------- | GGC |
| 168 | PD0512-C | 2011 | TH004-021 | A675V | -------- | --- |
| 169 | PD0514-C | 2011 | TH004-023 | A481V | -------- | --- |
| 170 | PD0515-C | 2011 | TH004-031 | C580Y | -------- | --- |
| 171 | PD0627-C | 2011 | TH004-017 | A675V | -------- | --- |
| 172 | PD0629-C | 2011 | TH004-028 | C580Y | -------- | --- |
| 173 | PD0648-C | 2011 | N/A | C580Y | -------- | GGC |
| 174 | PD0526-C | 2012 | TH004-054 | C580Y | -------- | --- |
| 175 | PD0496-C | 2012 | TH004-095 | C580Y | -------- | --- |
| 176 | PD0497-C | 2012 | TH004-098 | A675V | -------- | --- |
| 177 | PD0527-C | 2012 | TH004-055 | G538V | -------- | --- |
| 178 | PD0530-C | 2012 | TH004-058 | G538V | -------- | --- |
| 179 | PD0533-C | 2012 | TH004-062 | G538V | -------- | --- |
| 180 | PD0538-C | 2012 | TH004-067 | C580Y | -------- | --- |
| 181 | PD0539-C | 2012 | TH004-068 | G538V | -------- | --- |
| 182 | PD0540-C | 2012 | TH004-069 | C580Y | -------- | --- |
| 183 | PD0487-C | 2012 | TH004-042 | G538V | -------- | --- |
| 184 | PD0546-C | 2012 | TH004-077 | G538V | -------- | --- |
| 185 | PD0553-C | 2012 | TH004-096 | C580Y | -------- | --- |
| 186 | PD0554-C | 2012 | TH004-097 | C580Y | -------- | --- |
| 187 | PD0555-C | 2012 | TH004-099 | F614L | -------- | GGC |
| 188 | PD0562-C | 2012 | TH004-108 | R561H | -------- | --- |
| 189 | PD0564-C | 2012 | TH004-110 | P441L | -------- | --- |
| 190 | PD0565-C | 2012 | TH004-111 | R561H | -------- | --- |
| 191 | PD0569-C | 2012 | TH004-115 | G538V | -------- | --- |
| 192 | PD0572-C | 2012 | TH004-118 | C580Y | -------- | --- |
| 193 | PD0575-C | 2012 | N/A | R539T | -------- | --- |
| 194 | PD0576-C | 2012 | N/A | R539T | -------- | --- |
| 195 | PD0579-C | 2012 | N/A | R539T | -------- | --- |
| 196 | PD0580-C | 2012 | N/A | R539T | -------- | --- |
| 197 | PD0581-C | 2012 | N/A | P441L | -------- | --- |
| 198 | PD0591-C | 2012 | N/A | R539T | -------- | --- |
| 199 | PD0635-C | 2012 | TH004-060 | G538V | -------- | --- |
| 200 | PD0636-C | 2012 | TH004-073 | G538V | -------- | --- |
| 201 | PD0641-C | 2012 | TH004-107 | R561H | -------- | --- |
| 202 | PD0651-C | 2012 | N/A | C580Y | -------- | GGC |
| 203 | PD0658-C | 2012 | N/A | R539T | -------- | --- |
| 204 | PD0659-C | 2012 | N/A | R539T | -------- | --- |
| 205 | PD0663-C | 2012 | N/A | R539T | -------- | --- |
| 206 | PD0799-C | 2012 | NHP1445 | R561H | -------- | --- |
| 207 | PD0800-C | 2012 | NHP1477 | R561H | -------- | --- |
| 208 | PD0803-C | 2012 | NHP1487 | C580Y | -------- | --- |
| 209 | PD0804-C | 2012 | NHP1488 | C580Y | -------- | --- |
| 210 | PD0929-C | 2013 | NHP3219 | C580Y | -------- | --- |
| 211 | PD0931-C | 2013 | NHP3223 | G538V | -------- | --- |
| 212 | PD0876-C | 2013 | NHP2204 | R561H | -------- | --- |
| 213 | PD0877-C | 2013 | NHP2205 | R561H | -------- | GGC |
| 214 | PD0827-C | 2013 | NHP1512 | C580Y | -------- | --- |
| 215 | PD0809-C | 2013 | NHP1494 | P441L | -------- | --- |
| 216 | PD0816-C | 2013 | NHP1501 | C580Y | -------- | --- |
| 217 | PD0826-C | 2013 | NHP1511 | R561H | -------- | --- |
| 218 | PD0927-C | 2013 | NHP3215 | C580Y | -------- | --- |
| 219 | PD0667-C | 2013 | N/A | C580Y, R539T | -------- | GGC |
| 220 | PD0668-C | 2013 | N/A | R539T | -------- | --- |
| 221 | PD1253-C | 2001 | OHP0032 | WT | ATTGCTCG | --- |
| 222 | PD1277-C | 2001 | OHP0091 | WT | ATTGCTCG | --- |
| 223 | PD1278-C | 2001 | OHP0093 | WT | ATTGCTCG | GGC |
| 224 | PD1281-C | 2001 | OHP0097 | WT | ATTGCTCG | --- |
| 225 | PD1257-C | 2001 | OHP0047 | WT | ATTGCTCG | GGC |
| 226 | PD1261-C | 2001 | OHP0053 | WT | ATTGCTCG | GGC |
| 227 | PD1264-C | 2001 | OHP0057 | WT | ATTGCTCG | --- |
| 228 | PD1266-C | 2001 | OHP0065 | WT | ATTGCTCG | --- |
| 229 | PD1267-C | 2001 | OHP0069 | WT | ATTGCTCG | --- |
| 230 | PD1268-C | 2001 | OHP0072 | WT | ATTGCTCG | --- |
| 231 | PD1434-C | 2001 | OHP0570 | WT | ATTGCTCG | GGC |
| 232 | PD1284-C | 2001 | OHP0102 | WT | ACTGCTCG | --- |
| 233 | PD1285-C | 2001 | OHP0103 | WT | ATTGCTCG | GGC |
| 234 | PD1288-C | 2002 | OHP0113 | WT | ATTGCTCG | --- |
| 235 | PD1290-C | 2002 | OHP0120 | WT | ATTGCTCG | --- |
| 236 | PD1294-C | 2002 | OHP0129 | WT | ATTGCTCG | GGC |
| 237 | PD1451-C | 2002 | OHP0651 | WT | ATTGCTCG | GGC |
| 238 | PD1436-C | 2002 | OHP0598 | WT | ATTGCTCG | --- |
| 239 | PD1437-C | 2002 | OHP0604 | WT | ATTGCTCG | --- |
| 240 | PD1440-C | 2002 | OHP0634 | WT | ATTGCTCG | GGC |
| 241 | PD1301-C | 2002 | OHP0151 | WT | ATTGCTCG | --- |
| 242 | PD1296-C | 2002 | OHP0138 | WT | ATTGCTCG | --- |
| 243 | PD1307-C | 2002 | OHP0158 | WT | ATTGCTCG | GGC |
| 244 | PD1309-C | 2002 | OHP0160 | WT | ATTGCTCG | GGC |
| 245 | PD1311-C | 2002 | OHP0162 | WT | ATTGCTCG | GGC |
| 246 | PD1319-C | 2002 | OHP0170 | WT | ATTGCTCG | --- |
| 247 | PD1331-C | 2002 | OHP0184 | WT | ATTGCTCG | --- |
| 248 | PD1332-C | 2002 | OHP0185 | WT | ATTGCTCG | --- |
| 249 | PD1335-C | 2002 | OHP0190 | WT | ATTGCTCG | --- |
| 250 | PD1337-C | 2002 | OHP0193 | WT | ATTGCTCG | --- |
| 251 | PD1355-C | 2003 | OHP0226 | WT | ATTGCTCG | --- |
| 252 | PD1357-C | 2003 | OHP0230 | WT | ATTGCTCG | --- |
| 253 | PD1360-C | 2003 | OHP0235 | WT | ATTGCTCG | GGC |
| 254 | PD1361-C | 2003 | OHP0236 | WT | ATTGCTCG | --- |
| 255 | PD1365-C | 2003 | OHP0242 | WT | ATTGCTCG | --- |
| 256 | PD1366-C | 2003 | OHP0243 | WT | ATTGCTCG | --- |
| 257 | PD1379-C | 2003 | OHP0266 | WT | ATTGCTCG | GGC |
| 258 | PD1380-C | 2003 | OHP0267 | WT | ATTGCTCG | GGC |
| 259 | PD1388-C | 2003 | OHP0284 | WT | ATTGCTCG | --- |
| 260 | PD1478-C | 2003 | OHP0779 | WT | ATTGCTCG | --- |
| 261 | PD1484-C | 2003 | OHP0812 | WT | ATTGCTCG | --- |
| 262 | PD1491-C | 2003 | OHP0852 | WT | ATTGCTCG | --- |
| 263 | PD1493-C | N/A | N/A | WT | ATTGCTCG | --- |
| 264 | PD1494-C | N/A | N/A | WT | ATTGCTCG | GGC |
| 265 | PD1501-C | 2004 | OHP1502 | WT | ATTGCTCG | --- |
| 266 | PD1502-C | 2004 | OHP1503 | WT | ATTGCTCG | GGC |
| 267 | PD1503-C | 2004 | OHP1504 | WT | ATTGCTCG | --- |
| 268 | PD1504-C | 2004 | OHP1505 | WT | ATTGCTCG | GGC |
| 269 | PD1512-C | 2004 | OHP1516 | WT | ATTGCTCG | --- |
| 270 | PD1399-C | 2004 | OHP0298 | WT | ATTGCTCG | --- |
| 271 | PD1400-C | 2004 | OHP0299 | WT | ATTGCTCG | GGC |
| 272 | PD1405-C | 2004 | OHP0308 | WT | ATTGCTCG | GGC |
| 273 | PD1422-C | 2004 | OHP0346 | WT | ATTGCTCG | --- |
| 274 | PD1409-C | 2004 | OHP0315 | WT | ATTGCTCG | --- |
| 275 | PD1412-C | 2004 | OHP0321 | WT | ATTGCTCG | GGC |
| 276 | PD0948-C | 2007 | NHP4042 | WT | ATTGCTCG | GGC |
| 277 | PD0949-C | 2007 | NHP4044 | WT | ATTGCTCG | --- |
| 278 | PD0883-C | 2007 | NHP3009 | WT | ATTGCTCG | --- |
| 279 | PD0841-C | 2008 | NHP2019 | WT | ATTGCTCG | GGC |
| 280 | PD0851-C | 2008 | NHP2042 | WT | ATTGCTCG | --- |
| 281 | PD1087-C | 2008 | NHP4268 | WT | ATTGCTCG | --- |
| 282 | PD1076-C | 2008 | NHP4254 | WT | ATTGCTCG | GGC |
| 283 | PD1077-C | 2008 | NHP4255 | WT | ATTGCTCG | GG- |
| 284 | PD1079-C | 2008 | NHP4257 | WT | ATTGCTCG | --- |
| 285 | PD1105-C | 2008 | NHP4288 | WT | ATTGCTCG | --- |
| 286 | PD1110-C | 2008 | NHP4296 | WT | ATTGCTCG | --- |
| 287 | PD1124-C | 2008 | NHP4323 | WT | ATTGCTCG | GGC |
| 288 | PD1125-C | 2008 | NHP4324 | WT | ATTGCTCG | --- |
| 289 | PD0892-C | 2008 | NHP3024 | WT | ATTGCTCG | --- |
| 290 | PD0901-C | 2008 | NHP3039 | WT | ATTGCTCG | --- |
| 291 | PD0904-C | 2008 | NHP3042 | WT | ATTGCTCG | --- |
| 292 | PD0905-C | 2008 | NHP3043 | WT | ATTGCTCG | GGC |
| 293 | PD0955-C | 2008 | NHP4053 | WT | ATTGCTCG | --- |
| 294 | PD0958-C | 2008 | NHP4060 | WT | ATTGCTCG | --- |
| 295 | PD0967-C | 2008 | NHP4079 | WT | ATTGCTCG | --- |
| 296 | PD1012-C | 2008 | NHP4146 | WT | ATTGCTCG | --- |
| 297 | PD0978-C | 2008 | NHP4092 | WT | ATTGCTCG | --- |
| 298 | PD0994-C | 2008 | NHP4113 | WT | ATTGCTCG | --- |
| 299 | PD1072-C | 2008 | NHP4249 | WT | ATTGCTCG | --- |
| 300 | PD1045-C | 2008 | NHP4205 | WT | ATTGCTCG | --- |
| 301 | PD1052-C | 2008 | NHP4218 | WT | ATTGCTCG | --- |
| 302 | PD1054-C | 2008 | NHP4220 | WT | ATTGCTCG | --- |
| 303 | PD1060-C | 2008 | NHP4228 | WT | ATTGCTCG | GGC |
| 304 | PD0855-C | 2008 | NHP2052 | WT | ATTGCTCG | --- |
| 305 | PD0859-C | 2008 | NHP2056 | WT | ATTGCTCG | --- |
| 306 | PD0915-C | 2008 | NHP3060 | WT | ATTGCTCG | --- |
| 307 | PD1000-C | 2008 | NHP4126 | WT | ATTGCTCG | --- |
| 308 | PD1008-C | 2008 | NHP4139 | WT | ATTGCTCG | GGC |
| 309 | PD0995-C | 2008 | NHP4115 | WT | ATTGCTCG | --- |
| 310 | PD0996-C | 2008 | NHP4116 | WT | ATTGCTCG | --- |
| 311 | PD1016-C | 2008 | NHP4153 | WT | ATTGCTCG | --- |
| 312 | PD1017-C | 2008 | NHP4158 | WT | ATTGCTCG | --- |
| 313 | PD1018-C | 2008 | NHP4159 | WT | ATTGCTCG | --- |
| 314 | PD1020-C | 2008 | NHP4163 | WT | ATTGCTCG | --- |
| 315 | PD1024-C | 2008 | NHP4170 | WT | ATTGCTCG | --- |
| 316 | PD1025-C | 2008 | NHP4171 | WT | ATTGCTCG | --- |
| 317 | PD1029-C | 2008 | NHP4179 | WT | ATTGCTCG | --- |
| 318 | PD1037-C | 2008 | NHP4193 | WT | ATTGCTCG | --- |
| 319 | PD1038-C | 2008 | NHP4194 | WT | ATTGCTCG | --- |
| 320 | PD1065-C | 2008 | NHP4236 | WT | ATTGCTCG | --- |
| 321 | PD1069-C | 2008 | NHP4244 | WT | ATTGCTCG | --- |
| 322 | PD0730-C | 2008 | NHP1037 | WT | ATTGCTCG | --- |
| 323 | PD0754-C | 2008 | NHP1076 | WT | ATTGCTCG | GGC |
| 324 | PD0755-C | 2008 | NHP1078 | WT | ATTGCTCG | --- |
| 325 | PD0760-C | 2008 | NHP1085 | WT | ATTGCTCG | --- |
| 326 | PD0765-C | 2008 | NHP1092 | WT | ATTGCTCG | --- |
| 327 | PD0772-C | 2008 | NHP1100 | WT | ATTGCTCG | GGC |
| 328 | PD0836-C | 2008 | NHP2011 | WT | ATTGCTCG | --- |
| 329 | PD0838-C | 2008 | NHP2015 | WT | ATTGCTCG | --- |
| 330 | PD1135-C | 2009 | NHP4435 | WT | ATTGCTCG | --- |
| 331 | PD1149-C | 2010 | NHP4587 | WT | ATTGCTCG | GGC |
| 332 | PD0459-Cx | 2011 | TH004-002 | WT | ATTGCTCG | --- |
| 333 | PD0475-C | 2011 | TH004-081 | WT | ATTGCTCG | GGC |
| 334 | PD0490-C | 2011 | TH004-086 | WT | ATTGCTCG | --- |
| 335 | PD0493-C | 2011 | TH004-092 | WT | ATTGCTCG | GGC |
| 336 | PD0507-C | 2011 | N/A | WT | ATTGCTCG | --- |
| 337 | PD0638-C | 2011 | TH004-083 | WT | ATTGCTCG | --- |
| 338 | PD0543-C | 2012 | TH004-072 | WT | ATTGCTCG | --- |
| 339 | PD0525-C | 2012 | TH004-053 | WT | ATTGCTCG | --- |
| 340 | PD0536-C | 2012 | TH004-065 | WT | ATTGCTCG | GGC |
| 341 | PD0570-C | 2012 | TH004-116 | WT | ATTGCTCG | --- |
| 342 | PD0585-CX | 2012 | N/A | WT | ATTGCTCG | GG- |
| 343 | PD0494-C | 2012 | TH004-093 | WT | ATTGCTCG | --- |
| 344 | PD1189-C | 2013 | NHP4868 | WT | ATTGCTCG | --- |
| 345 | PD0660-C | 2013 | N/A | WT | ATTGCTCG | --- |
| 346 | PD1255-C | 2001 | OHP0044 | WT | ATTG---- | GGC |
| 347 | PD1256-C | 2001 | OHP0046 | WT | ATTG---- | GGC |
| 348 | PD1262-C | 2001 | OHP0054 | WT | ATTG---- | --- |
| 349 | PD1273-C | 2001 | OHP0085 | WT | ATTGCT-- | --- |
| 350 | PD1283-C | 2001 | OHP0100 | WT | ATTG---- | --- |
| 351 | PD1291-C | 2002 | OHP0121 | WT | ATTG---- | --- |
| 352 | PD1300-C | 2002 | OHP0150 | WT | ATTG---- | --- |
| 353 | PD1304-C | 2002 | OHP0154 | WT | ATTG---- | --- |
| 354 | PD1464-C | 2002 | OHP0716 | WT | ATTG---- | --- |
| 355 | PD1310-C | 2002 | OHP0161 | WT | ATTG---- | --- |
| 356 | PD1328-C | 2002 | OHP0181 | WT | ATTG---- | GGC |
| 357 | PD1347-C | 2003 | OHP0215 | WT | ATTG---- | --- |
| 358 | PD1387-C | 2003 | OHP0283 | WT | ATTG---- | --- |
| 359 | PD1390-C | 2003 | OHP0286 | WT | ATTGC--- | --- |
| 360 | PD1487-C | 2003 | OHP0827 | WT | --TG---- | --- |
| 361 | PD1413-C | 2004 | OHP0322 | WT | A-TG---- | --- |
| 362 | PD1407-C | 2004 | OHP0313 | WT | ATTG---- | --- |
| 363 | PD1428-C | 2004 | OHP0355 | WT | ATTG---- | --- |
| 364 | PD1427-C | 2004 | OHP0354 | WT | ATTG---- | --- |
| 365 | PD0950-C | 2007 | NHP4045 | WT | ----CTCG | --- |
| 366 | PD0781-C | 2008 | NHP1110 | WT | ATTG---- | GGC |
| 367 | PD0776-C | 2008 | NHP1104 | WT | ATTG---- | GGC |
| 368 | PD1032-C | 2008 | NHP4183 | WT | ATTG---- | GGC |
| 369 | PD0919-C | 2008 | NHP3069 | WT | A-TG---- | --- |
| 370 | PD0971-C | 2008 | NHP4083 | WT | ATTGCTC- | --- |
| 371 | PD0998-C | 2008 | NHP4123 | WT | ATTG---- | --- |
| 372 | PD1009-C | 2008 | NHP4140 | WT | --TG---- | --- |
| 373 | PD1013-C | 2008 | NHP4147 | WT | ATTG---- | --- |
| 374 | PD1022-C | 2008 | NHP4165 | WT | ATTG---- | --- |
| 375 | PD1043-C | 2008 | NHP4203 | WT | -TTG---- | --- |
| 376 | PD1064-C | 2008 | NHP4233 | WT | A--GCTCG | --- |
| 377 | PD1073-C | 2008 | NHP4250 | WT | A-TGCTCG | --- |
| 378 | PD1082-C | 2008 | NHP4260 | WT | ATTG---- | --- |
| 379 | PD1083-C | 2008 | NHP4261 | WT | ATTG---- | --- |
| 380 | PD1111-C | 2008 | NHP4300 | WT | ATTG---- | --- |
| 381 | PD1099-C | 2008 | NHP4282 | WT | ATTG---- | --- |
| 382 | PD1133-C | 2008 | NHP4363 | WT | ATTG---- | GGC |
| 383 | PD0856-C | 2008 | NHP2053 | WT | ATTGCTC- | GGC |
| 384 | PD0865-C | 2008 | NHP2064 | WT | ATTG---- | --- |
| 385 | PD0869-C | 2008 | NHP2072 | WT | ATTG---- | GGC |
| 386 | PD0728-C | 2008 | NHP1035 | WT | ATTG---- | --- |
| 387 | PD0733-C | 2008 | NHP1051 | WT | ATTG---- | --- |
| 388 | PD0722-C | 2008 | NHP1027 | WT | A------- | --- |
| 389 | PD0724-C | 2008 | NHP1031 | WT | A-T----- | --- |
| 390 | PD0726-C | 2008 | NHP1033 | WT | ATT----- | --- |
| 391 | PD0735-C | 2008 | NHP1053 | WT | AT------ | --- |
| 392 | PD0740-C | 2008 | NHP1059 | WT | ----CTCG | GGC |
| 393 | PD0762-C | 2008 | NHP1088 | WT | ----CTCG | --- |
| 394 | PD0782-C | 2008 | NHP1111 | WT | ----CTCG | --- |
| 395 | PD0839-C | 2008 | NHP2017 | WT | ----CTCG | --- |
| 396 | PD0846-C | 2008 | NHP2026 | WT | A-T----- | --- |
| 397 | PD0889-C | 2008 | NHP3021 | WT | AT------ | --- |
| 398 | PD0891-C | 2008 | NHP3023 | WT | ----CTCG | --- |
| 399 | PD0906-C | 2008 | NHP3044 | WT | ATT-CTCG | --- |
| 400 | PD0907-C | 2008 | NHP3045 | WT | ATTG---- | --- |
| 401 | PD1033-C | 2008 | NHP4188 | WT | ATTG---- | --- |
| 402 | PD0767-C | 2008 | NHP1095 | WT | ATTG---- | --- |
| 403 | PD1147-C | 2009 | NHP4574 | WT | ---GCTCG | GGC |
| 404 | PD1139-C | 2009 | NHP4465 | WT | ATTG---- | --- |
| 405 | PD0790-C | 2010 | NHP1279 | WT | ----CTCG | GGC |
| 406 | PD1150-C | 2010 | NHP4668 | WT | A-TG---- | --- |
| 407 | PD0469-C | 2011 | TH004-012 | WT | ATTGCTC- | --- |
| 408 | PD0489-C | 2011 | TH004-084 | WT | ATTG---- | --- |
| 409 | PD1162-C | 2011 | NHP4758 | WT | ATTG---- | --- |
| 410 | PD1157-C | 2011 | NHP4747 | WT | ATTG---- | --- |
| 411 | PD0567-C | 2012 | TH004-113 | WT | --TGCTCG | --- |
| 412 | PD0574-C | 2012 | TH004-120 | WT | ATTG---- | --- |
| 413 | PD1179-C | 2012 | NHP4857 | WT | ATTG---- | --- |
| 414 | PD0522-C | 2012 | TH004-049 | WT | -TTG---- | --- |
| 415 | PD0541-C | 2012 | TH004-070 | WT | ATTG---- | --- |
| 416 | PD0532-C | 2012 | TH004-061 | WT | -T------ | --- |
| 417 | PD0547-C | 2012 | TH004-078 | WT | ----CTCG | --- |
| 418 | PD0813-C | 2013 | NHP1498 | WT | ATTG---- | --- |
| 419 | PD0831-C | 2013 | NHP1516 | WT | AT-G---- | GGC |
| 420 | PD1217-C | 2013 | NHP4901 | WT | ATTG---- | GGC |
| 421 | PD0935-C | 2007 | NHP4020 | WT | -------- | --- |
| 422 | PD0936-C | 2007 | NHP4023 | WT | -------- | --- |
| 423 | PD0717-C | 2007 | NHP1016 | WT | -------- | --- |
| 424 | PD0718-C | 2007 | NHP1019 | WT | -------- | --- |
| 425 | PD0834-C | 2007 | NHP2006 | WT | -------- | --- |
| 426 | PD0881-C | 2007 | NHP3007 | WT | -------- | --- |
| 427 | PD0882-C | 2007 | NHP3008 | WT | -------- | GGC |
| 428 | PD0884-C | 2007 | NHP3011 | WT | -------- | GGC |
| 429 | PD0885-C | 2007 | NHP3012 | WT | -------- | --- |
| 430 | PD0937-C | 2007 | NHP4024 | WT | -------- | --- |
| 431 | PD0938-C | 2007 | NHP4025 | WT | -------- | --- |
| 432 | PD0939-C | 2007 | NHP4028 | WT | -------- | --- |
| 433 | PD0942-C | 2007 | NHP4035 | WT | -------- | GGC |
| 434 | PD0944-C | 2007 | NHP4037 | WT | -------- | --- |
| 435 | PD0947-C | 2007 | NHP4041 | WT | -------- | --- |
| 436 | PD0766-C | 2008 | NHP1094 | WT | -------- | GGC |
| 437 | PD0771-C | 2008 | NHP1099 | WT | -------- | --- |
| 438 | PD0774-C | 2008 | NHP1102 | WT | -------- | --- |
| 439 | PD0777-C | 2008 | NHP1105 | WT | -------- | GGC |
| 440 | PD0785-C | 2008 | NHP1132 | WT | -------- | --- |
| 441 | PD0786-C | 2008 | NHP1137 | WT | -------- | --- |
| 442 | PD0758-C | 2008 | NHP1083 | WT | -------- | --- |
| 443 | PD0761-C | 2008 | NHP1087 | WT | -------- | GGC |
| 444 | PD0835-C | 2008 | NHP2009 | WT | -------- | --- |
| 445 | PD0837-C | 2008 | NHP2012 | WT | -------- | --- |
| 446 | PD0840-C | 2008 | NHP2018 | WT | -------- | --- |
| 447 | PD0843-C | 2008 | NHP2021 | WT | -------- | --- |
| 448 | PD0847-C | 2008 | NHP2037 | WT | -------- | --- |
| 449 | PD0848-C | 2008 | NHP2038 | WT | -------- | GGC |
| 450 | PD0852-C | 2008 | NHP2047 | WT | -------- | --- |
| 451 | PD0853-C | 2008 | NHP2049 | WT | -------- | --- |
| 452 | PD0854-C | 2008 | NHP2050 | WT | -------- | --- |
| 453 | PD0858-C | 2008 | NHP2055 | WT | -------- | --- |
| 454 | PD0860-C | 2008 | NHP2058 | WT | -------- | GGC |
| 455 | PD0861-C | 2008 | NHP2059 | WT | -------- | --- |
| 456 | PD0862-C | 2008 | NHP2060 | WT | -------- | GGC |
| 457 | PD0863-C | 2008 | NHP2061 | WT | -------- | --- |
| 458 | PD0864-C | 2008 | NHP2062 | WT | -------- | GGC |
| 459 | PD0747-C | 2008 | NHP1067 | WT | -------- | --- |
| 460 | PD0748-C | 2008 | NHP1068 | WT | -------- | --- |
| 461 | PD0749-C | 2008 | NHP1069 | WT | -------- | --- |
| 462 | PD0750-C | 2008 | NHP1070 | WT | -------- | --- |
| 463 | PD0886-C | 2008 | NHP3018 | WT | -------- | --- |
| 464 | PD0890-C | 2008 | NHP3022 | WT | -------- | --- |
| 465 | PD0894-C | 2008 | NHP3027 | WT | -------- | --- |
| 466 | PD0895-C | 2008 | NHP3028 | WT | -------- | GGC |
| 467 | PD0900-C | 2008 | NHP3036 | WT | -------- | --- |
| 468 | PD0903-C | 2008 | NHP3041 | WT | -------- | --- |
| 469 | PD0727-C | 2008 | NHP1034 | WT | -------- | --- |
| 470 | PD0908-C | 2008 | NHP3047 | WT | -------- | GGC |
| 471 | PD0909-C | 2008 | NHP3050 | WT | -------- | --- |
| 472 | PD0910-C | 2008 | NHP3052 | WT | -------- | --- |
| 473 | PD0914-C | 2008 | NHP3059 | WT | -------- | GGC |
| 474 | PD0916-C | 2008 | NHP3061 | WT | -------- | --- |
| 475 | PD0918-C | 2008 | NHP3063 | WT | -------- | GGC |
| 476 | PD0721-C | 2008 | NHP1025 | WT | -------- | --- |
| 477 | PD0729-C | 2008 | NHP1036 | WT | -------- | GGC |
| 478 | PD0734-C | 2008 | NHP1052 | WT | -------- | --- |
| 479 | PD0741-C | 2008 | NHP1060 | WT | -------- | GGC |
| 480 | PD0742-C | 2008 | NHP1061 | WT | -------- | GGC |
| 481 | PD0745-C | 2008 | NHP1064 | WT | -------- | --- |
| 482 | PD0778-C | 2008 | NHP1106 | WT | -------- | GGC |
| 483 | PD1086-C | 2008 | NHP4265 | WT | -------- | GGC |
| 484 | PD1117-C | 2008 | NHP4316 | WT | -------- | --- |
| 485 | PD0920-C | 2009 | NHP3072 | WT | -------- | --- |
| 486 | PD0795-C | 2010 | NHP1301 | WT | -------- | GGC |
| 487 | PD0923-C | 2010 | NHP3128 | WT | -------- | GGC |
| 488 | PD0458-Cx | 2011 | TH004-001 | WT | -------- | --- |
| 489 | PD0461-C | 2011 | TH004-004 | WT | -------- | --- |
| 490 | PD0463-Cx | 2011 | TH004-006 | WT | -------- | --- |
| 491 | PD0465-Cx | 2011 | TH004-008 | WT | -------- | --- |
| 492 | PD0467-C | 2011 | TH004-010 | WT | -------- | --- |
| 493 | PD0472-C | 2011 | TH004-015 | WT | -------- | --- |
| 494 | PD0474-C | 2011 | TH004-019 | WT | -------- | --- |
| 495 | PD0476-C | 2011 | TH004-082 | WT | -------- | --- |
| 496 | PD0511-C | 2011 | TH004-020 | WT | -------- | --- |
| 497 | PD0550-C | 2011 | TH004-085 | WT | -------- | --- |
| 498 | PD0626-C | 2011 | TH004-016 | WT | -------- | --- |
| 499 | PD0801-C | 2012 | NHP1485 | WT | -------- | --- |
| 500 | PD0495-C | 2012 | TH004-094 | WT | -------- | --- |
| 501 | PD0535-C | 2012 | TH004-064 | WT | -------- | --- |
| 502 | PD0558-C | 2012 | TH004-103 | WT | -------- | --- |
| 503 | PD0559-C | 2012 | TH004-104 | WT | -------- | --- |
| 504 | PD0561-C | 2012 | TH004-106 | WT | -------- | GGC |
| 505 | PD0582-C | 2012 | N/A | WT | -------- | --- |
| 506 | PD0586-C | 2012 | N/A | WT | -------- | GGC |
| 507 | PD0589-C | 2012 | N/A | WT | -------- | --- |
| 508 | PD0640-C | 2012 | TH004-102 | WT | -------- | --- |
| 509 | PD0925-C | 2012 | NHP3213 | WT | -------- | --- |
| 510 | PD0833-C | 2013 | NHP2004 | WT | -------- | --- |
| 511 | PD0817-C | 2013 | NHP1502 | WT | -------- | --- |
| 512 | PD0930-C | 2013 | NHP3221 | WT | -------- | --- |
| 513 | PD0934-C | 2013 | NHP3227 | WT | -------- | --- |

***Supplementary Table 5*. Bivariate statistical analysis on the shortlisted isolates by referring to genes PF3D7_0200300 and PF3D7_0223300, as well as the K13 mutation status of the isolates and the year of sample collection.**

- Table provided separately as excel file.

Supplementary Figures


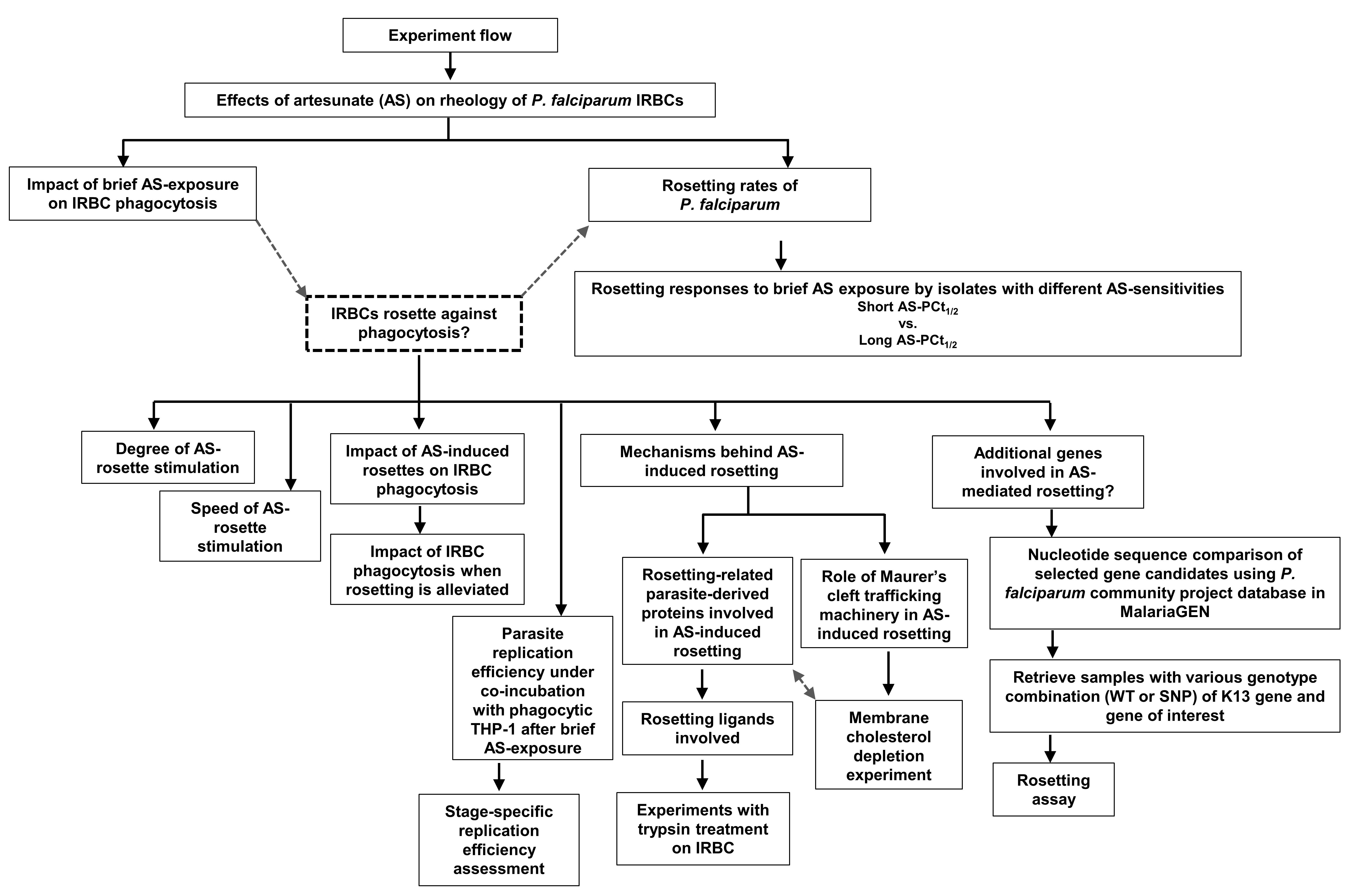


***Supplementary Figure 1*. Experiment workflow of this study.**

**
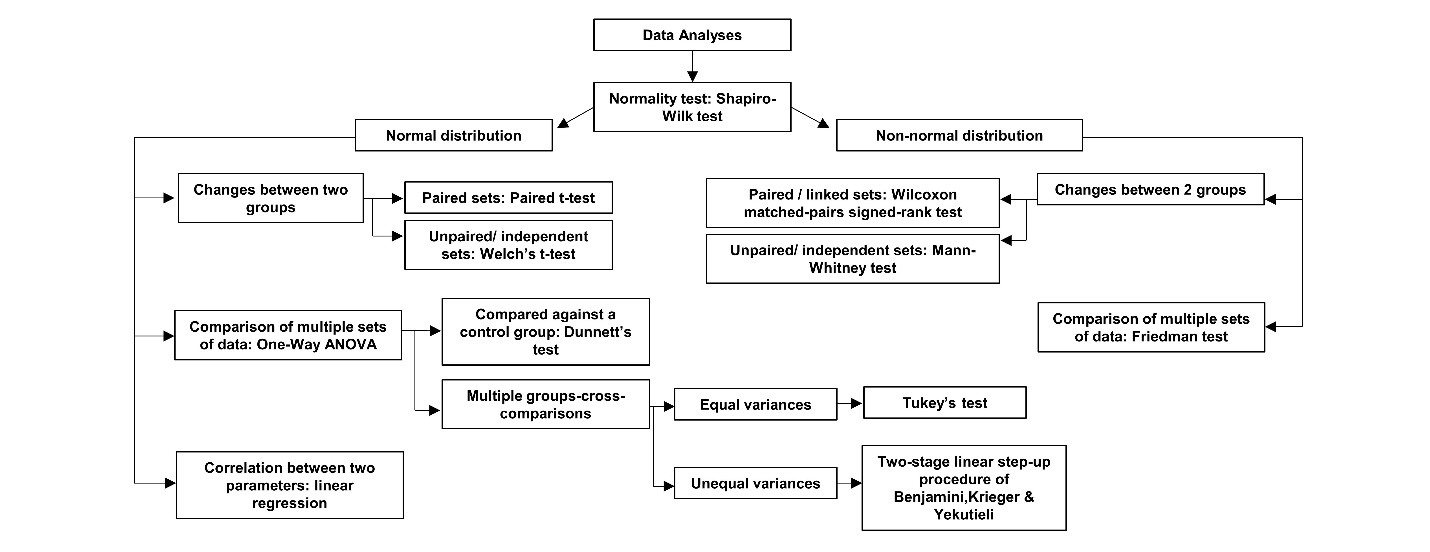
**

***Supplementary Figure 2*. Guidelines on types of statistical tests used based on the dataset collected.**


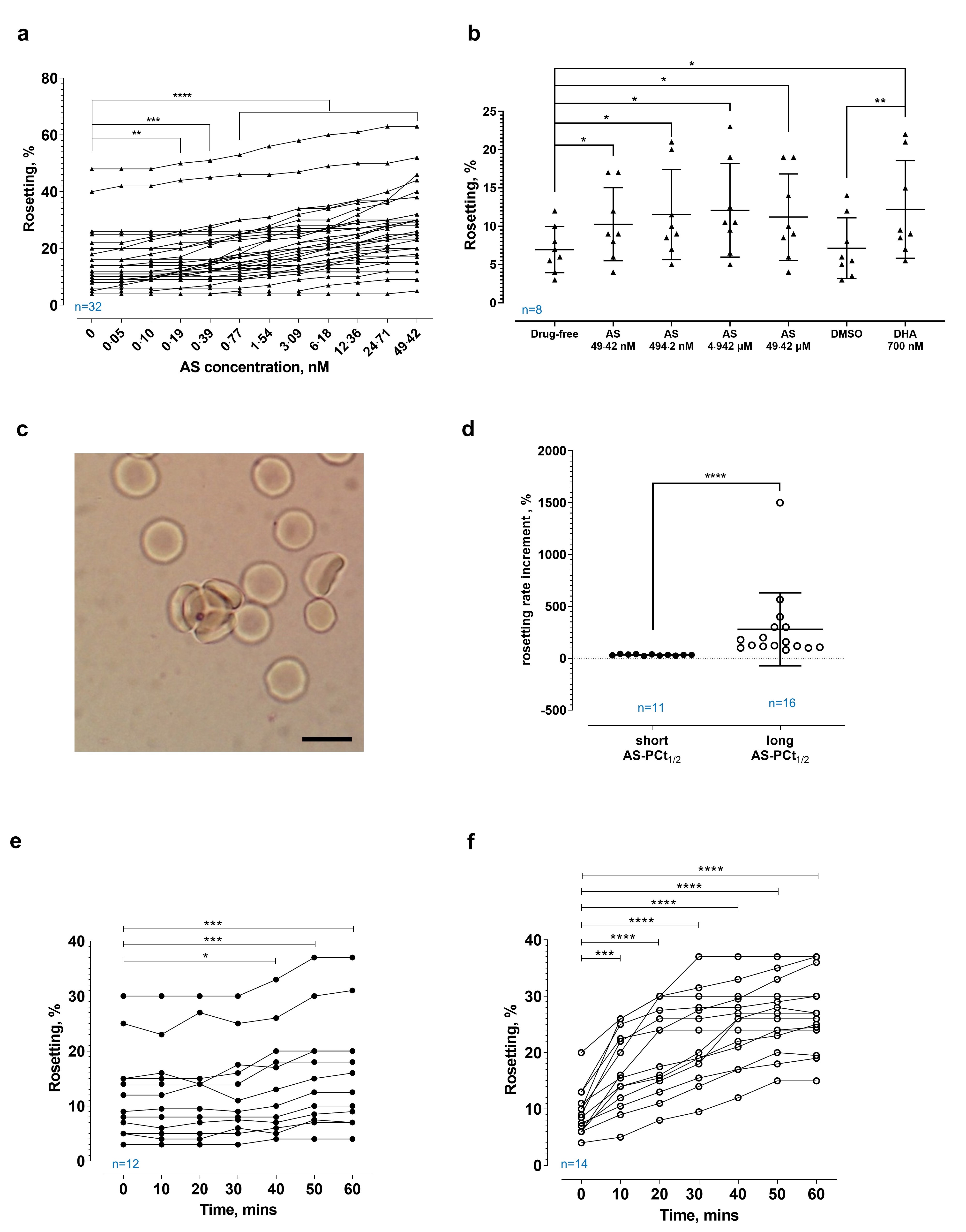


***Supplementary Figure 3*. Effects of drug exposure on rosetting. (a)** Effects of brief exposure of AS (0 – 49·42 nM) to rosetting of IRBC. Dunnett’s test revealed rosette-stimulation at 0.19 nM (P = 0·0051), 0·39 nM (P = 0·0001), 0·77 through 49·42 nM (P < 0·0001). **(b)** Rosetting rates of eight laboratory-adapted *P. falciparum* after brief exposure to AS of different high concentrations, DHA (700 nM) and DMSO (control; solvent of DHA). One-way ANOVA with Tukey’s test was performed for cross-group comparisons. When compared against the untreated setting, rosetting rates were significantly increased after exposure to AS of 49·42 nM, 494·2 nM, 4·942 µM and 49·42 µM (adjusted P = 0·0210, 0·0440, 0·0455, 0·0375 respectively). When compared with the control (DMSO), rosetting rates were significantly higher after exposure to DHA 700 nM (adjusted P = 0·0095). Untreated versus DMSO groups were not significantly different from each other (adjusted P = 0·9995). The degree of rosette-stimulation by 49·42 nM AS was similar to those of 494·2 nM (P = 0·3404), 4·942 µM (P = 0·2075), 49·42 µM (P = 0·1972), and DHA (P = 0·1745). **(c)** Rosette formed by the late ring-IRBC of a *P. falciparum* isolate from the long AS-PCt_1/2_ group under AS exposure; scale bar 10 µm. **(d)** Ring stage-rosette-stimulation by AS (mean and S.D. shown) in short AS-PCt_1/2_ and long AS-PCt_1/2_ groups, P < 0·0001, U = 0 (Mann-Whitney test). **(e)** In short AS-PCt_1/2_, rosette-stimulation occurred at the 40^th^, 50^th^ and 60^th^ minutes (Dunnett’s test P = 0·0271, 0·0002 and 0·0002 respectively). **(f)** In long AS-PCt_1/2_, rosette-stimulation occurred at the 10^th^ minute (P = 0·0001), and 20^th^ through 60^th^ minute (P <0·0001).


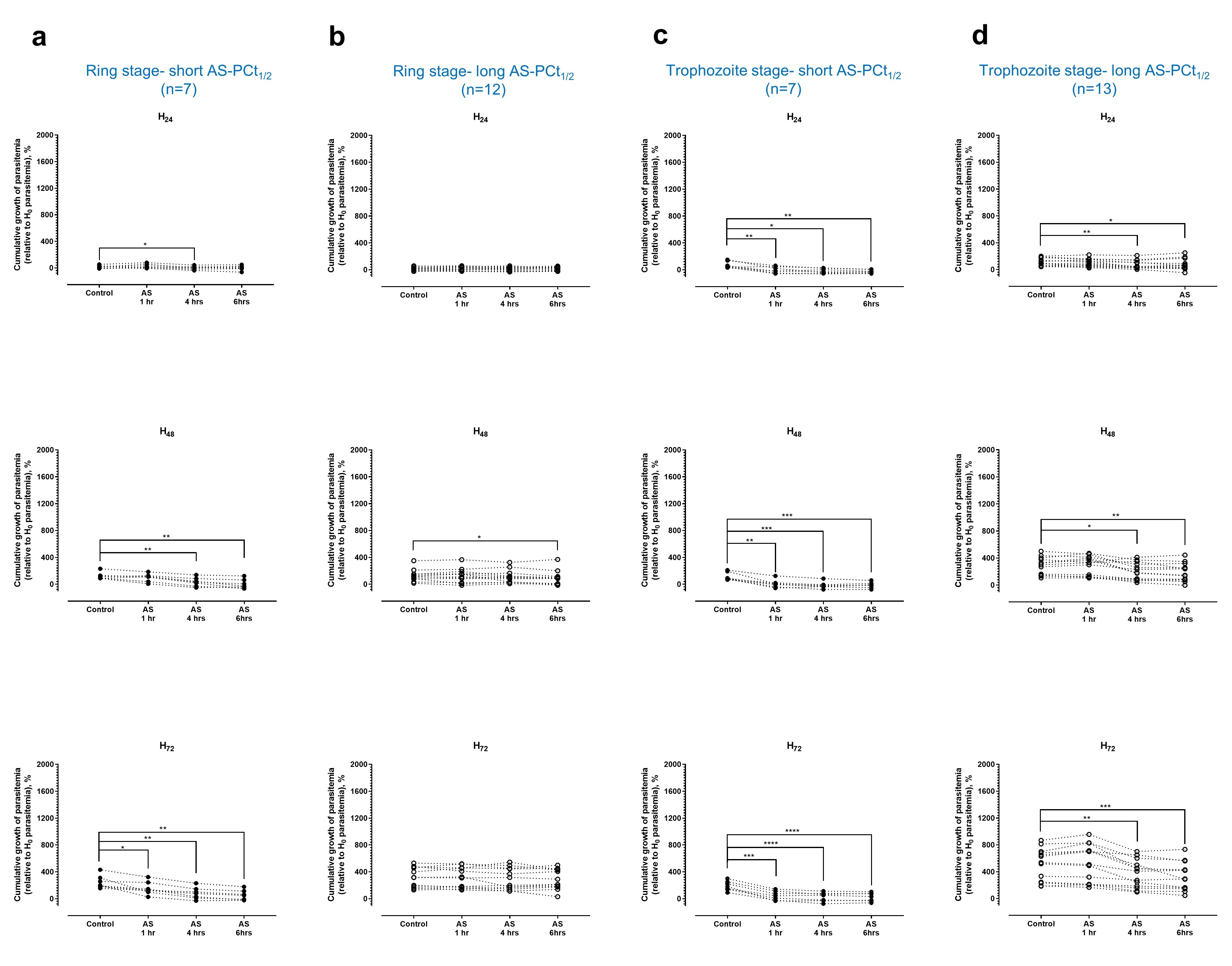


***Supplementary Figure 4*. Effects of AS-mediated rosetting on survival of ring and trophozoite stages post-AS exposure. (a-d)** Ring and trophozoite stages were exposed to AS for different durations prior to removal of drug from the system. The parasites were then cultured *in vitro*. Parasitemia growth at different hours (H)-post drug removal [H_24_ (top), H_48_ (middle) and H_72_ (bottom)] were monitored. The plots represent ring stages with short AS-PCt_1/2_ **(a)** and long AS-PCt_1/2_ **(b)**; trophozoites with short AS-PCt_1/2_ **(c)** and long AS-PCt_1/2_ **(d)**. Parasitemia of drug-exposed groups were compared with those of control using One-way ANOVA with Dunnett’s test. In **(a)**, at H_24_, P = 0·7091, 0·0423 and 0·5028 for 1 hr-, 4 hrs- and 6 hrs-exposure groups, respectively. At H_48_, P = 0·2403, 0·0017 and 0·0013 for groups 1hr, 4hrs and 6hrs, respectively. At H_72_, all drug exposed groups (1 hr, 4 hrs and 6 hrs) showed lower parasitemia growth than the control (P = 0·0429, 0·0044 and 0·0014 respectively). In **(b)** At H_24_, P = 0·9433, 0·8000 and 0·9990 for groups 1 hr, 4 hrs and 6 hrs, respectively. At H_48_, P = 0·9992, 0·2852 and 0·0310 for groups 1hr, 4hrs and 6hrs, respectively. At H_72_, P = 0·9994, 0·5879 and 0·3239 for 1 hr, 4 hrs and 6 hrs, respectively. For **(c)** At H_24_, P = 0·0099, 0·0149 and 0·0067 for 1hr, 4 hrs and 6hrs, respectively. At H_48_, P = 0·0016, 0·0009 and 0·0008, respectively. At H_72_, P = 0·0001 for all groups. In **(d)** At H_24_, P = 0·7336, 0·0018 and 0·0346 for 1hr, 4 hrs and 6 hrs, respectively. At H_48_, P = 0·9327, 0·013 and 0·0042 for groups 1 hr, 4 hrs and 6 hrs, respectively. At H_72_, P = 0·3997, 0·0017 and 0·0009 for groups 1 hr, 4 hrs and 6 hrs, respectively.


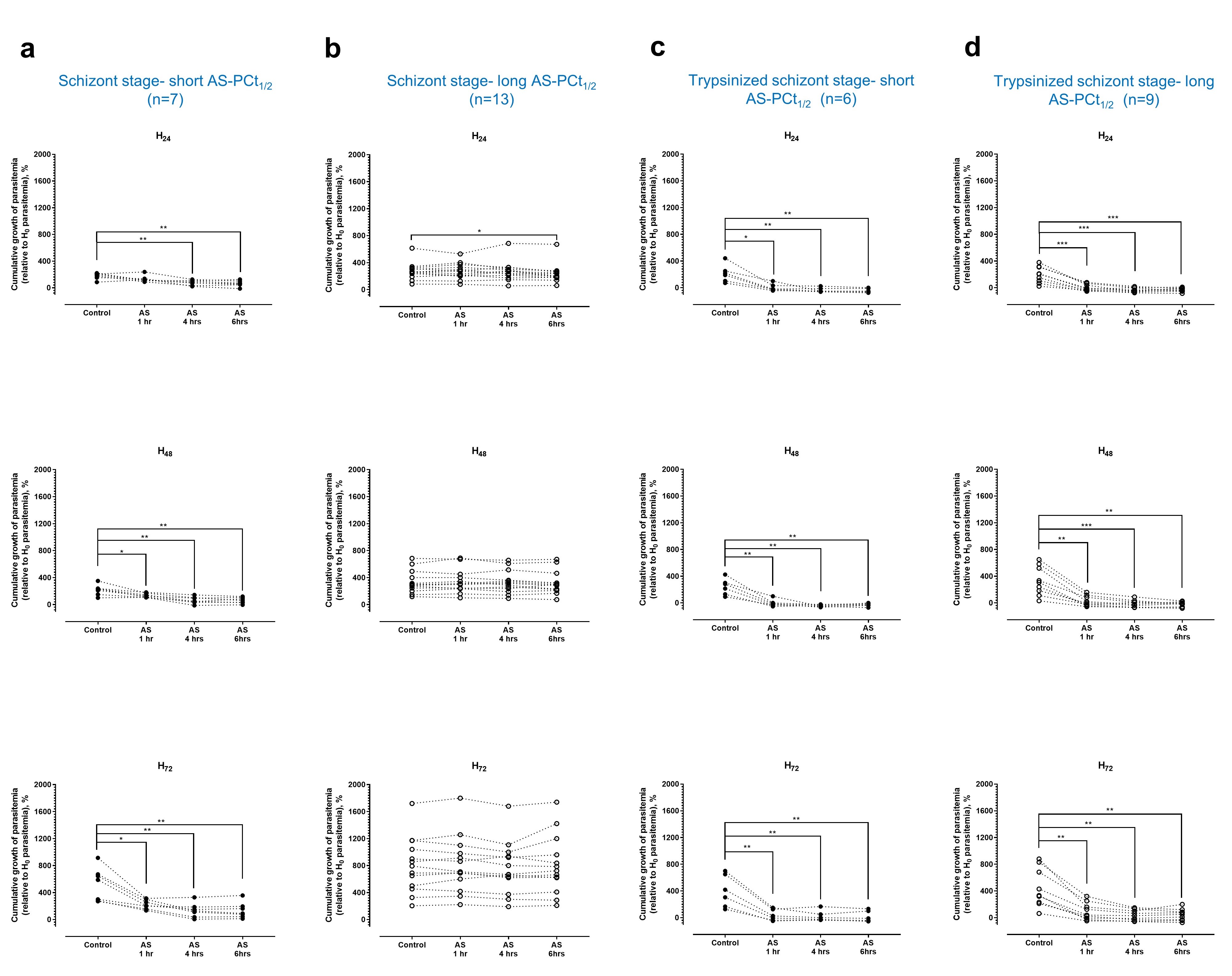


***Supplementary Figure 5*. Effect of AS-mediated rosetting on survival of schizonts post-AS exposure. (a-d)** The plots represent schizonts with short AS-PCt_1/2_ **(a)** and long AS-PCt_1/2_ **(b)**, trypsinised schizonts with short AS-PCt_1/2_ **(c)** and long AS-PCt_1/2_ **(d)**. Schizonts were exposed to AS for different durations prior to drug removal from the system and subsequent *in vitro* cultivation. Parasitemia growth at H_24_ (top), H_48_ (middle) and H_72_ (bottom) were monitored. Parasitemia of drug-exposed groups were compared with those of the control using One-way ANOVA with Dunnett’s test. In **(a)**, At H_24_, P = 0·2090, 0·0026 and 0·0032 for 1 hr-, 4 hrs- and 6 hrs-exposure groups, respectively. At H_48_, P = 0·0274, 0·0028 and 0·0032 for 1 hr-, 4 hrs- and 6 hrs-exposure groups, respectively. At H_72_, P = 0·013, 0·0032 and 0·0041 for 1 hr-, 4 hrs- and 6 hrs-exposure groups, respectively. In **(b)**, At H_24_, P = 0·9920, 0·9564 and 0·0246 for 1 hr-, 4 hrs- and 6 hrs-exposure groups, respectively. At H_48_, P = 0·8077, 0·9985 and 0·6050 for 1 hr-, 4 hrs- and 6 hrs-exposure groups, respectively. At H_72_, P = 0·9277, 0·1746 and 0·9999 for 1 hr-, 4 hrs- and 6 hrs-exposure groups, respectively. For **(c)** At H_24_, P = 0·0135, 0·0077 and 0·0088 for 1 hr-, 4 hrs- and 6 hrs-exposure groups, respectively. At H_48_, P = 0·0056, 0·0063 and 0·0053 for 1 hr-, 4 hrs- and 6 hrs-exposure groups, respectively. At H_72_, P = 0·0063, 0·0097 and 0·0062 for 1 hr-, 4 hrs- and 6 hrs-exposure groups, respectively. For **(d)** At H_24_, P = 0·0003, 0·0008 and 0·0007 for 1 hr-, 4 hrs- and 6 hrs-exposure groups, respectively. At H_48_, P = 0·0011, 0·0010 and 0·0014 for 1 hr-, 4 hrs- and 6 hrs-exposure groups, respectively. At H_72_, P = 0·0012, 0·0014 and 0·0015 for 1 hr-, 4 hrs- and 6 hrs-exposure groups, respectively.


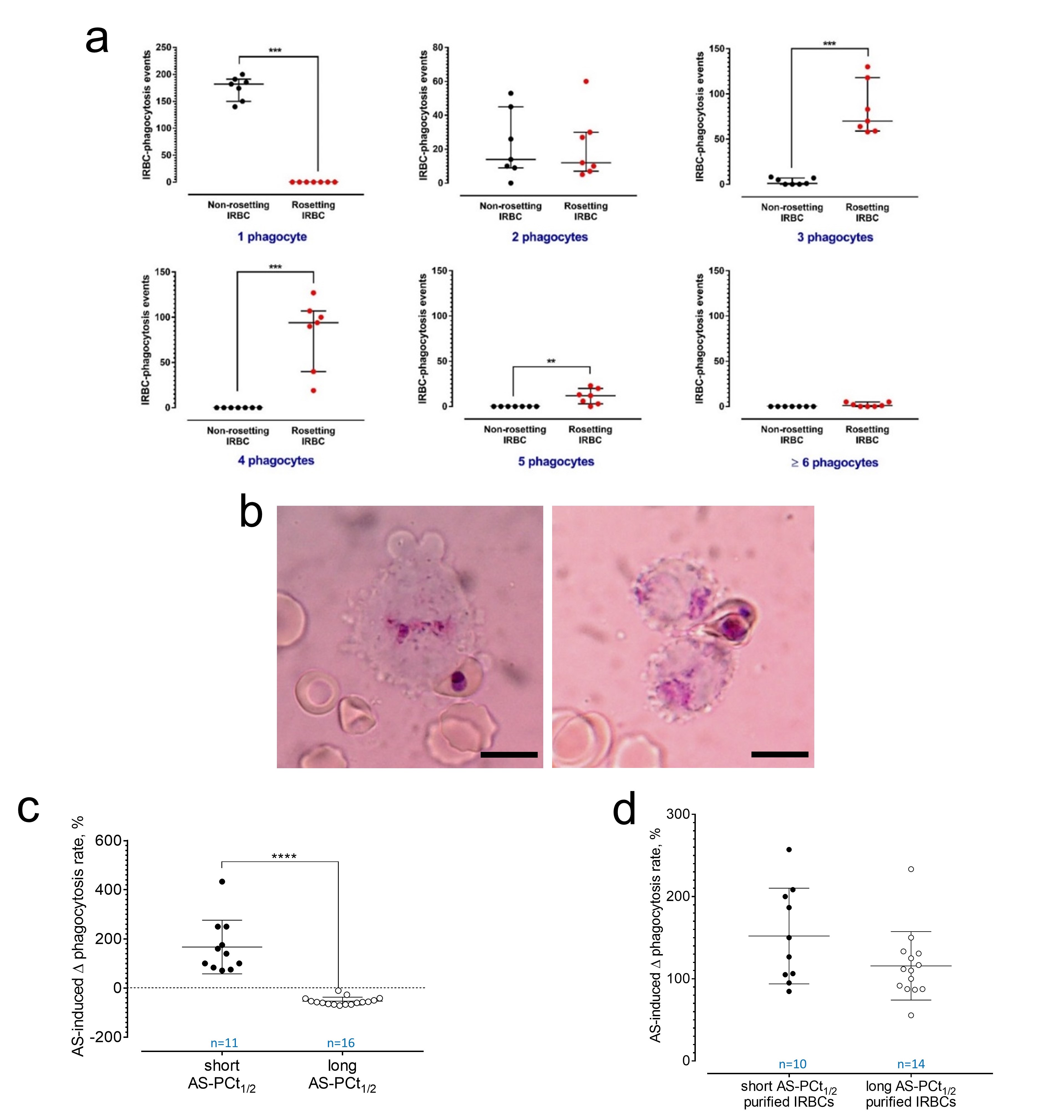


***Supplementary Figure 6*.** **Rosettes, AS and IRBC phagocytosis. (a)** Occurrence of IRBC-phagocytosis involving different numbers of THP-1. Experiments were conducted on seven laboratory-adapted parasite lines. Error bars represent median and interquartile range. Mann-Whitney test was conducted. None of the phagocytosis events observed in rosetting IRBC group (n=1400) involved only one phagocyte whereas majority of the phagocytosis events in the non-rosetting IRBC group were mediated by one phagocyte (P = 0·0006). For phagocytosis events involving two phagocytes, no significant difference found between the non-rosetting and rosetting groups (P > 0·9999). The incidents involving three phagocytes were significantly higher in rosetting IRBC group than the non-rosetting group (P = 0·0006). No phagocytosis event involving four or more phagocytes were found in the non-rosetting group, whereas many phagocytosis events in the rosetting group were found to be involved of four phagocytes (P = 0·0006) and five phagocytes (P = 0·0047). Only a few (n=13) phagocytosis events in the rosetting group were found to be involved of at least six phagocytes; no significant difference was found between the two groups in this category (P = 0·0699). **(b)** Phagocytosis of a non-rosetting IRBC (left) and a rosette (right). Scale bar 10 µm. **(c)** Comparison of AS-induced phagocytosis changes between the short AS-PCt_1/2_ and long AS-PCt_1/2_ groups, mean and S.D. shown. Mann-Whitney test P < 0·0001. **(d)** Differences in AS-induced purified IRBC phagocytosis changes between isolates with short and long AS-PCt_1/2_ (mean and S.D. shown). Mann-Whitney P = 0·1767.


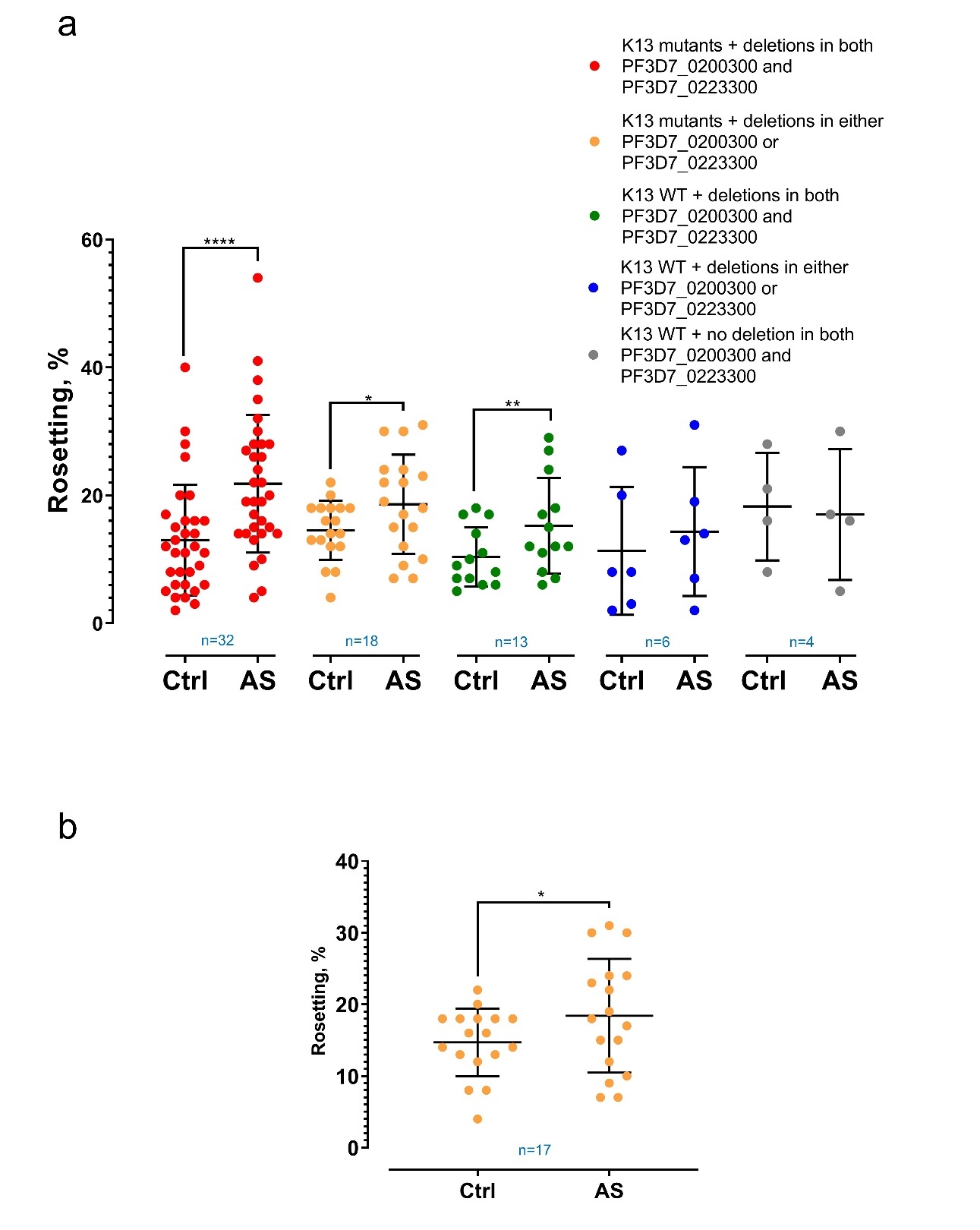


***Supplementary Figure 7*.** **AS-mediated rosetting in *P. falciparum* with different genotype combinations of K13, PF3D7_0223300 and PF3D7_0200300.** **(a)** Rosetting rates of isolates from all experiment groups after AS exposure for one hour. Each combination was represented by a distinct color. From Wilcoxon test, AS stimulated rosetting of K13 mutants with deletions in PF3D7_0200300 and PF3D7_0223300 (P < 0·0001), K13 mutants with deletions in either of the two genes (P = 0·0133), and K13 WT with deletions in both genes (P = 0·0032). K13 WT with deletions in either one of the two genes of interest and K13 WT without deletion in both genes did not show significant changes in rosetting post-AS exposure (P = 0·3125 and 0·5 respectively). **(b)** AS mediated rosetting by K13 mutants with deletions only in PF3D7_0223300; Wilcoxon test P = 0·0252.
