# Supplementary material for: Plasmodium falciparum rosetting protects schizonts against artemisinin
Source: eBioMedicine. 2021 Nov 5;73:103680. doi: 10.1016/j.ebiom.2021.103680 (PMC8586750; doi:10.1016/j.ebiom.2021.103680)
Supplement: Supplementary file 3 [file mmc3.docx]

**Captions for supplementary materials**

***Supplementary Table 1*. Reagents and Tools Table.**

***Supplementary Table 2*.** **Genotype combination of genes PF3D7_1343700 (coded for K13), PF3D7_0200300 and PF3D7_0223300 (both coded for parts of PfEMP1 exon 2) among the isolates recruited for experiments in Figure 3c, Supplementary Figures 7a and b.**

***Supplementary Table 3*. The 140 *P. falciparum* genes shortlisted for SNPs comparison.**

***Supplementary Table 4*. Sequenced Thai *P. falciparum* isolates retrieved from MalariaGEN database.**

***Supplementary Table 5*. Bivariate statistical analysis on the shortlisted isolates by referring to genes PF3D7_0200300 and PF3D7_0223300, as well as the K13 mutation status of the isolates and the year of sample collection.**

***Supplementary Figure 1*. Experiment workflow of this study.**

***Supplementary Figure 2*. Guidelines on types of statistical tests used based on the dataset collected.**

***Supplementary Figure 3*. Effects of drug exposure on rosetting. (a)** Effects of brief exposure of AS (0 – 49·42 nM) to rosetting of IRBC. Dunnett’s test revealed rosette-stimulation at 0.19 nM (P = 0·0051), 0·39 nM (P = 0·0001), 0·77 through 49·42 nM (P < 0·0001). **(b)** Rosetting rates of eight laboratory-adapted *P. falciparum* after brief exposure to AS of different high concentrations, DHA (700 nM) and DMSO (control; solvent of DHA). One-way ANOVA with Tukey’s test was performed for cross-group comparisons. When compared against the untreated setting, rosetting rates were significantly increased after exposure to AS of 49·42 nM, 494·2 nM, 4·942 µM and 49·42 µM (adjusted P = 0·0210, 0·0440, 0·0455, 0·0375 respectively). When compared with the control (DMSO), rosetting rates were significantly higher after exposure to DHA 700 nM (adjusted P = 0·0095). Untreated versus DMSO groups were not significantly different from each other (adjusted P = 0·9995). The degree of rosette-stimulation by 49·42 nM AS was similar to those of 494·2 nM (P = 0·3404), 4·942 µM (P = 0·2075), 49·42 µM (P = 0·1972), and DHA (P = 0·1745). **(c)** Rosette formed by the late ring-IRBC of a *P. falciparum* isolate from the long AS-PCt_1/2_ group under AS exposure; scale bar 10 µm. **(d)** Ring stage-rosette-stimulation by AS (mean and S.D. shown) in short AS-PCt_1/2_ and long AS-PCt_1/2_ groups, P < 0·0001, U = 0 (Mann-Whitney test). **(e)** In short AS-PCt_1/2_, rosette-stimulation occurred at the 40^th^, 50^th^ and 60^th^ minutes (Dunnett’s test P = 0·0271, 0·0002 and 0·0002 respectively). **(f)** In long AS-PCt_1/2_, rosette-stimulation occurred at the 10^th^ minute (P = 0·0001), and 20^th^ through 60^th^ minute (P <0·0001).

***Supplementary Figure 4*. Effects of AS-mediated rosetting on survival of ring and trophozoite stages post-AS exposure. (a-d)** Ring and trophozoite stages were exposed to AS for different durations prior to removal of drug from the system. The parasites were then cultured *in vitro*. Parasitemia growth at different hours (H)-post drug removal [H_24_ (top), H_48_ (middle) and H_72_ (bottom)] were monitored. The plots represent ring stages with short AS-PCt_1/2_ **(a)** and long AS-PCt_1/2_ **(b)**; trophozoites with short AS-PCt_1/2_ **(c)** and long AS-PCt_1/2_ **(d)**. Parasitemia of drug-exposed groups were compared with those of control using One-way ANOVA with Dunnett’s test. In **(a)**, at H_24_, P = 0·7091, 0·0423 and 0·5028 for 1 hr-, 4 hrs- and 6 hrs-exposure groups, respectively. At H_48_, P = 0·2403, 0·0017 and 0·0013 for groups 1hr, 4hrs and 6hrs, respectively. At H_72_, all drug exposed groups (1 hr, 4 hrs and 6 hrs) showed lower parasitemia growth than the control (P = 0·0429, 0·0044 and 0·0014 respectively). In **(b)** At H_24_, P = 0·9433, 0·8000 and 0·9990 for groups 1 hr, 4 hrs and 6 hrs, respectively. At H_48_, P = 0·9992, 0·2852 and 0·0310 for groups 1hr, 4hrs and 6hrs, respectively. At H_72_, P = 0·9994, 0·5879 and 0·3239 for 1 hr, 4 hrs and 6 hrs, respectively. For **(c)** At H_24_, P = 0·0099, 0·0149 and 0·0067 for 1hr, 4 hrs and 6hrs, respectively. At H_48_, P = 0·0016, 0·0009 and 0·0008, respectively. At H_72_, P = 0·0001 for all groups. In **(d)** At H_24_, P = 0·7336, 0·0018 and 0·0346 for 1hr, 4 hrs and 6 hrs, respectively. At H_48_, P = 0·9327, 0·013 and 0·0042 for groups 1 hr, 4 hrs and 6 hrs, respectively. At H_72_, P = 0·3997, 0·0017 and 0·0009 for groups 1 hr, 4 hrs and 6 hrs, respectively.

***Supplementary Figure 5*. Effect of AS-mediated rosetting on survival of schizonts post-AS exposure. (a-d)** The plots represent schizonts with short AS-PCt_1/2_ **(a)** and long AS-PCt_1/2_ **(b)**, trypsinised schizonts with short AS-PCt_1/2_ **(c)** and long AS-PCt_1/2_ **(d)**. Schizonts were exposed to AS for different durations prior to drug removal from the system and subsequent *in vitro* cultivation. Parasitemia growth at H_24_ (top), H_48_ (middle) and H_72_ (bottom) were monitored. Parasitemia of drug-exposed groups were compared with those of the control using One-way ANOVA with Dunnett’s test. In **(a)**, At H_24_, P = 0·2090, 0·0026 and 0·0032 for 1 hr-, 4 hrs- and 6 hrs-exposure groups, respectively. At H_48_, P = 0·0274, 0·0028 and 0·0032 for 1 hr-, 4 hrs- and 6 hrs-exposure groups, respectively. At H_72_, P = 0·013, 0·0032 and 0·0041 for 1 hr-, 4 hrs- and 6 hrs-exposure groups, respectively. In **(b)**, At H_24_, P = 0·9920, 0·9564 and 0·0246 for 1 hr-, 4 hrs- and 6 hrs-exposure groups, respectively. At H_48_, P = 0·8077, 0·9985 and 0·6050 for 1 hr-, 4 hrs- and 6 hrs-exposure groups, respectively. At H_72_, P = 0·9277, 0·1746 and 0·9999 for 1 hr-, 4 hrs- and 6 hrs-exposure groups, respectively. For **(c)** At H_24_, P = 0·0135, 0·0077 and 0·0088 for 1 hr-, 4 hrs- and 6 hrs-exposure groups, respectively. At H_48_, P = 0·0056, 0·0063 and 0·0053 for 1 hr-, 4 hrs- and 6 hrs-exposure groups, respectively. At H_72_, P = 0·0063, 0·0097 and 0·0062 for 1 hr-, 4 hrs- and 6 hrs-exposure groups, respectively. For **(d)** At H_24_, P = 0·0003, 0·0008 and 0·0007 for 1 hr-, 4 hrs- and 6 hrs-exposure groups, respectively. At H_48_, P = 0·0011, 0·0010 and 0·0014 for 1 hr-, 4 hrs- and 6 hrs-exposure groups, respectively. At H_72_, P = 0·0012, 0·0014 and 0·0015 for 1 hr-, 4 hrs- and 6 hrs-exposure groups, respectively.

***Supplementary Figure 6*.** **Rosettes, AS and IRBC phagocytosis. (a)** Occurrence of IRBC-phagocytosis involving different numbers of THP-1. Experiments were conducted on seven laboratory-adapted parasite lines. Error bars represent median and interquartile range. Mann-Whitney test was conducted. None of the phagocytosis events observed in rosetting IRBC group (n=1400) involved only one phagocyte whereas majority of the phagocytosis events in the non-rosetting IRBC group were mediated by one phagocyte (P = 0·0006). For phagocytosis events involving two phagocytes, no significant difference found between the non-rosetting and rosetting groups (P > 0·9999). The incidents involving three phagocytes were significantly higher in rosetting IRBC group than the non-rosetting group (P = 0·0006). No phagocytosis event involving four or more phagocytes were found in the non-rosetting group, whereas many phagocytosis events in the rosetting group were found to be involved of four phagocytes (P = 0·0006) and five phagocytes (P = 0·0047). Only a few (n=13) phagocytosis events in the rosetting group were found to be involved of at least six phagocytes; no significant difference was found between the two groups in this category (P = 0·0699). **(b)** Phagocytosis of a non-rosetting IRBC (left) and a rosette (right). Scale bar 10 µm. **(c)** Comparison of AS-induced phagocytosis changes between the short AS-PCt_1/2_ and long AS-PCt_1/2_ groups, mean and S.D. shown. Mann-Whitney test P < 0·0001. **(d)** Differences in AS-induced purified IRBC phagocytosis changes between isolates with short and long AS-PCt_1/2_ (mean and S.D. shown). Mann-Whitney P = 0·1767.

***Supplementary Figure 7*.** **AS-mediated rosetting in *P. falciparum* with different genotype combinations of K13, PF3D7_0223300 and PF3D7_0200300.** **(a)** Rosetting rates of isolates from all experiment groups after AS exposure for one hour. Each combination was represented by a distinct color. From Wilcoxon test, AS stimulated rosetting of K13 mutants with deletions in PF3D7_0200300 and PF3D7_0223300 (P < 0·0001), K13 mutants with deletions in either of the two genes (P = 0·0133), and K13 WT with deletions in both genes (P = 0·0032). K13 WT with deletions in either one of the two genes of interest and K13 WT without deletion in both genes did not show significant changes in rosetting post-AS exposure (P = 0·3125 and 0·5 respectively). **(b)** AS mediated rosetting by K13 mutants with deletions only in PF3D7_0223300; Wilcoxon test P = 0·0252.
